# Disproportionality analysis of adverse event signals associated with seven Alzheimer’s drugs: insights from the FAERS database

**DOI:** 10.3389/fphar.2026.1955230

**Published:** 2026-09-24

**Authors:** Kai Liu, Xiaohu Yang, Lingti Kong

**Affiliations:** 1 Department of Pharmacy, The First Affiliated Hospital of Bengbu Medical University, Bengbu, China; 2 School of Pharmacy, Bengbu Medical University, Bengbu, China; 3 State Key Laboratory of Neurology and Oncology Drug Development, Nanjing, China

**Keywords:** adverse drug events, alzheimer’s disease drugs, disproportionality analysis, FDA adverse event reporting system (FAERS), signal

## Abstract

**Objective:**

To compare adverse event reporting patterns for seven Alzheimer’s disease (AD) drugs (aducanumab, donanemab, lecanemab, donepezil, galantamine, rivastigmine, and memantine) using the FDA Adverse Event Reporting System (FAERS), and to explore potential reporting signals.

**Methods:**

FAERS reports from the first quarter of 2004 to the second quarter of 2026 were retrospectively analyzed. Adverse events were coded using MedDRA version 28.1 preferred terms (PTs) and system organ classes (SOCs). Disproportionality signals were detected using the reporting odds ratio (ROR), proportional reporting ratio (PRR), Bayesian confidence propagation neural network (BCPNN), and multi-item gamma-Poisson shrinker (MGPS). PT-level signals were additionally controlled using Benjamini–Hochberg false discovery rate correction. Drug-level comparisons, sex- and age-stratified analyses, time-to-onset (TTO) analysis, and Weibull distribution fitting were performed.

**Results:**

A total of 39,301 valid reports involving the seven AD drugs were included. Reports were more frequent among women (52.3%, n = 20,537) and patients aged ≥65 years. Anti-Aβ monoclonal antibodies showed concentrated reporting signals in nervous system disorders, with amyloid related imaging abnormalities (ARIA) related events as the core PT-level signal. Donanemab and lecanemab were also associated with prominent infusion-related reaction signals. Conventional symptomatic drugs demonstrated broader multi-system reporting patterns: donepezil and galantamine were mainly associated with cholinergic cardiovascular and fall-related signals; rivastigmine with administration-site reactions and fall-related events; and memantine with confusional state, agitation, dizziness, and somnolence. Several PT-level signals were not listed in the reviewed prescribing information and were annotated as potential reporting signals. Sex- and age-stratified analyses showed broadly consistent PT-level spectra. TTO analyses revealed heterogeneous onset patterns among different drug and adverse events.

**Conclusion:**

This study utilized FAERS data to compare adverse event reporting signals for seven anti-AD drugs. Distinct reporting profiles were observed across drugs, and several potential ADE signals, including potential unlabeled reporting signals, were identified. These findings may support pharmacovigilance surveillance and hypothesis generation, but should be interpreted as reporting associations rather than evidence of causality.

## 1 Introduction

Alzheimer’s disease (AD) is an insidious and progressive neurodegenerative disorder (Jack et al., 2024; Scheltens et al., 2021; Author Anonymous, 2024). With population aging worldwide, the number of individuals at risk for AD and related dementias is increasing substantially: global dementia cases are projected to increase from 57.4 million in 2019 to 152.8 million by 2050 (GBD, 2019, 2022). Pharmacological intervention remains a key approach for delaying disease progression and managing both cognitive decline and neuropsychiatric symptoms. For several decades, systemic anti-AD treatment has primarily included acetylcholinesterase inhibitors (AChEIs), such as donepezil, rivastigmine, and galantamine, and memantine, an N-methyl-D-aspartate (NMDA) receptor antagonist. These conventional agents provide symptomatic benefit at specific disease stages (Shi et al., 2022).

As disease-modifying therapies have emerged, clinical treatment guidelines for AD continue to evolve. New-generation biologicals, represented by amyloid-β (Aβ)-targeting monoclonal antibodies, including aducanumab, lecanemab, and donanemab, are intended for intervention during the very early stages of disease to reduce cerebral Aβ plaques and slow clinical decline (Cummings et al., 2024; Sims et al., 2023; Swanson et al., 2021; van Dyck et al., 2023). Although these drugs may improve cognition or slow decline, they are also associated with clinically relevant safety concerns. AChEIs are known to increase central and peripheral cholinergic tone and can cause gastrointestinal cramping and nausea, as well as bradycardia, atrioventricular block, syncope, and a risk of falling (Ruangritchankul et al., 2021; Young et al., 2021). Anti-Aβ monoclonal antibodies are known to reduce amyloid plaques, although amyloid-related imaging abnormalities (ARIA) have limited their broader clinical application (Doran and Sawyer, 2024; Jeong et al., 2022; Salloway et al., 2022).

Existing pre-marketing studies are limited by sample size, follow-up duration, and participant homogeneity, thus reducing the detection of delayed or rare adverse events. Therefore, there is a critical need for post-marketing safety evaluation including large pharmacovigilance databases (Cutroneo et al., 2023; Potter et al., 2025). FAERS, the World Health Organization global database of individual case safety reports (VigiBase), and the European Union pharmacovigilance system (EudraVigilance) provide key data sources for the mining of signals on a large scale. Based on reports of suspected adverse drug reactions, signal detection can identify emerging medication safety risks and support the safer use of medications (Fusaroli et al., 2024; Li et al., 2026; Mouffak et al., 2021).

Although individual FAERS-based studies have described adverse event signals for selected anti-Aβ monoclonal antibodies, direct comparative characterization across both disease-modifying monoclonal antibodies and conventional symptomatic therapies remains limited (Hu et al., 2025; Huang et al., 2025; Kong et al., 2025; Xing et al., 2025; Yang et al., 2024). Moreover, comprehensive comparisons integrating subgroup analyses, PT-level temporal characteristics, and multiple signal detection algorithms remain scarce. On this basis, the present study used real-world data from the FAERS database to comparatively characterize adverse event reporting signals associated with seven current AD therapies, including three anti-Aβ monoclonal antibodies and four conventional symptomatic drugs. In particular, we aimed to provide a unified comparison of safety signals across mechanistically distinct drugs and to comprehensively evaluate reporting patterns through drug- and PT-level analyses, sex- and age-stratified analyses, time-to-onset characteristics, and multiple disproportionality algorithms. Our findings may contribute to post-marketing safety surveillance and support the rational selection of treatments and risk management (Hu et al., 2025; Huang et al., 2025; Kong et al., 2025).

## 2 Materials and methods

### 2.1 Data source

Data were extracted from the FDA Adverse Event Reporting System (FAERS) database covering the first quarter of 2004 through the second quarter of 2026. The FAERS data files were downloaded on 19 August 2026, which was defined as the database lock date for this analysis. The core FAERS modules were used, including DEMO for demographic and administrative information, DRUG for suspect and concomitant drug information, REAC for adverse event preferred terms, OUTC for patient outcomes, RPSR for report sources, THER for drug therapy start and end dates, and INDI for indication information. During data cleaning, a sequential de-duplication strategy was applied at the case level. For records sharing the same CASEID, the most recent report was retained according to FDA_DT; when multiple records had the same FDA_DT, the record with the largest PRIMARYID was retained. After case-level de-duplication, remaining duplicate PRIMARYIDs were further removed to ensure that each report was counted only once. The complete workflow for data extraction, cleaning, and de-duplication is shown in Figure 1.

**FIGURE 1:**
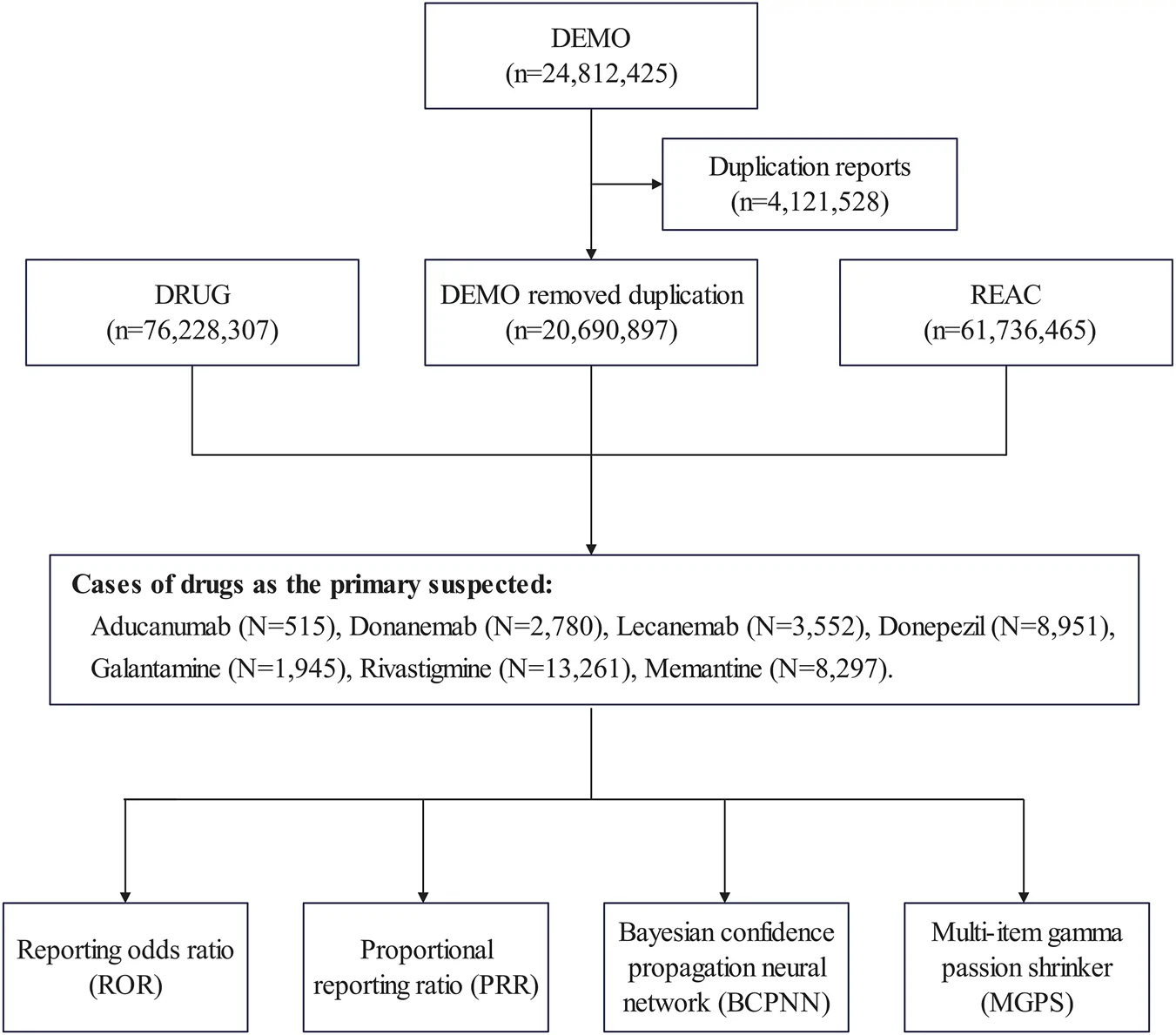
Flowchart showing data extraction from the FAERS database followed by disproportionality analysis for seven drugs used to treat Alzheimer’s Disease. Abbreviations: FAERS: the US Food and Drug Administration Adverse Event Reporting System.

### 2.2 Data filtering

Using the FAERS database, we screened for the generic names of three anti-Aβ monoclonal antibodies (aducanumab, lecanemab, and donanemab) and four conventional agents (donepezil, rivastigmine, galantamine, and memantine), together with their corresponding brand names (Aricept, Exelon, Razadyne, Namenda, Aduhelm, Leqembi, and Kisunla). Drug names were manually harmonized across generic names, brand names, and common spelling variants. We extracted records in which the drug was coded as the primary suspect (PS) drug in the ROLE field of the database. Adverse events were described and classified according to MedDRA version 28.1 preferred terms (PTs) and system organ classes (SOCs).

### 2.3 Statistical analysis

Signal detection was performed using four disproportionality analysis algorithms: reporting odds ratio (ROR), proportional reporting ratio (PRR), Bayesian confidence propagation neural network (BCPNN), and multi-item gamma-Poisson shrinker (MGPS). For each drug–PT pair, a 2 × 2 contingency table was constructed. Because these estimators are derived from the same table and are not independent, their predefined thresholds were jointly applied as a conservative signal filter. Signals were defined as positive only when all four algorithm-specific criteria were met (Table 1).

**TABLE 1:** Summary of the main algorithms used for signal detection.

Algorithm	Equation	Positive criteria
ROR	$ROR=\frac{a\times d}{b\times c}$ $95\%CI=e^{\ln\left(ROR\right)\pm 1.96\sqrt{\frac{1}{a}+\frac{1}{b}+\frac{1}{c}+\frac{1}{d}}}$	lower limit of 95%CI>1 a≥3
PRR	$PRR=\frac{a}{\left(a+b\right)}÷\frac{c}{\left(c+d\right)}$ $\chi^{2}=\frac{{\left(ad‐bc\right)}^{2}\times \left(a+b+c+d\right)}{\left(a+b\right)\left(c+d\right)\left(a+c\right)\left(d+b\right)}$	PRR≥2 $\chi^{2}\geq 4,a\geq 3$
BCPNN	$IC=\log_{2}a\left(a+b+c+d\right)\left(a+b\right)$ $IC025=e^{\ln\left(IC\right)‐1.96\sqrt{\frac{1}{a}+\frac{1}{b}+\frac{1}{c}+\frac{1}{d}}}$	IC025>0
MGPS	$EBGM=\frac{a\left(a+b+c+d\right)}{\left(a+c\right)\left(a+b\right)}$ $EBGM05=e^{\ln\left(EBGM\right)‐1.96\sqrt{\frac{1}{a}+\frac{1}{b}+\frac{1}{c}+\frac{1}{d}}}$	EBGM05≥2 N>0

Abbreviations: ROR: reporting odds ratio; PRR: proportional reporting ratio; BCPNN: bayesian confidence propagation neural network; MGPS: multi-item gamma-Poisson shrinker; IC: information component; EBGM: empirical Bayes geometric mean; a: number of reports arising from the suspect adverse event (AE) and suspect drug; b: number of reports arising from the suspect AE and all other drugs; c: number of reports arising from the suspect drug and other AEs; d: number of reports arising from other drugs and other AEs; CI: confidence interval; 2: chi-squared; IC025: lower limit of 95% two-sided CI of the IC; EBGM05: lower limit of 95% one-sided CI of EBG.

PT-level analyses were exploratory. Benjamini–Hochberg false discovery rate (FDR) correction was applied within each drug across all tested PTs, and main PT-level signals were required to meet both the four-algorithm criteria and the FDR-adjusted threshold. For sex-stratified analyses, only PTs positive in both female and male reports for the same drug were compared. Female-to-male reporting odds ratios with 95% confidence intervals were calculated from 2 × 2 tables using a 0.5 continuity correction. Fisher’s exact test was applied when any expected count was <5; Pearson’s chi-square test without continuity correction was applied when all expected counts were ≥5. P values were adjusted within each drug using the Benjamini–Hochberg method.

Time to onset (TTO) was defined as the interval between the first recorded therapy start date (drug first exposure) and the first reported adverse event occurrence. Reports with valid non-negative TTO values were included in descriptive and non-parametric analyses. TTO was summarized as median and interquartile range (IQR). Between-drug differences were assessed using the Kruskal–Wallis rank-sum test, followed by pairwise Wilcoxon rank-sum tests with Benjamini–Hochberg correction. Weibull fitting was performed by maximum likelihood estimation among reports with positive TTO values. The shape parameter β was used to classify onset patterns as early-failure (β < 1), random (β = 1), or wear-out (β > 1).

Analyses were performed using R version 4.5.1. Data processing used readxl, dplyr, purrr, tibble, stringr, writexl, and openxlsx; Weibull fitting used MASS; and visualization used ggplot2 and pheatmap. Categorical variables were summarized as frequencies and percentages, and continuous variables as mean ± standard deviation or median (IQR), as appropriate. All results were interpreted as reporting patterns rather than incidence or causality.

## 3 Results

### 3.1 Demographic characteristics

A total of 39,301 adverse event reports related to the seven target drugs were included in the final analysis. Table 2 summarizes demographic characteristics and report sources by drug. Conventional drugs accounted for higher report volumes, with rivastigmine having the largest number of reports (N = 13,261), followed by donepezil (N = 8,951) and memantine (N = 8,297). As clinical use expanded, lecanemab accumulated 3,552 reports, whereas fewer reports were available for donanemab (N = 2,780) and aducanumab (N = 515).

**TABLE 2:** Demographic characteristics of the adverse event reports for seven drugs used to treat Alzheimer’s disease.

Characteristic	Aducanumab (N = 515)	Donanemab (N = 2780)	Lecanemab (N = 3552)	Donepezil (N = 8951)	Galantamine (N = 1945)	Rivastigmine (N = 13261)	Memantine (N = 8297)	Overall (N = 39301)
Sex								
Female	258 (50.1%)	1348 (48.5%)	1874 (52.8%)	4539 (50.7%)	865 (44.5%)	7347 (55.4%)	4306 (51.9%)	20537 (52.3%)
Male	228 (44.3%)	837 (30.1%)	1325 (37.3%)	3157 (35.3%)	667 (34.3%)	5160 (38.9%)	3062 (36.9%)	14436 (36.7%)
Missing	29 (5.6%)	595 (21.4%)	353 (9.9%)	1255 (14.0%)	413 (21.2%)	754 (5.7%)	929 (11.2%)	4328 (11.0%)
Age (years)								
<18	0 (0.0%)	0 (0.0%)	0 (0.0%)	54 (0.6%)	0 (0.0%)	15 (0.1%)	56 (0.7%)	125 (0.3%)
18∼64.9	30 (5.8%)	120 (4.3%)	279 (7.9%)	644 (7.2%)	60 (3.1%)	312 (2.4%)	714 (8.6%)	2159 (5.5%)
65∼85	271 (52.6%)	1264 (45.5%)	2345 (66.0%)	4571 (51.1%)	779 (40.1%)	4345 (32.8%)	3866 (46.6%)	17441 (44.4%)
>85	15 (2.9%)	54 (1.9%)	88 (2.5%)	1542 (17.2%)	348 (17.9%)	1952 (14.7%)	1443 (17.4%)	5442 (13.8%)
Missing	199 (38.6%)	1342 (48.3%)	840 (23.6%)	2140 (23.9%)	758 (39.0%)	6637 (50.0%)	2218 (26.7%)	14134 (36.0%)
Reporter type								
Consumer	215 (41.7%)	1644 (59.1%)	664 (18.7%)	1399 (15.6%)	254 (13.1%)	6424 (48.4%)	3401 (41.0%)	14001 (35.6%)
Health-professional	114 (22.1%)	123 (4.4%)	379 (10.7%)	1215 (13.6%)	88 (4.5%)	258 (1.9%)	489 (5.9%)	2666 (6.8%)
Physician	177 (34.4%)	285 (10.3%)	1626 (45.8%)	2994 (33.4%)	698 (35.9%)	3444 (26.0%)	2042 (24.6%)	11266 (28.7%)
Pharmacist	6 (1.2%)	33 (1.2%)	89 (2.5%)	1094 (12.2%)	588 (30.2%)	787 (5.9%)	719 (8.7%)	3316 (8.4%)
Other	0 (0.0%)	0 (0.0%)	0 (0.0%)	1798 (20.1%)	264 (13.6%)	1518 (11.4%)	1298 (15.6%)	4878 (12.4%)
Missing	3 (0.6%)	695 (25.0%)	794 (22.4%)	451 (5.0%)	53 (2.7%)	830 (6.3%)	348 (4.2%)	3174 (8.1%)
Reporter country								
United States	477 (92.6%)	2460 (88.5%)	2805 (79.0%)	2082 (23.3%)	407 (20.9%)	2984 (22.5%)	2748 (33.1%)	13963 (35.5%)
Japan	10 (1.9%)	213 (7.7%)	585 (16.5%)	1265 (14.1%)	841 (43.2%)	1359 (10.2%)	300 (3.6%)	4573 (11.6%)
United Kingdom	1 (0.2%)	4 (0.1%)	4 (0.1%)	2315 (25.9%)	108 (5.6%)	386 (2.9%)	909 (11.0%)	3727 (9.5%)
Brazil	0 (0.0%)	6 (0.2%)	1 (0.0%)	63 (0.7%)	50 (2.6%)	2293 (17.3%)	8 (0.1%)	2421 (6.2%)
Other/Missing	27 (5.2%)	97 (3.5%)	157 (4.4%)	3226 (36.0%)	539 (27.7%)	6239 (47.0%)	4332 (52.2%)	14617 (37.2%)

In total, 52.3% (n = 20,537) of subjects were women, while 36.7% were men; 11.0% of all reports were missing sex information. The number of adverse event reports was higher among women than among men; this was consistent with the higher prevalence of AD among women. The reporting population was predominantly aged 65 years or older. By reporting country, the United States accounted for 35.5% of all reports, followed by Japan (11.6%) and the United Kingdom (9.5%).

### 3.2 Signal detection at the SOC level

Table 3 presents SOC-level signals that met the predefined positivity criteria for all four disproportionality algorithms. The complete SOC-level results were provided in Supplementary Tables S1–S7. To visualize differences in signal strength across SOCs, a heatmap of SOC-level signal intensity based on ROR values was generated (Figure 2). Analysis revealed substantial differences in adverse event profiles across the seven anti-AD drugs; however, all drugs exhibited core positive signals in nervous system disorders. Monoclonal antibodies showed the most concentrated signal pattern. For aducanumab, donanemab, and lecanemab, nervous system disorders showed consistently strong signals across all four algorithms. For aducanumab, nervous system disorders showed ROR = 12.58 (95% CI: 11.18–14.16), PRR = 6.40 (χ^2^ = 2930.25), EBGM = 6.40 (EBGM05 = 5.68), and IC = 2.68 (IC025 = 2.52). The corresponding values were ROR = 7.25 (95% CI: 6.89–7.63), PRR = 4.77 (χ^2^ = 8033.55), EBGM = 4.76 (EBGM05 = 4.53), and IC = 2.25 (IC025 = 2.18) for donanemab and ROR = 7.19 (95% CI: 6.87–7.54), PRR = 4.74 (χ^2^ = 9419.82), EBGM = 4.74 (EBGM05 = 4.52), and IC = 2.24 (IC025 = 2.18) for lecanemab. In contrast, conventional symptomatic drugs showed broader multi-system distributions. Donepezil showed relatively strong signals for cardiac, psychiatric, and nervous system disorders. For cardiac disorders, ROR = 3.82 (95% CI: 3.66–3.98), PRR = 3.56 (χ^2^ = 4559.09), EBGM = 3.56 (EBGM05 = 3.41), and IC = 1.83 (IC025 = 1.77). Galantamine also showed a prominent signal for cardiac disorders, with ROR = 2.77 (95% CI: 2.49–3.09), PRR = 2.65 (χ^2^ = 373.56), EBGM = 2.65 (EBGM05 = 2.38), and IC = 1.41 (IC025 = 1.24). Memantine showed prominent signals for psychiatric and nervous system disorders. For psychiatric disorders, ROR = 3.65 (95% CI: 3.53–3.78), PRR = 3.19 (χ^2^ = 6152.60), EBGM = 3.19 (EBGM05 = 3.08), and IC = 1.67 (IC025 = 1.62). Although rivastigmine had the highest numbers of reports for general disorders and administration site conditions (8,912 reports) and nervous system disorders (7,751 reports), the SOC-level profile of this drug reflected a high-frequency distribution rather than highly concentrated disproportionality enrichment.

**TABLE 3:** Positive signals at the System Organ Class (SOC) level for seven drugs used to treat Alzheimer’s disease.

SOCs	Frequency	ROR (95%Cl)	PRR (χ^2^)	EBGM(EBGM05)	IC(IC025)
Aducanumab					
Nervous system disorders	590	12.58 (11.18–14.16)	6.4 (2930.25)	6.4 (5.68)	2.68 (2.52)
Donanemab					
Vascular disorders	352	2.8 (2.51–3.11)	2.69 (382.88)	2.69 (2.42)	1.43 (1.26)
Nervous system disorders	2476	7.25 (6.89–7.63)	4.77 (8033.55)	4.76 (4.53)	2.25 (2.18)
Immune system disorders	164	2.39 (2.05–2.8)	2.36 (129.7)	2.36 (2.02)	1.24 (1)
Donepezil					
Nervous system disorders	5171	2.66 (2.58–2.74)	2.34 (4313.01)	2.34 (2.27)	1.22 (1.18)
Cardiac disorders	2412	3.82 (3.66–3.98)	3.56 (4559.09)	3.56 (3.41)	1.83 (1.77)
Psychiatric disorders	4034	3.1 (2.99–3.2)	2.78 (4848.84)	2.78 (2.68)	1.47 (1.42)
Galantamine					
Cardiac disorders	354	2.77 (2.49–3.09)	2.65 (373.56)	2.65 (2.38)	1.41 (1.24)
Lecanemab					
Nervous system disorders	2925	7.19 (6.87–7.54)	4.74 (9419.82)	4.74 (4.52)	2.24 (2.18)
Memantine					
Nervous system disorders	4608	2.88 (2.79–2.97)	2.49 (4472.24)	2.49 (2.41)	1.31 (1.27)
Psychiatric disorders	3876	3.65 (3.53–3.78)	3.19 (6152.6)	3.19 (3.08)	1.67 (1.62)

Abbreviations: SOC: system organ class; 95%CI: 95% Confidence interval; χ^2^: Chi-squared.

Counts at the SOC level represent report-event counts rather than unique case counts. A single FAERS report may contain multiple PTs and SOCs; therefore, SOC counts may exceed the total number of unique reports.

**FIGURE 2:**
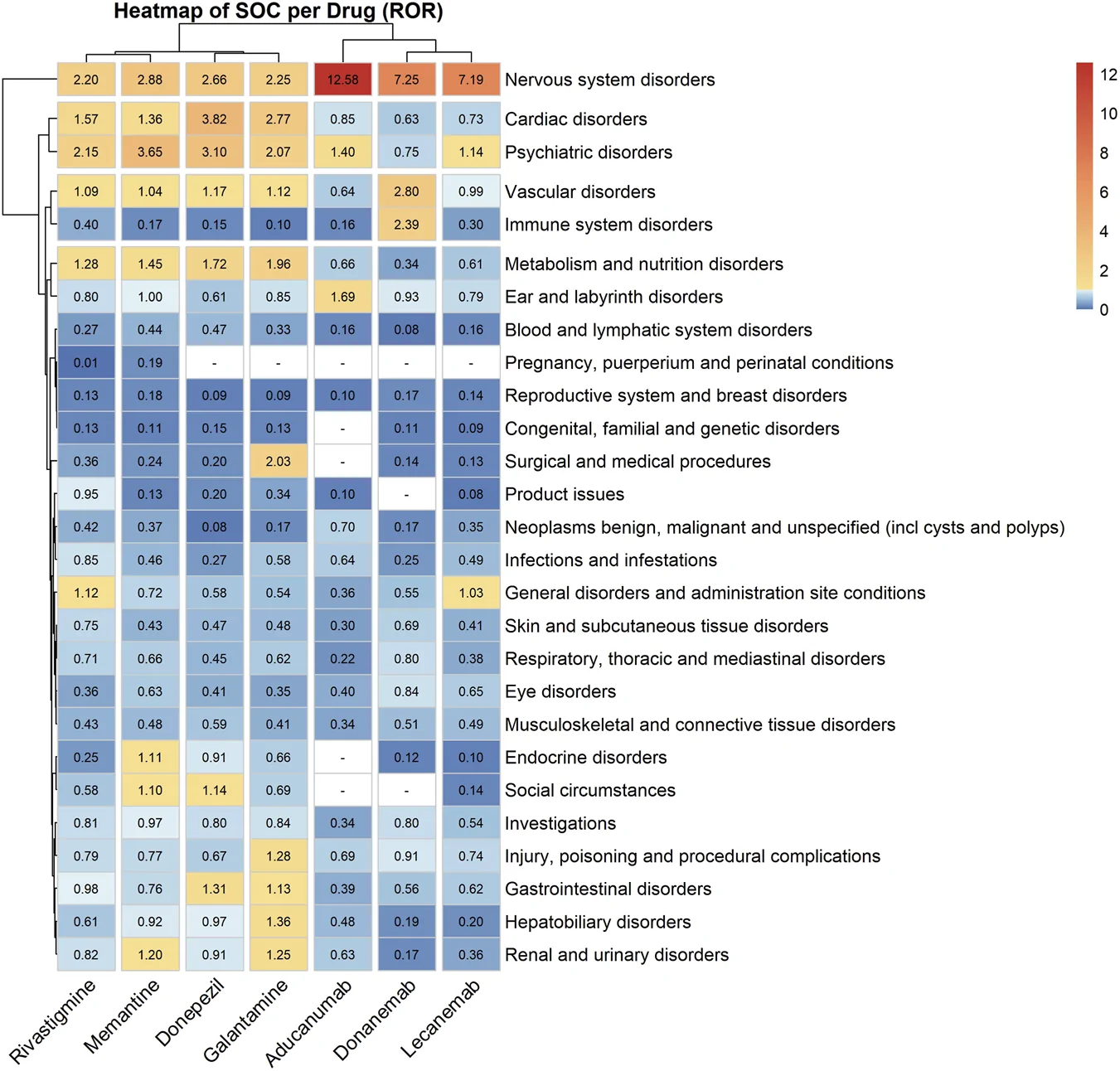
Heatmap of ROR-based signal intensity at the SOC level for seven drugs used to treat Alzheimer’s disease. Note: The heatmap was generated based on reporting odds ratio (ROR) values. An ROR of 1 was used as the neutral reference, indicating no reporting imbalance. Values > 1 suggest increased reporting, whereas values < 1 suggest decreased reporting.

Overall, adverse event signals for monoclonal antibodies were concentrated in the nervous system, whereas conventional drugs involved multiple systems, including cardiac, psychiatric, and gastrointestinal disorders.

### 3.3 Signal detection at the PT level

Figure 3 presents the overlap of PT-level positive signals identified by the four disproportionality algorithms (ROR, PRR, BCPNN, and MGPS) for each of the seven Alzheimer’s drugs. PTs located in the central intersection met the predefined positivity criteria of all four algorithms and were included in subsequent PT-level analyses. Table 4 lists the top 30 most frequently reported positive signals and their signal strengths at the PT level for each of the seven drugs following the exclusion of false-positive signals related to indication-related confounding and product packaging. The complete positive PT-level results were provided in Supplementary Tables S8–S14. PTs marked with an asterisk in Table 4 were positive reporting signals that were not listed as adverse reactions in the reviewed prescribing information for the corresponding drug. In addition, ROR values for signals positive by all four detection methods were transformed as lg (ROR + 1), and a heatmap of PT-level positive signal strength based on ROR values was generated (Figure 4; Supplementary Table S15).

**FIGURE 3:**
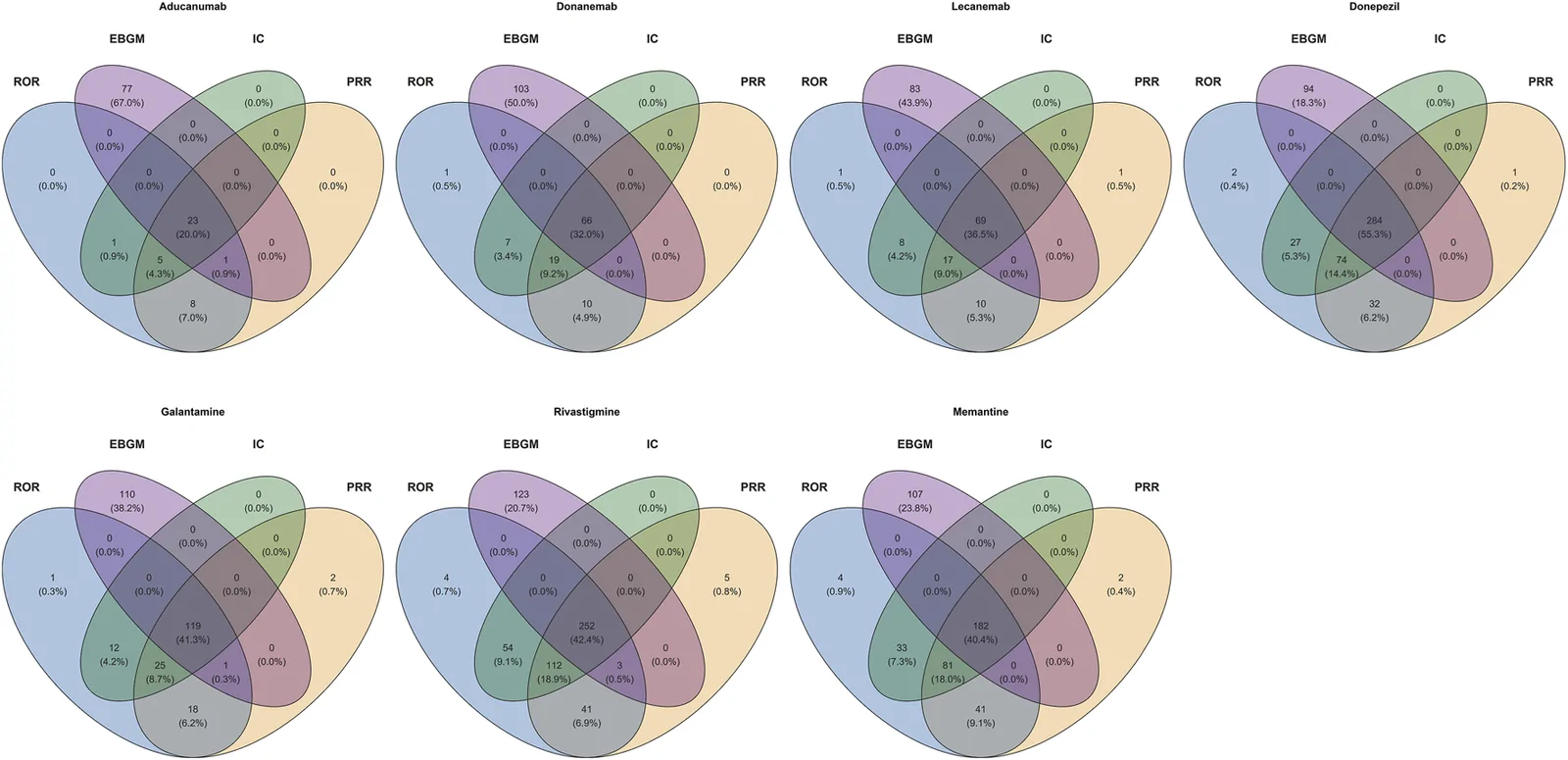
Overlap of PT-level positive signals identified by the four disproportionality algorithms (ROR, PRR, BCPNN, and MGPS) for each of the seven Alzheimer’s drugs.

**TABLE 4:** Top 30 most frequently reported adverse events at the preferred term (PT) level and signal strengths for drugs used to treat Alzheimer’s disease.

PTs	Frequency	ROR (95%Cl)	PRR (χ^2^)	EBGM(EBGM05)	IC(IC025)	FDR P value
Aducanumab						
ARIA-E	143	9731.43 (8072.48–11731.3)	8472.2 (1051765.86)	7356.74 (6102.62)	12.84 (6.87)	<0.001
ARIA-H	101	7419.77 (5981.2–9204.33)	6741.67 (607420.39)	6015.86 (4849.49)	12.55 (6.34)	<0.001
Headache	41	3.81 (2.79–5.2)	3.7 (81.66)	3.7 (2.71)	1.89 (1.34)	<0.001
Confusional state	35	12.57 (8.98–17.61)	12.21 (360.96)	12.2 (8.72)	3.61 (2.73)	<0.001
Cerebral haemorrhage	27	43.66 (29.8–63.97)	42.62 (1097.11)	42.59 (29.06)	5.41 (3.55)	<0.001
Superficial siderosis of central nervous system	23	14264.16 (8997.56–22613.48)	13967.28 (256961.56)	11174.02 (7048.36)	13.45 (3.93)	<0.001
Fall	23	3.97 (2.63–6)	3.91 (50)	3.91 (2.58)	1.97 (1.2)	<0.001
Seizure	20	6.63 (4.26–10.32)	6.53 (93.89)	6.53 (4.19)	2.71 (1.73)	<0.001
Amyloid related imaging abnormalities	17	3782.79 (2306.93–6202.82)	3724.61 (59329.31)	3491.88 (2129.52)	11.77 (3.46)	<0.001
Brain oedema	14	63.2 (37.29–107.1)	62.41 (845.17)	62.34 (36.79)	5.96 (2.86)	<0.001
*Atrial fibrillation	11	6.41 (3.54–11.61)	6.35 (49.7)	6.35 (3.51)	2.67 (1.3)	<0.001
*Subarachnoid haemorrhage	9	50.08 (25.98–96.53)	49.68 (428.96)	49.63 (25.75)	5.63 (2.16)	<0.001
*Head injury	8	14.39 (7.18–28.86)	14.3 (98.96)	14.29 (7.13)	3.84 (1.56)	<0.001
Cerebral microhaemorrhage	7	974.19 (460.39–2061.42)	968.03 (6647.08)	951.56 (449.69)	9.89 (1.96)	<0.001
Mental status changes	5	10.16 (4.22–24.45)	10.11 (41.07)	10.11 (4.2)	3.34 (0.82)	<0.001
*Transient ischaemic attack	5	8.33 (3.46–20.06)	8.3 (32.11)	8.3 (3.45)	3.05 (0.72)	<0.001
Aphasia	4	7.37 (2.76–19.66)	7.34 (21.92)	7.34 (2.75)	2.88 (0.4)	<0.001
*Posterior reversible encephalopathy syndrome	3	16.5 (5.31–51.26)	16.46 (43.56)	16.46 (5.3)	4.04 (0.31)	<0.001
*Subdural haematoma	3	11.8 (3.8–36.64)	11.77 (29.56)	11.77 (3.79)	3.56 (0.23)	<0.001
Haemorrhage intracranial	3	11.31 (3.64–35.14)	11.29 (28.12)	11.28 (3.63)	3.5 (0.21)	<0.001
*Ischaemic stroke	3	9.5 (3.06–29.51)	9.48 (22.76)	9.48 (3.05)	3.24 (0.16)	<0.001
*Skin cancer	3	7.91 (2.55–24.56)	7.89 (18.05)	7.89 (2.54)	2.98 (0.09)	0.001
*Cerebral infarction	3	6.91 (2.23–21.46)	6.89 (15.12)	6.89 (2.22)	2.79 (0.03)	0.002
Donanemab						
ARIA-E	452	7621.95 (6737.22–8622.86)	7068.68 (1864750.1)	4127.06 (3648.01)	12.01 (8.51)	<0.001
ARIA-H	369	6834.91 (5980.82–7810.97)	6429.88 (1438832.5)	3900.82 (3413.38)	11.93 (8.22)	<0.001
Infusion related reaction	342	55.2 (49.48–61.57)	52.22 (17110.37)	51.95 (46.57)	5.7 (5.34)	<0.001
Headache	259	4.29 (3.79–4.86)	4.15 (625.57)	4.15 (3.66)	2.05 (1.85)	<0.001
Flushing	189	19.16 (16.58–22.15)	18.61 (3148.94)	18.58 (16.07)	4.22 (3.87)	<0.001
Cerebral haemorrhage	162	46.74 (39.98–54.66)	45.55 (7030.97)	45.35 (38.78)	5.5 (4.93)	<0.001
Amyloid related imaging abnormalities	155	13470.47 (10581.91–17147.52)	13135.14 (875626.95)	5650.61 (4438.92)	12.46 (6.95)	<0.001
Dizziness	122	2.49 (2.08–2.98)	2.46 (106.35)	2.46 (2.05)	1.3 (1.02)	<0.001
Chills	94	8.14 (6.64–9.98)	8.04 (579.7)	8.03 (6.55)	3.01 (2.6)	<0.001
Cerebral microhaemorrhage	88	2739.99 (2161.81–3472.81)	2701.28 (186678.22)	2123.11 (1675.1)	11.05 (6.08)	<0.001
Confusional state	88	5.51 (4.47–6.8)	5.45 (320.27)	5.45 (4.41)	2.45 (2.07)	<0.001
Blood pressure increased	79	5.23 (4.19–6.53)	5.17 (266.46)	5.17 (4.14)	2.37 (1.97)	<0.001
*Back pain	74	3.22 (2.56–4.05)	3.19 (111.7)	3.19 (2.54)	1.67 (1.3)	<0.001
Chest discomfort	73	7.25 (5.76–9.14)	7.18 (388.61)	7.17 (5.7)	2.84 (2.39)	<0.001
Erythema	70	3.36 (2.66–4.25)	3.33 (114.74)	3.33 (2.63)	1.74 (1.35)	<0.001
Brain oedema	69	55.43 (43.69–70.32)	54.83 (3626.99)	54.53 (42.98)	5.77 (4.6)	<0.001
*Cerebrovascular accident	63	3.75 (2.92–4.8)	3.72 (125.61)	3.72 (2.9)	1.9 (1.47)	<0.001
Tremor	54	3.28 (2.51–4.29)	3.26 (84.73)	3.26 (2.49)	1.7 (1.25)	<0.001
Anaphylactic reaction	50	9.2 (6.96–12.15)	9.13 (362.07)	9.12 (6.91)	3.19 (2.57)	<0.001
Blood pressure decreased	43	6.53 (4.84–8.82)	6.5 (200.02)	6.49 (4.81)	2.7 (2.09)	<0.001
Feeling hot	41	6.74 (4.96–9.17)	6.7 (199.07)	6.7 (4.93)	2.74 (2.11)	<0.001
Cerebral infarction	32	13.13 (9.27–18.59)	13.07 (356.26)	13.05 (9.22)	3.71 (2.75)	<0.001
Infusion related hypersensitivity reaction	30	552.47 (382.19–798.61)	549.81 (15571.05)	520.98 (360.41)	9.03 (4.34)	<0.001
Anaphylactic shock	29	11.39 (7.91–16.4)	11.34 (273.2)	11.33 (7.86)	3.5 (2.55)	<0.001
*Oxygen saturation decreased	28	5.05 (3.48–7.32)	5.03 (90.49)	5.03 (3.47)	2.33 (1.61)	<0.001
Ischaemic stroke	23	12.96 (8.6–19.52)	12.91 (252.52)	12.9 (8.56)	3.69 (2.52)	<0.001
*Subdural haematoma	22	15.39 (10.12–23.39)	15.34 (294.45)	15.31 (10.07)	3.94 (2.64)	<0.001
*Feeling cold	19	6.85 (4.37–10.75)	6.83 (94.61)	6.83 (4.35)	2.77 (1.76)	<0.001
Head injury	18	5.72 (3.6–9.09)	5.71 (69.94)	5.71 (3.59)	2.51 (1.53)	<0.001
Aphasia	17	5.55 (3.45–8.94)	5.54 (63.25)	5.54 (3.44)	2.47 (1.46)	<0.001
Lecanemab						
ARIA-E	488	7297.37 (6460.08–8243.18)	6815.62 (1830950.35)	3753.43 (3322.77)	11.87 (8.6)	<0.001
Headache	478	6.83 (6.23–7.5)	6.46 (2224.56)	6.45 (5.88)	2.69 (2.54)	<0.001
ARIA-H	453	8310.14 (7295.56–9465.83)	7800.87 (1826734.25)	4033.98 (3541.47)	11.98 (8.5)	<0.001
Infusion related reaction	316	42.4 (37.87–47.47)	40.63 (12168.22)	40.44 (36.12)	5.34 (5)	<0.001
Chills	243	18.08 (15.91–20.55)	17.52 (3784.79)	17.49 (15.39)	4.13 (3.85)	<0.001
Fatigue	217	2.4 (2.1–2.75)	2.36 (172.34)	2.36 (2.06)	1.24 (1.03)	<0.001
Pyrexia	174	4.32 (3.72–5.03)	4.25 (433.92)	4.24 (3.65)	2.09 (1.84)	<0.001
Confusional state	166	8.84 (7.58–10.31)	8.66 (1126.77)	8.65 (7.42)	3.11 (2.82)	<0.001
Dizziness	165	2.84 (2.44–3.32)	2.8 (192.5)	2.8 (2.4)	1.49 (1.24)	<0.001
Cerebral haemorrhage	117	28.11 (23.41–33.75)	27.68 (3000.35)	27.59 (22.98)	4.79 (4.22)	<0.001
*Fall	104	2.66 (2.2–3.23)	2.64 (106.6)	2.64 (2.18)	1.4 (1.09)	<0.001
Amyloid related imaging abnormalities	92	4322.55 (3358.88–5562.72)	4268.76 (259771.63)	2825.25 (2195.39)	11.46 (6.14)	<0.001
*Tremor	91	4.67 (3.8–5.75)	4.63 (259.23)	4.62 (3.76)	2.21 (1.85)	<0.001
*Cerebral infarction	78	27.16 (21.72–33.96)	26.88 (1938.11)	26.8 (21.43)	4.74 (4.01)	<0.001
Influenza like illness	75	7.74 (6.16–9.72)	7.67 (435.23)	7.66 (6.1)	2.94 (2.48)	<0.001
Seizure	54	2.65 (2.03–3.46)	2.64 (54.95)	2.64 (2.02)	1.4 (0.96)	<0.001
Superficial siderosis of central nervous system	46	5602.81 (3855.45–8142.11)	5567.95 (153620.5)	3341.17 (2299.15)	11.71 (5.03)	<0.001
Atrial fibrillation	45	3.9 (2.91–5.23)	3.89 (96.61)	3.89 (2.9)	1.96 (1.44)	<0.001
*Feeling cold	42	12.8 (9.45–17.34)	12.74 (453.73)	12.72 (9.39)	3.67 (2.88)	<0.001
*Syncope	41	3.44 (2.53–4.68)	3.43 (70.54)	3.43 (2.52)	1.78 (1.25)	<0.001
*Balance disorder	33	3.23 (2.29–4.55)	3.22 (50.57)	3.22 (2.29)	1.69 (1.1)	<0.001
Brain oedema	30	20.09 (14.03–28.77)	20.02 (540.83)	19.97 (13.95)	4.32 (3.11)	<0.001
*Lethargy	29	4.27 (2.97–6.15)	4.26 (72.33)	4.26 (2.96)	2.09 (1.41)	<0.001
*Hallucination	29	3.28 (2.28–4.72)	3.27 (45.77)	3.27 (2.27)	1.71 (1.08)	<0.001
*Subdural haematoma	28	16.51 (11.39–23.93)	16.45 (405.57)	16.42 (11.32)	4.04 (2.88)	<0.001
Cerebral microhaemorrhage	27	589.39 (398.69–871.31)	587.24 (14763.69)	548.73 (371.19)	9.1 (4.17)	<0.001
Aphasia	23	6.33 (4.2–9.54)	6.32 (102.86)	6.31 (4.19)	2.66 (1.78)	<0.001
*Lacunar infarction	19	66.01 (42.01–103.74)	65.85 (1203.9)	65.34 (41.58)	6.03 (3.3)	<0.001
*Brain fog	19	11.06 (7.05–17.36)	11.04 (173.2)	11.02 (7.02)	3.46 (2.23)	<0.001
*Delirium	18	4.4 (2.77–6.99)	4.39 (47.18)	4.39 (2.77)	2.13 (1.23)	<0.001
Donepezil						
Bradycardia	754	34.76 (32.31–37.39)	33.8 (23674.73)	33.33 (30.98)	5.06 (4.89)	<0.001
Syncope	510	12.15 (11.13–13.26)	11.93 (5091.41)	11.88 (10.88)	3.57 (3.41)	<0.001
Vomiting	508	2.58 (2.37–2.82)	2.55 (482.92)	2.55 (2.34)	1.35 (1.22)	<0.001
*Fall	482	3.46 (3.16–3.79)	3.42 (826.43)	3.41 (3.12)	1.77 (1.63)	<0.001
Drug interaction	434	6.54 (5.95–7.2)	6.45 (1999.44)	6.44 (5.85)	2.69 (2.53)	<0.001
Confusional state	427	6.3 (5.73–6.94)	6.22 (1870.07)	6.21 (5.64)	2.63 (2.48)	<0.001
Decreased appetite	369	3.59 (3.24–3.98)	3.56 (679.39)	3.55 (3.2)	1.83 (1.67)	<0.001
Electrocardiogram qt prolonged	266	17.38 (15.4–19.62)	17.21 (4035.17)	17.1 (15.14)	4.1 (3.83)	<0.001
Agitation	258	8.31 (7.35–9.39)	8.23 (1635.95)	8.21 (7.26)	3.04 (2.82)	<0.001
Somnolence	240	2.83 (2.49–3.21)	2.81 (280.98)	2.81 (2.48)	1.49 (1.29)	<0.001
*Delirium	236	16.29 (14.33–18.53)	16.16 (3334.07)	16.05 (14.11)	4 (3.73)	<0.001
Seizure	227	3.11 (2.73–3.54)	3.09 (321.5)	3.09 (2.71)	1.63 (1.42)	<0.001
Aggression	222	10.54 (9.23–12.03)	10.46 (1891.45)	10.41 (9.12)	3.38 (3.13)	<0.001
Hallucination	220	6.98 (6.11–7.98)	6.93 (1115.08)	6.92 (6.06)	2.79 (2.56)	<0.001
Sinus bradycardia	217	54.03 (47.2–61.84)	53.59 (10949.26)	52.41 (45.78)	5.71 (5.21)	<0.001
Hypotension	202	2.38 (2.08–2.74)	2.37 (160.81)	2.37 (2.06)	1.25 (1.03)	<0.001
*Loss of consciousness	200	3.69 (3.21–4.25)	3.67 (389.36)	3.67 (3.19)	1.88 (1.65)	<0.001
Toxicity to various agents	197	2.52 (2.19–2.9)	2.51 (178.79)	2.51 (2.18)	1.33 (1.11)	<0.001
Tremor	182	2.59 (2.24–3)	2.58 (176.38)	2.58 (2.23)	1.37 (1.14)	<0.001
Nightmare	152	10.39 (8.86–12.2)	10.34 (1277.55)	10.3 (8.78)	3.36 (3.04)	<0.001
Mania	138	20.02 (16.92–23.68)	19.92 (2459.3)	19.76 (16.7)	4.3 (3.88)	<0.001
Dehydration	138	2.44 (2.06–2.88)	2.43 (116.5)	2.43 (2.06)	1.28 (1.02)	<0.001
Rhabdomyolysis	132	7.52 (6.33–8.92)	7.49 (739.87)	7.46 (6.29)	2.9 (2.58)	<0.001
*Hyperhidrosis	131	2.4 (2.02–2.85)	2.39 (106.31)	2.39 (2.01)	1.26 (0.99)	<0.001
Depressed level of consciousness	129	7.7 (6.48–9.16)	7.67 (746.53)	7.65 (6.43)	2.94 (2.61)	<0.001
*Pleurothotonus	116	345 (283.78–419.41)	343.49 (34519.41)	299.45 (246.32)	8.23 (6.11)	<0.001
Torsade de pointes	114	34.75 (28.87–41.82)	34.6 (3665.9)	34.11 (28.34)	5.09 (4.46)	<0.001
Neuroleptic malignant syndrome	113	22.68 (18.83–27.3)	22.58 (2308.92)	22.38 (18.58)	4.48 (3.96)	<0.001
Delusion	111	17.01 (14.11–20.51)	16.95 (1653.84)	16.83 (13.96)	4.07 (3.61)	<0.001
*Lethargy	109	4.48 (3.71–5.41)	4.47 (292.97)	4.46 (3.69)	2.16 (1.84)	<0.001
Galantamine						
Fall	106	3.87 (3.19–4.69)	3.81 (220.53)	3.81 (3.14)	1.93 (1.61)	<0.001
Decreased appetite	86	4.25 (3.44–5.26)	4.2 (210.48)	4.2 (3.39)	2.07 (1.71)	<0.001
Bradycardia	61	13.84 (10.75–17.82)	13.69 (717.6)	13.68 (10.63)	3.77 (3.14)	<0.001
Loss of consciousness	57	5.36 (4.12–6.95)	5.31 (199.6)	5.31 (4.09)	2.41 (1.92)	<0.001
Syncope	54	6.44 (4.93–8.42)	6.38 (245.46)	6.38 (4.88)	2.67 (2.15)	<0.001
Confusional state	52	3.86 (2.94–5.08)	3.83 (109.22)	3.83 (2.92)	1.94 (1.46)	<0.001
Somnolence	47	2.81 (2.11–3.74)	2.79 (54.26)	2.79 (2.1)	1.48 (1.01)	<0.001
Seizure	43	2.98 (2.21–4.03)	2.97 (56.26)	2.97 (2.2)	1.57 (1.07)	<0.001
Dysphagia	39	4.72 (3.44–6.47)	4.69 (113.47)	4.69 (3.42)	2.23 (1.64)	<0.001
Drug interaction	39	2.95 (2.15–4.04)	2.94 (49.89)	2.94 (2.14)	1.55 (1.03)	<0.001
Electrocardiogram qt prolonged	38	12.49 (9.07–17.18)	12.4 (398.18)	12.39 (9)	3.63 (2.8)	<0.001
Arrhythmia	38	9.6 (6.98–13.22)	9.54 (290.54)	9.53 (6.93)	3.25 (2.5)	<0.001
Agitation	37	6.01 (4.35–8.31)	5.98 (153.38)	5.97 (4.32)	2.58 (1.93)	<0.001
Delirium	36	12.51 (9.01–17.37)	12.43 (378.35)	12.42 (8.95)	3.63 (2.77)	<0.001
Renal impairment	36	5.17 (3.73–7.18)	5.14 (120.25)	5.14 (3.7)	2.36 (1.73)	<0.001
Cardiac failure	32	4.77 (3.37–6.75)	4.75 (94.75)	4.75 (3.35)	2.25 (1.59)	<0.001
Hallucination	31	4.97 (3.49–7.07)	4.95 (97.66)	4.94 (3.47)	2.31 (1.63)	<0.001
Depressed level of consciousness	30	9.08 (6.34–13)	9.03 (214.27)	9.03 (6.3)	3.17 (2.32)	<0.001
Aggression	30	7.18 (5.01–10.28)	7.14 (158.53)	7.14 (4.99)	2.84 (2.05)	<0.001
*Fracture	22	14 (9.2–21.28)	13.94 (264.04)	13.93 (9.16)	3.8 (2.55)	<0.001
*Rhabdomyolysis	22	6.34 (4.17–9.63)	6.32 (98.43)	6.31 (4.15)	2.66 (1.75)	<0.001
Hypokalaemia	20	5.2 (3.35–8.07)	5.19 (67.64)	5.19 (3.34)	2.37 (1.48)	<0.001
Altered state of consciousness	19	10.56 (6.73–16.57)	10.52 (163.65)	10.51 (6.7)	3.39 (2.19)	<0.001
*Cerebral infarction	18	8.78 (5.53–13.95)	8.75 (123.54)	8.75 (5.5)	3.13 (1.97)	<0.001
Sedation	18	8.27 (5.21–13.14)	8.24 (114.55)	8.24 (5.19)	3.04 (1.91)	<0.001
Nightmare	18	6.21 (3.91–9.86)	6.19 (78.34)	6.19 (3.9)	2.63 (1.62)	<0.001
Circulatory collapse	17	11.88 (7.38–19.14)	11.85 (168.75)	11.84 (7.35)	3.57 (2.2)	<0.001
*Cerebral haemorrhage	17	5.69 (3.53–9.16)	5.67 (65.42)	5.67 (3.52)	2.5 (1.49)	<0.001
Abnormal behaviour	17	5.09 (3.16–8.2)	5.08 (55.71)	5.08 (3.15)	2.34 (1.37)	<0.001
*Hyponatraemia	17	3.62 (2.25–5.82)	3.61 (32.07)	3.61 (2.24)	1.85 (0.97)	<0.001
Rivastigmine						
Fall	799	3.27 (3.05–3.5)	3.23 (1231.65)	3.22 (3)	1.69 (1.58)	<0.001
Vomiting	768	2.22 (2.07–2.38)	2.2 (505.11)	2.2 (2.05)	1.14 (1.03)	<0.001
Confusional state	610	5.12 (4.73–5.55)	5.07 (1987.91)	5.05 (4.66)	2.34 (2.21)	<0.001
*Cerebrovascular accident	437	3.48 (3.17–3.83)	3.46 (763.45)	3.45 (3.14)	1.79 (1.64)	<0.001
Application site erythema	433	22.65 (20.59–24.92)	22.45 (8730.27)	22.09 (20.08)	4.47 (4.26)	<0.001
Hallucination	428	7.77 (7.06–8.54)	7.7 (2485.12)	7.66 (6.97)	2.94 (2.78)	<0.001
Gait disturbance	360	2.49 (2.24–2.76)	2.48 (317.13)	2.47 (2.23)	1.31 (1.15)	<0.001
Agitation	358	6.56 (5.91–7.28)	6.52 (1665.45)	6.49 (5.85)	2.7 (2.52)	<0.001
Aggression	337	9.12 (8.19–10.15)	9.06 (2401.99)	9.01 (8.09)	3.17 (2.98)	<0.001
Application site pruritus	307	18.52 (16.54–20.73)	18.4 (4984.17)	18.16 (16.22)	4.18 (3.94)	<0.001
Tremor	304	2.47 (2.2–2.76)	2.46 (262.63)	2.45 (2.19)	1.29 (1.12)	<0.001
Urinary tract infection	303	2.4 (2.14–2.69)	2.39 (245.01)	2.39 (2.13)	1.26 (1.08)	<0.001
Syncope	301	4.02 (3.59–4.51)	4 (677.11)	3.99 (3.57)	2 (1.82)	<0.001
*Dysphagia	267	3.63 (3.22–4.09)	3.61 (504.13)	3.61 (3.2)	1.85 (1.66)	<0.001
*General physical health deterioration	266	3.11 (2.76–3.51)	3.1 (378.54)	3.1 (2.74)	1.63 (1.44)	<0.001
*Loss of consciousness	251	2.64 (2.33–2.98)	2.63 (252.87)	2.62 (2.32)	1.39 (1.2)	<0.001
*Speech disorder	246	6.35 (5.6–7.19)	6.32 (1096.71)	6.29 (5.55)	2.65 (2.44)	<0.001
Abnormal behaviour	241	8.16 (7.19–9.27)	8.13 (1497.98)	8.08 (7.12)	3.01 (2.79)	<0.001
Parkinson’s disease	227	16.1 (14.12–18.36)	16.03 (3161.32)	15.85 (13.9)	3.99 (3.7)	<0.001
Bradycardia	215	5.45 (4.77–6.24)	5.43 (774.96)	5.41 (4.73)	2.44 (2.21)	<0.001
*Gait inability	206	4.78 (4.17–5.49)	4.77 (611.64)	4.75 (4.14)	2.25 (2.02)	<0.001
*Cardiac arrest	180	2.91 (2.51–3.37)	2.9 (224.39)	2.9 (2.5)	1.54 (1.31)	<0.001
*Delirium	174	6.79 (5.85–7.89)	6.77 (852)	6.74 (5.81)	2.75 (2.49)	<0.001
*Femur fracture	157	6.83 (5.84–8)	6.81 (775.1)	6.78 (5.8)	2.76 (2.48)	<0.001
Cardiac failure	155	2.59 (2.21–3.03)	2.58 (150.46)	2.58 (2.2)	1.37 (1.12)	<0.001
Application site rash	143	14.1 (11.95–16.63)	14.06 (1716.33)	13.92 (11.8)	3.8 (3.43)	<0.001
*Cardio-respiratory arrest	133	4.24 (3.57–5.02)	4.23 (326.85)	4.22 (3.56)	2.08 (1.79)	<0.001
Restlessness	131	4.81 (4.05–5.71)	4.8 (393)	4.79 (4.03)	2.26 (1.97)	<0.001
Arrhythmia	128	3.62 (3.04–4.31)	3.61 (241.45)	3.61 (3.03)	1.85 (1.57)	<0.001
Aphasia	117	5.12 (4.27–6.15)	5.11 (385.93)	5.1 (4.25)	2.35 (2.03)	<0.001
Memantine						
Confusional state	878	15.91 (14.87–17.03)	15.32 (11720.44)	15.24 (14.25)	3.93 (3.81)	<0.001
Dizziness	731	4.24 (3.94–4.57)	4.14 (1749.66)	4.13 (3.84)	2.05 (1.93)	<0.001
Somnolence	478	6.83 (6.24–7.48)	6.7 (2320.72)	6.69 (6.11)	2.74 (2.59)	<0.001
*Fall	444	3.81 (3.47–4.19)	3.76 (902.46)	3.75 (3.42)	1.91 (1.76)	<0.001
Agitation	383	14.87 (13.44–16.46)	14.63 (4843.99)	14.56 (13.16)	3.86 (3.66)	<0.001
Asthenia	331	2.5 (2.24–2.78)	2.48 (292.64)	2.47 (2.22)	1.31 (1.14)	<0.001
Condition aggravated	258	2.42 (2.14–2.74)	2.4 (212.43)	2.4 (2.13)	1.26 (1.08)	<0.001
Constipation	236	3.09 (2.72–3.52)	3.07 (330.27)	3.07 (2.7)	1.62 (1.42)	<0.001
Aggression	231	13.13 (11.53–14.95)	13 (2549.33)	12.95 (11.37)	3.69 (3.43)	<0.001
Hallucination	226	8.59 (7.53–9.79)	8.51 (1495.22)	8.49 (7.44)	3.09 (2.85)	<0.001
Gait disturbance	209	3.03 (2.65–3.48)	3.01 (281.74)	3.01 (2.63)	1.59 (1.38)	<0.001
Seizure	208	3.41 (2.97–3.9)	3.38 (349.77)	3.38 (2.95)	1.76 (1.54)	<0.001
Decreased appetite	203	2.35 (2.04–2.7)	2.34 (155.51)	2.33 (2.03)	1.22 (1.01)	<0.001
Bradycardia	182	9.71 (8.39–11.24)	9.64 (1405.95)	9.61 (8.3)	3.26 (2.98)	<0.001
Tremor	152	2.58 (2.2–3.03)	2.57 (146.57)	2.57 (2.19)	1.36 (1.11)	<0.001
Abnormal behaviour	148	10.51 (8.94–12.36)	10.45 (1260.27)	10.41 (8.85)	3.38 (3.05)	<0.001
Syncope	139	3.89 (3.29–4.6)	3.87 (296.17)	3.87 (3.27)	1.95 (1.68)	<0.001
Loss of consciousness	135	2.97 (2.51–3.52)	2.96 (175.54)	2.96 (2.5)	1.57 (1.3)	<0.001
Lethargy	120	5.9 (4.93–7.06)	5.88 (485.03)	5.87 (4.9)	2.55 (2.23)	<0.001
Balance disorder	110	3.59 (2.98–4.33)	3.58 (204.29)	3.57 (2.96)	1.84 (1.53)	<0.001
*Pulmonary embolism	107	3.16 (2.62–3.83)	3.15 (157.4)	3.15 (2.61)	1.66 (1.35)	<0.001
*Hypersomnia	98	9.71 (7.96–11.84)	9.67 (759.27)	9.64 (7.9)	3.27 (2.86)	<0.001
*Myoclonus	97	23.13 (18.93–28.26)	23.04 (2028.28)	22.85 (18.71)	4.51 (3.93)	<0.001
Urinary incontinence	94	8.96 (7.32–10.98)	8.93 (659.91)	8.9 (7.27)	3.15 (2.74)	<0.001
*Delirium	90	7.36 (5.98–9.05)	7.33 (490.96)	7.31 (5.94)	2.87 (2.47)	<0.001
Hyponatraemia	86	4.32 (3.49–5.33)	4.3 (217.88)	4.3 (3.48)	2.1 (1.74)	<0.001
*Dyskinesia	84	5.86 (4.73–7.26)	5.84 (336.29)	5.83 (4.7)	2.54 (2.15)	<0.001
Electrocardiogram qt prolonged	77	5.94 (4.75–7.43)	5.92 (314.59)	5.91 (4.73)	2.56 (2.15)	<0.001
Depressed level of consciousness	77	5.48 (4.38–6.86)	5.46 (280.48)	5.46 (4.36)	2.45 (2.04)	<0.001
*Speech disorder	77	4.15 (3.31–5.19)	4.14 (182.91)	4.13 (3.3)	2.05 (1.66)	<0.001

Abbreviations: PT: preferred term; ARIA-E: Amyloid-related imaging abnormalities-edema/effusion; ARIA-H: Amyloid-related imaging abnormalities-hemorrhage; FDR: false discovery rate.

Counts at the PT level represent report-event counts rather than unique case counts. A single FAERS, report may contain multiple PTs; therefore, PT counts may exceed the total number of unique reports. All PTs shown in Table 4 met the predefined positivity criteria of the four disproportionality algorithms and had a Benjamini–Hochberg FDR-adjusted P value < 0.05 within the corresponding drug. PTs, marked with * were not listed as adverse reactions in the reviewed FDA-approved prescribing information.

**FIGURE 4:**
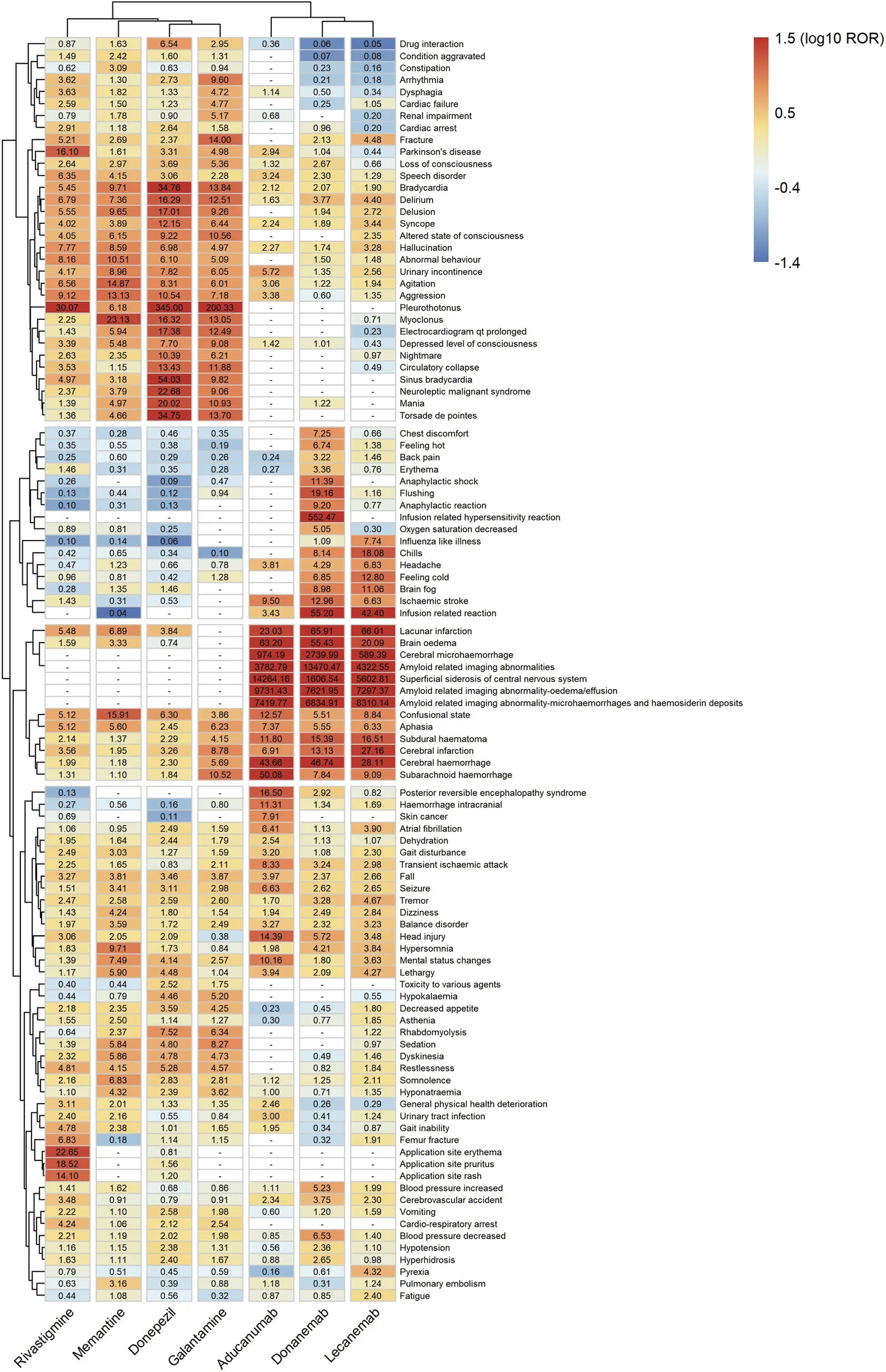
Heatmap of the top 30 positive signals at the PT level based on log-transformed ROR values for seven drugs used to treat Alzheimer’s disease.

Monoclonal antibodies showed a concentrated pattern of target-related adverse event signals. ARIA-related PTs, including Amyloid related imaging abnormalities, Amyloid-related imaging abnormalities-edema/effusion (ARIA-E) and Amyloid-related imaging abnormalities-hemorrhage (ARIA-H) showed high disproportionality. For aducanumab, ARIA-E showed ROR = 9731.43 (95% CI: 8072.48–11731.30), PRR = 8472.20 (χ^2^ = 1,051,765.86), EBGM = 7356.74 (EBGM05 = 6102.62), and IC = 12.84 (IC025 = 6.87). For donanemab, the corresponding values were ROR = 7621.95 (95% CI: 6737.22–8622.86), PRR = 7068.68 (χ^2^ = 1,864,750.10), EBGM = 4127.06 (EBGM05 = 3648.01), and IC = 12.01 (IC025 = 8.51). Donanemab and lecanemab also showed strong infusion-related reaction signals, suggesting that cerebral microvascular injury and immunogenic responses to exogenous proteins may be important safety features of this drug class.

Conventional symptomatic drugs exhibited broader peripheral cholinergic and non-specific central nervous system signals. Donepezil showed strong signals for bradycardia [ROR = 34.76 (95% CI: 32.31–37.39), PRR = 33.80 (χ^2^ = 23674.73), EBGM = 33.33 (EBGM05 = 30.98), and IC = 5.06 (IC025 = 4.89)], with syncope and falls also being prominent. Galantamine similarly showed a strong bradycardia signal [ROR = 13.84 (95% CI: 10.75–17.82), PRR = 13.69 (χ^2^ = 717.60), EBGM = 13.68 (EBGM05 = 10.63), and IC = 3.77 (IC025 = 3.14)], along with syncope. Rivastigmine showed pronounced disproportionality for application site erythema [ROR = 22.65 (95% CI: 20.59–24.92), PRR = 22.45 (χ^2^ = 8730.27), EBGM = 22.09 (EBGM05 = 20.08), and IC = 4.47 (IC025 = 4.26)]. Memantine was characterized by confusional state [ROR = 15.91 (95% CI: 14.87–17.03), PRR = 15.32 (χ^2^ = 11720.44), EBGM = 15.24 (EBGM05 = 14.25), and IC = 3.93 (IC025 = 3.81)], with dizziness and somnolence as other typical signals.

In the label-comparison analysis, several asterisk-marked PTs in Table 4 were identified as potential unlabeled reporting signals. These signals were detected by all four disproportionality algorithms and remained significant after FDR adjustment, but they were not described as adverse reactions in the reviewed prescribing information. Therefore, these findings may provide additional pharmacovigilance clues beyond known label information. However, because FAERS is a spontaneous reporting system, these asterisk-marked PTs should be interpreted as exploratory reporting signals.

### 3.4 Sex-stratified PT-level signal analysis

Because female reports accounted for a greater proportion of the total reports than male reports, PT-level ROR heatmaps were calculated separately for female and male reports to evaluate sex-stratified differences (Figures 5A,B). Sex-stratified PT-level analyses showed that the core signal spectra were broadly consistent with the overall PT-level results. Exploratory comparisons among PTs shared by female and male positive-signal sets identified selected sex-related reporting enrichments after False Discovery Rate (FDR) correction (Table 5). However, these differences were limited to a subset of PTs and did not materially alter the main drug-specific signal patterns observed in the overall analysis. For both female and male reports, ARIA-related events were the dominant signals for the three anti-Aβ monoclonal antibodies.

**FIGURE 5:**
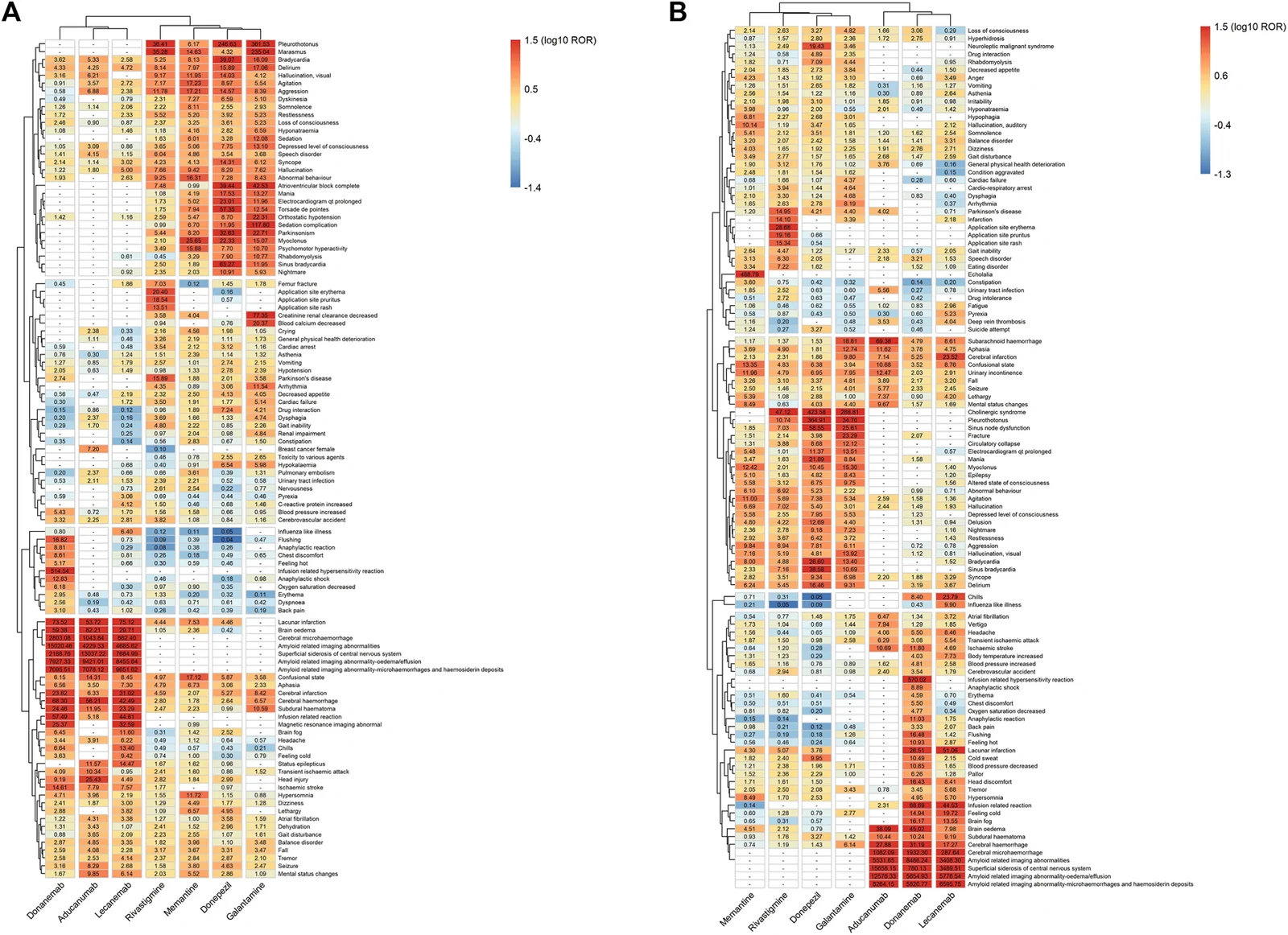
**(A)** Sex-stratified heatmap of top PT-level ROR signals for seven drugs used to treat Alzheimer’s disease in female reports. **(B)** Sex-stratified heatmap of top PT-level ROR signals for seven drugs used to treat Alzheimer’s disease in male reports.

**TABLE 5:** Exploratory sex-stratified PT-level reporting enrichment among PTs positive in both sexes.

Drug	PT	Direction	Female, n/N (%)	Male, n/N (%)	Female-to-male reporting OR (95% CI)	FDR P value
Donanemab	ARIA-E	Female-enriched	261/3254 (8.02%)	107/2086 (5.13%)	1.61 (1.28–2.03)	0.002
Lecanemab	ARIA-E	Female-enriched	304/3988 (7.62%)	133/2905 (4.58%)	1.72 (1.39–2.12)	<0.001
Lecanemab	Pyrexia	Male-enriched	63/3988 (1.58%)	95/2905 (3.27%)	0.48 (0.35–0.66)	<0.001
Lecanemab	Chills	Male-enriched	100/3988 (2.51%)	130/2905 (4.48%)	0.55 (0.42–0.72)	<0.001
Donepezil	Torsade de pointes	Female-enriched	95/13304 (0.71%)	6/9778 (0.06%)	10.87 (4.91–24.07)	<0.001
Donepezil	Electrocardiogram qt prolonged	Female-enriched	162/13304 (1.22%)	66/9778 (0.67%)	1.81 (1.36–2.41)	<0.001
Donepezil	Parkinsonism	Female-enriched	53/13304 (0.40%)	19/9778 (0.19%)	2.02 (1.20–3.40)	0.034
Donepezil	Rhabdomyolysis	Male-enriched	43/13304 (0.32%)	75/9778 (0.77%)	0.42 (0.29–0.61)	<0.001
Donepezil	Delirium	Male-enriched	89/13304 (0.67%)	119/9778 (1.22%)	0.55 (0.42–0.72)	<0.001
Donepezil	Neuroleptic malignant syndrome	Male-enriched	36/13304 (0.27%)	58/9778 (0.59%)	0.46 (0.30–0.69)	0.002
Rivastigmine	Femur fracture	Female-enriched	126/26760 (0.47%)	31/18171 (0.17%)	2.74 (1.85–4.04)	<0.001
Rivastigmine	Urinary tract infection	Female-enriched	219/26760 (0.82%)	81/18171 (0.45%)	1.84 (1.42–2.37)	<0.001
Rivastigmine	Parkinson’s disease	Male-enriched	94/26760 (0.35%)	130/18171 (0.72%)	0.49 (0.38–0.64)	<0.001
Rivastigmine	Abnormal behaviour	Male-enriched	117/26760 (0.44%)	120/18171 (0.66%)	0.66 (0.51–0.85)	0.026
Rivastigmine	Bradycardia	Male-enriched	95/26760 (0.36%)	100/18171 (0.55%)	0.64 (0.49–0.85)	0.033
Memantine	Mental status changes	Male-enriched	24/11838 (0.20%)	44/8561 (0.51%)	0.40 (0.24–0.65)	0.004
Memantine	Bradycardia	Male-enriched	65/11838 (0.55%)	77/8561 (0.90%)	0.61 (0.44–0.85)	0.045

Abbreviations: OR: odds ratio.

n/N (%) indicates target PT reports divided by target PT plus other PT reports within the corresponding drug-sex stratum. Female-to-male reporting OR > 1 indicates female-enriched reporting; OR < 1 indicates male-enriched reporting. Only PTs detected as positive signals in both female and male reports and meeting FDR-adjusted P < 0.05 with an effect-size cutoff (female-to-male reporting OR ≥ 1.5 or ≤ 0.67) are shown. Values represent reporting enrichment in spontaneous reports and should not be interpreted as true incidence differences.

Of the conventional symptomatic drugs, reports from males exhibited more prominent signals related to cholinergic syndrome and conduction abnormalities. For example, the ROR values for cholinergic syndrome were 423.58 for galantamine and 288.81 for donepezil. When considering reports from females, arrhythmia-related signals for donepezil were more prominent, including sinus bradycardia, torsade de pointes, and bradycardia (ROR = 65.27, 57.35, and 39.07, respectively). For both males and females, rivastigmine was characterized predominantly by reactions at the application site, whereas memantine was characterized predominantly by consciousness, behavioral, and neuropsychiatric symptoms.

### 3.5 Age-stratified PT-level analysis

Age-stratified analyses were performed using the ten most frequently reported PTs identified in the primary analysis for each drug (Figure 6). Overall, the PT spectra remained largely consistent across adult age groups, with most differences limited to changes in signal ranking rather than the emergence of age-specific reporting patterns. Among the three anti-Aβ monoclonal antibodies, ARIA-related events, cerebral haemorrhage, headache, and infusion-related reactions remained the dominant reporting signals across adult age groups, with the 65–85 years subgroup showing the most complete and stable signal pattern. Likewise, the conventional symptomatic drugs exhibited broadly preserved PT spectra across age groups. Donepezil was characterized primarily by cardiovascular and neuropsychiatric PTs, whereas memantine remained dominated by confusional state, agitation, dizziness, and somnolence. Only modest variations in signal ranking were observed for rivastigmine and memantine, while donepezil and galantamine showed nearly identical PT profiles across adult age groups. Interpretation of the <18 years subgroup should be made with caution because of the limited number of reports and substantial missing age information.

**FIGURE 6:**
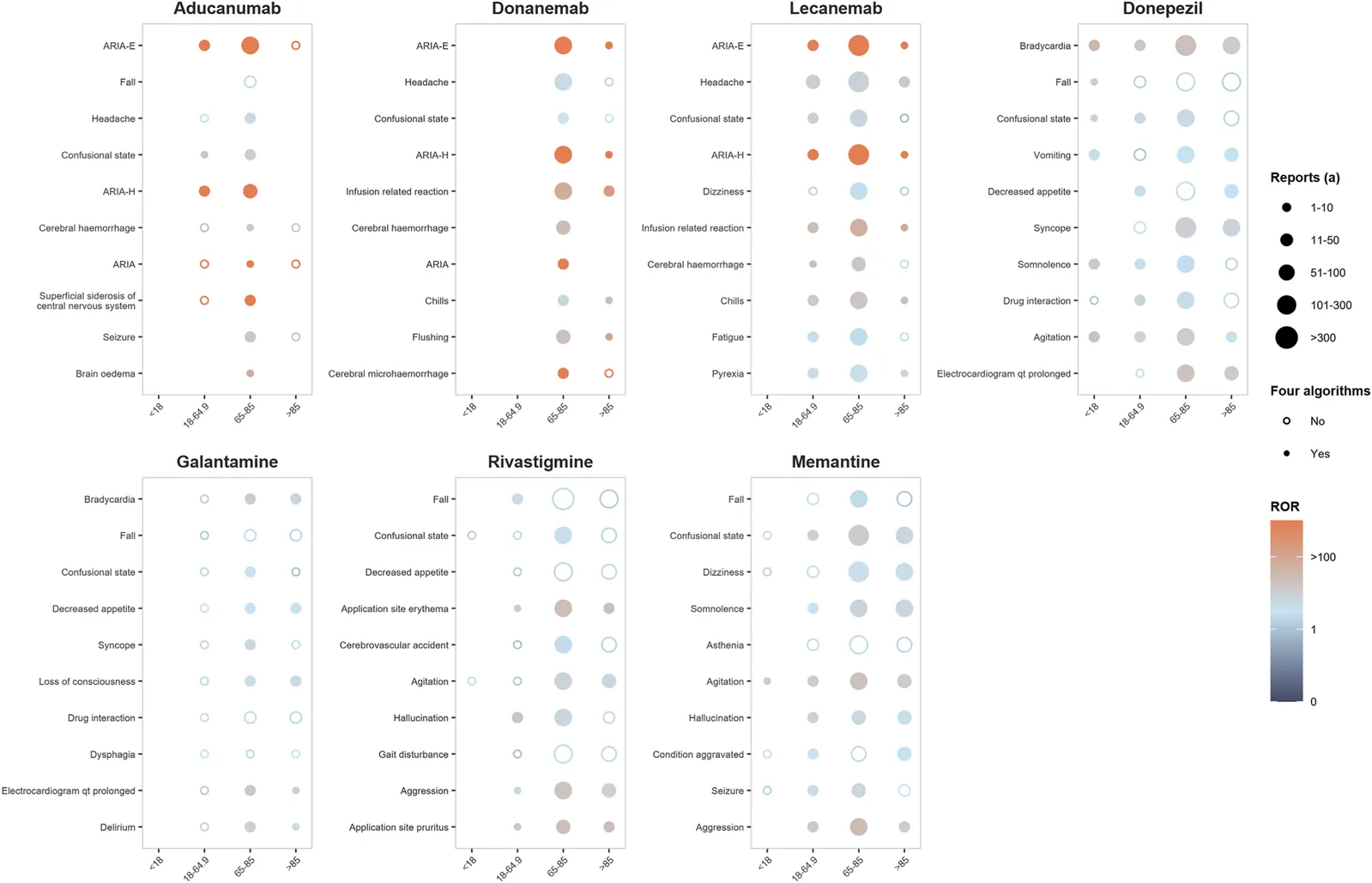
Age-stratified heatmap of PT-level reporting signals for the top 10 preferred terms of each anti-Alzheimer’s disease drug.

### 3.6 TTO analysis

Analysis revealed differences in TTO between the seven anti-AD drugs (Table 6; Figure 7).

**TABLE 6:** Median time-to-onset (TTO) of adverse events for drugs used to treat Alzheimer’s disease (Days).

Drug	Valid TTO reports, n	Overall median time-to-onset (IQR)	Median time-to-onset for females (IQR)	Median time-to-onset for males (IQR)	Weibull β (95% CI); onset pattern
Lecanemab	1089	50 (15.0,168)	52.0 (21.0,154.0)	48.0 (14.0,174.5)	0.677 (0.646–0.708); early-failure
Aducanumab	208	147.5 (89.0,210.0)	150.5 (88.0,206.3)	147.0 (95.0,209.0)	1.286 (1.158–1.414); wear-out/delayed
Donanemab	420	36.0 (26.0,85.0)	35.0 (25.0,84.0)	35.5 (28.0,90.25)	1.042 (0.996–1.118); random
Donepezil	2563	45.0 (10.0,281.0)	45.5 (12.0,279.0)	49.5 (8.0,302.5)	0.528 (0.513–0.544); early-failure
Galantamine	319	41.0 (12.0,190.0)	37.0 (12.0,201.0)	42.5 (12.8,189.0)	0.537 (0.493–0.581); early-failure
Rivastigmine	2282	76.0 (18.0,269.75)	70.0 (16.0,246.0)	83.0 (21.5,311.5)	0.576 (0.558–0.594); early-failure
Memantine	2163	18.0 (4.0,91.0)	17.0 (4.0,63.25)	17.0 (4.0,95.0)	0.500 (0.484–0.515); early-failure

Abbreviations: IQR: interquartile range; β: Weibull shape parameter.

TTO was summarized as median and interquartile range. Records with missing, non-numeric, or negative TTO values were considered invalid and excluded from TTO analyses without imputation. β < 1 indicates an early-failure pattern, β ≈ 1 indicates a random pattern, and β > 1 indicates a wear-out or delayed-onset pattern.

**FIGURE 7:**
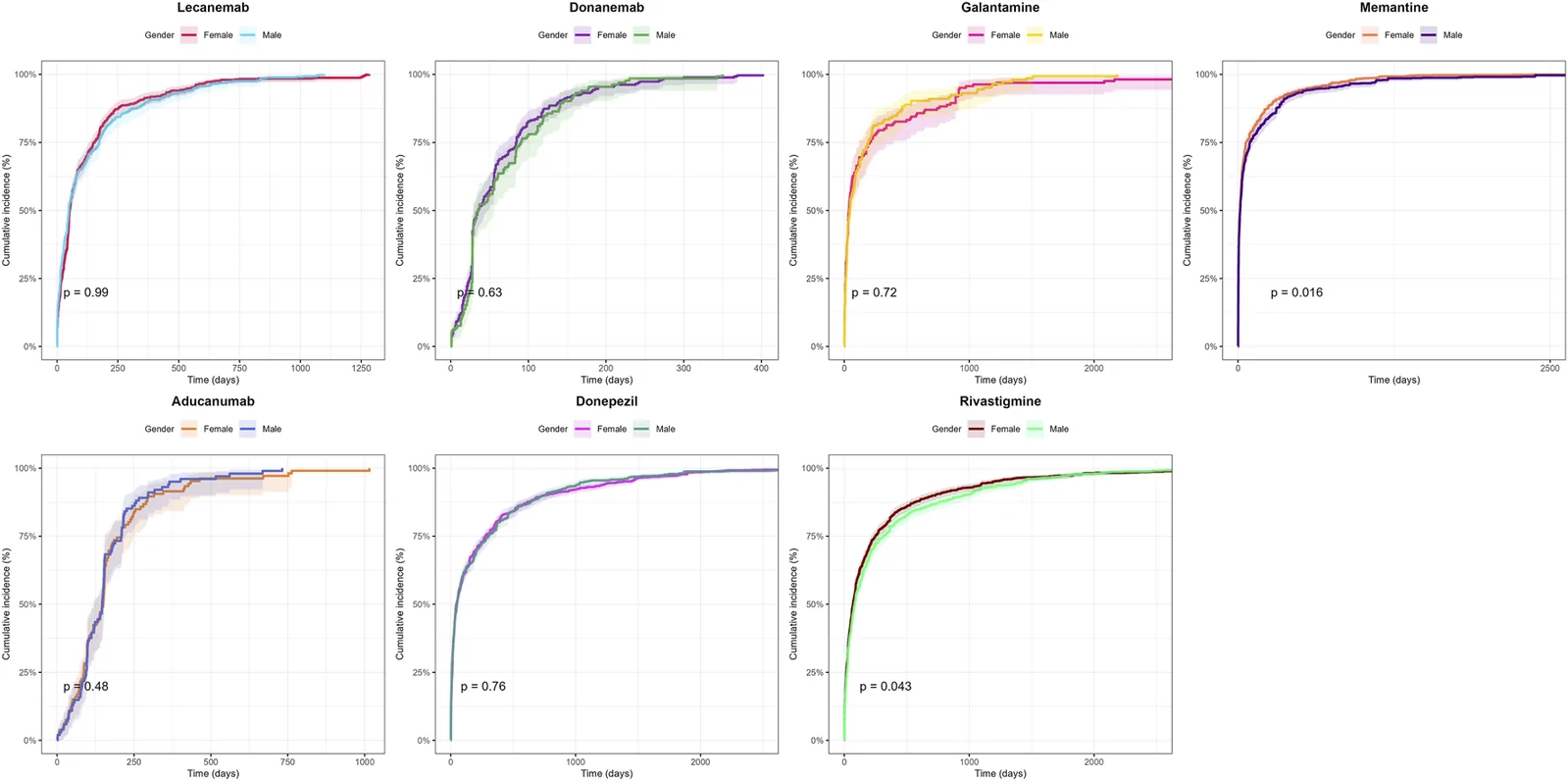
Sex-stratified cumulative distribution curves of time to onset across different treatments for Alzheimer’s disease.

Overall, memantine-associated adverse events occurred earliest, with a median TTO of 18 days. The median TTOs for donanemab, galantamine, donepezil, and lecanemab were observed during the early to mid-treatment period (36, 41, 45, and 50 days, respectively). The median TTO for rivastigmine was 76 days, while that of aducanumab was 147.5 days. TTO distributions differed significantly across the seven drugs according to the Kruskal–Wallis rank-sum test (H = 480.22, df = 6, P < 0.001; Supplementary Table S16). Pairwise Wilcoxon rank-sum tests with Benjamini–Hochberg correction were further performed to compare TTO distributions between individual drugs, and the complete pairwise results are provided in Supplementary Table S17. Weibull analysis showed that most drugs had β values below 1, indicating early-failure onset patterns. Aducanumab showed a delayed or wear-out pattern, whereas donanemab showed an approximately random pattern because the 95% CI of β included 1.

To further characterize onset patterns, PT-level TTO analyses were performed for the five most frequently reported PTs of each drug (Supplementary Table S18). Overall, variation in onset timing was observed among PTs within the same drug. For anti-Aβ monoclonal antibodies, infusion-related reactions and constitutional symptoms generally occurred earlier than ARIA-related events. In contrast, conventional symptomatic drugs tended to show earlier onset of gastrointestinal and neuropsychiatric events, whereas cardiovascular and fall-related events occurred relatively later. PT-level Weibull analyses were broadly consistent with the drug-level findings, with most PTs exhibiting early-failure patterns (β < 1).

Following sex stratification, differences in TTO between female and male reports were not significant for most drugs. Statistically significant differences were only observed for memantine (both 17 days in female and male reports, P = 0.016) and rivastigmine (70 days in female reports vs. 83 days in male reports, P = 0.043). For memantine, the median TTO has no differences in female and male, suggesting limited clinical relevance despite statistical significance. For rivastigmine, TTO was approximately 13 days shorter in female reports than in male reports, suggesting a potential difference in the timing of treatment intolerance; however, this finding should be interpreted cautiously because TTO data were available only for a subset of reports.

## 4 Discussion

This study used data from the FAERS database to perform signal detection for seven anti-AD drugs: aducanumab, donanemab, lecanemab, donepezil, galantamine, rivastigmine, and memantine. A total of 39,301 valid adverse event reports were included in the final analysis; our goal was to provide real-world evidence for the safety evaluation of pharmacotherapy for AD and to inform the rational selection of drugs, risk identification, and safety management for anti-dementia treatment (Cummings et al., 2021; Cummings et al., 2023; Cummings et al., 2022; Rabinovici et al., 2025).

Overall, women accounted for 52.3% of all reports, and patients aged 65 years or older constituted the predominant age population. Reports for anti-Aβ monoclonal antibodies were more concentrated among patients aged 65–85 years, whereas conventional symptomatic drugs were noted in a relatively higher proportion of reports among patients aged > 85 years. This distribution is broadly consistent with current real-world application in that monoclonal antibodies are used more often in earlier stages of disease and in more strictly selected populations, whereas AChEIs and memantine are used in broader and older dementia populations (Budd et al., 2022; Honig et al., 2024; McDade et al., 2022; Mintun et al., 2021).

At both the SOC and PT levels, the adverse event profiles of the seven anti-AD drugs showed clear class-specific differences. Anti-Aβ monoclonal antibodies were characterized by concentrated signals involving the nervous system, whereas conventional symptomatic drugs involved multiple systems, including cardiac, neuropsychiatric, gastrointestinal, and administration-site disorders. These differences were closely related to drug targets and the treated populations. Anti-Aβ monoclonal antibodies act directly on cerebral Aβ pathology, and adverse events associated with these drugs are therefore more likely to involve the central nervous system and cerebral microvascular changes. In contrast, AChEIs and memantine predominantly improve symptoms by modulating neurotransmitter systems, which may lead to peripheral cholinergic reactions, cardiovascular conduction abnormalities, and fluctuations in neuropsychiatric status (Xing et al., 2025; Yang et al., 2024).

ARIA-related events were the most important safety signals for the three anti-Aβ monoclonal antibodies. ARIA-E, ARIA-H, cerebral microhemorrhage, and superficial siderosis were highly prominent for aducanumab, donanemab, and lecanemab, indicating that cerebral microvascular injury-related events were the predominant reporting signals for this class of drug. This finding is consistent with a mechanism in which the removal of perivascular amyloid deposition may induce changes in vascular permeability, cerebral edema, and microhemorrhage. Donanemab and lecanemab were also associated with strong infusion-related reaction signals, indicating that the clinical risk of anti-Aβ monoclonal antibodies is not limited to imaging abnormalities but also includes systemic immune reactions during early administration (Sims et al., 2023; van Dyck et al., 2023; Salloway et al., 2022). Therefore, safety management for anti-Aβ monoclonal antibodies should emphasize baseline Magnetic resonance imaging (MRI) assessment, serial MRI follow-up during early treatment cycles, patient education regarding ARIA warning symptoms (such as headache, confusion, visual disturbance, gait change, or seizures), and close observation for infusion-related reactions, particularly after initial administrations. The exceptionally strong and concordant ARIA-related signals, together with their biological plausibility, reinforce ARIA as the defining safety concern of anti-Aβ monoclonal antibodies.

The signals observed for donepezil and galantamine reflected the systemic effects of enhanced cholinergic activity induced by AChEIs. Bradycardia, syncope, and falls may not be isolated events; instead, they may reflect a clinically relevant cluster of reporting signals in which reduced heart rate or the suppression of conduction causes syncope and subsequently fall-related injury (Ruangritchankul et al., 2021; Young et al., 2021; Ahuja et al., 2023). These risks are clinically relevant for patients with AD because these patients are often older, have cardiovascular comorbidities or orthostatic hypotension, and receive multiple types of medication. Falls may lead to fractures, hospitalization, and functional decline. Therefore, monitoring during AChEI therapy should not be limited to gastrointestinal reactions but should also include baseline heart rate, electrocardiographic findings, conduction function, the history of syncope and falls, and a structured review of concomitant rate-limiting drugs (such as beta-blockers, non-dihydropyridine calcium-channel blockers, and digoxin). These measures may be particularly important in older adults, patients with low body weight, and those with pre-existing cardiovascular disease.

Rivastigmine and memantine represented two additional monitoring priorities. The most characteristic signal for rivastigmine was reactions at the site of administration, suggesting that although transdermal patches can improve gastrointestinal tolerability in some patients, local skin irritation remains an important issue in clinical application (Fusaroli et al., 2024; For et al., 2025; García and Ferrer-Picón, 2026). Furthermore, higher reporting burdens for pneumonia, falls, and death should be interpreted cautiously in the context of longer disease duration, a poorer functional status, dysphagia, and a higher risk of infection, rather than being attributed directly to the drug. Memantine-related adverse events were more often reflected as fluctuations in consciousness and behavior, including confusional state, agitation, dizziness, somnolence, and falls. This pattern is consistent with the potential effects of NMDA receptor modulation on the central excitatory-inhibitory balance. Clinically, for rivastigmine patch therapy, routine rotation of application sites, early recognition of erythema or pruritus, and assessment of frailty-related risks such as aspiration, infection, and falls are advisable. For memantine, changes in consciousness, dizziness, somnolence, neuropsychiatric symptoms, and fall tendency should be monitored particularly during treatment initiation and dose escalation, and dose adjustment should be considered in patients with renal impairment (Yang et al., 2026).

The asterisk-marked PTs in Table 4 provide an additional label-comparison perspective for interpreting the PT-level signal spectrum. These signals were positive in disproportionality analyses but were not listed as adverse reactions in the reviewed prescribing information for the corresponding drugs. Therefore, they may represent potential unlabeled reporting signals that deserve further pharmacovigilance attention. However, such findings should not be interpreted as newly confirmed adverse reactions, because FAERS data cannot determine incidence, causality, or the influence of disease progression, comorbidities, and concomitant medications. Similarly, low-frequency signals should be interpreted cautiously because disproportionality estimates may be unstable and should be regarded as signals requiring further validation rather than confirmed high-risk adverse events. Clinically, these signals should be regarded as hypothesis-generating findings and may help guide targeted monitoring in future observational studies or clinical practice.

Subgroup analyses further indicated the existence of sex-related differences although this did not constitute a major basis for risk stratification overall (Yang et al., 2024; Castro-Aldrete et al., 2023; Lim et al., 2025). Similarly, age subgroup analysis indicated that the principal PT spectrum of each drug remained largely consistent across age groups. Although modest rank shifts were observed, particularly for aducanumab and donanemab, no age-specific dominant reporting signals emerged. These findings suggest that age may influence the relative prominence of established adverse events rather than fundamentally altering the overall safety profile. In terms of onset time, only memantine and rivastigmine showed statistically significant differences between sex. The median TTO for memantine was both 17 days in female male reports, thus indicating limited clinical relevance. The median TTO for rivastigmine was shorter in female reports than in male reports (70 vs. 83 days, P = 0.043), thus suggesting a potentially earlier reporting onset in women, but this should be interpreted cautiously because of the inherent limitations of spontaneous reporting data. PT heatmaps also revealed that the core signal spectra were generally consistent when compared between female and male reports. ARIA-related events dominated for anti-Aβ monoclonal antibodies in both sexes, thus suggesting that risk is primarily driven by drug mechanism and cerebral microvascular pathology rather than by sex itself. Some sex-specific differences observed for conventional symptomatic drugs, such as stronger cholinergic syndrome signals in male reports and more prominent donepezil-related arrhythmia signals in female reports, may be associated with differences in body weight, frailty, baseline cardiovascular status, renal function, electrolyte status, and concomitant medications. However, these findings should be viewed as reporting signals rather than incidence-based risk estimates (Kong et al., 2025). For clinical practice, closer monitoring of pulse rate and conduction abnormalities may be warranted in patients receiving AChEIs, especially when additional rate-limiting drugs are co-prescribed; women receiving rivastigmine may benefit from earlier follow-up after initiation. For memantine, careful observation of consciousness, behavioral status, dizziness, and fall risk during the early treatment period remains advisable. PT-level TTO analyses further indicated that onset timing varied across different adverse events within the same drug. Early-occurring infusion-related or gastrointestinal events may require closer monitoring shortly after treatment initiation, whereas delayed ARIA-related and cardiovascular events support continued safety surveillance during follow-up. Collectively, these findings provide not only statistical evidence for signal detection but also clinically relevant information regarding drug-specific risk profiles, subgroup consistency, and temporal characteristics that may facilitate individualized pharmacovigilance and safety monitoring.

This study has certain limitations that should be considered, particularly because this study was based on a spontaneous reporting pharmacovigilance system. First, the FAERS database can allow for the identification of statistical associations between drugs and adverse events but cannot establish causality. Second, under-reporting, duplicate reports, stimulated reporting, notoriety effects after the market entry of new drugs, comorbidities, and polypharmacy can all influence the interpretation of signal strength. Although indication-related confounding and false-positive signals related to product packaging were removed as far as possible and four algorithms (ROR, PRR, BCPNN, and MGPS) were used for cross-validation to improve robustness, outcomes such as falls, confusional state, and pneumonia should still be interpreted cautiously (Cutroneo et al., 2023; Fusaroli et al., 2024; Potter et al., 2025). Similarly, asterisk-marked signals in Table 4 should be interpreted as signals requiring further validation rather than evidence of newly established adverse reactions. In addition, PT-level multiplicity was addressed using the Benjamini–Hochberg FDR procedure, but residual confounding and reporting bias cannot be eliminated in spontaneous reporting systems.

## 5 Conclusion

Using the FAERS database, we systematically compared adverse event signals for seven anti-AD drugs by applying four disproportionality analysis methods. Our findings indicated heterogeneity in safety profiles between drugs. Risks associated with anti-Aβ monoclonal antibodies were predominantly concentrated in the nervous system, with ARIA-related events and infusion-related reactions as core signals. Conventional symptomatic drugs were associated with broader multi-system safety signals. In addition, identified several potential unlabeled reporting signals. Collectively, our findings provide real-world reporting evidence to support individualized and risk-differentiated safety assessment and clinical use of anti-AD drugs, while acknowledging the exploratory nature and inherent limitations of spontaneous reporting data.

## Data Availability

The original contributions presented in the study are included in the article/Supplementary Material, further inquiries can be directed to the corresponding author.
