# Supplementary material for: Disproportionality analysis of adverse event signals associated with seven Alzheimer’s drugs: insights from the FAERS database
Source: Front Pharmacol. 2026 Sep 24;17:1955230. doi: 10.3389/fphar.2026.1955230 (PMC13646139; doi:10.3389/fphar.2026.1955230)
Supplement: Supplementary file 1 [file Table1.docx]

Supplementary Material

Supplementary Table S1. Distribution of adverse events and signal detection results at the SOC level for Aducanumab.

| SOC | a | b | c | d | ROR(95%CI) | PRR(X^2^) | EBGM(EBGM05) | IC(IC025) |
| --- | --- | --- | --- | --- | --- | --- | --- | --- |
| Eye disorders | 9 | 1096 | 1253514 | 60481846 | 0.4 ( 0.21 - 0.76 ) | 0.4 ( 8.21 ) | 0.4 ( 0.21 ) | -1.32 ( -2.15 ) |
| Investigations | 24 | 1081 | 3741908 | 57993452 | 0.34 ( 0.23 - 0.52 ) | 0.36 ( 29.36 ) | 0.36 ( 0.24 ) | -1.48 ( -2.03 ) |
| Product issues | 2 | 1103 | 1058962 | 60676398 | 0.1 ( 0.03 - 0.42 ) | 0.11 ( 15.43 ) | 0.11 ( 0.03 ) | -3.24 ( -4.4 ) |
| Vascular disorders | 15 | 1090 | 1295833 | 60439527 | 0.64 ( 0.39 - 1.07 ) | 0.65 ( 2.96 ) | 0.65 ( 0.39 ) | -0.63 ( -1.32 ) |
| Hepatobiliary disorders | 5 | 1100 | 574988 | 61160372 | 0.48 ( 0.2 - 1.16 ) | 0.49 ( 2.75 ) | 0.49 ( 0.2 ) | -1.04 ( -2.09 ) |
| Nervous system disorders | 590 | 515 | 5153607 | 56581753 | 12.58 ( 11.18 - 14.16 ) | 6.4 ( 2930.25 ) | 6.4 ( 5.68 ) | 2.68 ( 2.52 ) |
| Immune system disorders | 2 | 1103 | 689717 | 61045643 | 0.16 ( 0.04 - 0.64 ) | 0.16 ( 8.77 ) | 0.16 ( 0.04 ) | -2.63 ( -3.82 ) |
| Cardiac disorders | 24 | 1081 | 1577478 | 60157882 | 0.85 ( 0.56 - 1.27 ) | 0.85 ( 0.65 ) | 0.85 ( 0.57 ) | -0.23 ( -0.81 ) |
| Psychiatric disorders | 83 | 1022 | 3384418 | 58350942 | 1.4 ( 1.12 - 1.75 ) | 1.37 ( 8.78 ) | 1.37 ( 1.1 ) | 0.45 ( 0.12 ) |
| Musculoskeletal and connective tissue disorders | 20 | 1085 | 3178549 | 58556811 | 0.34 ( 0.22 - 0.53 ) | 0.35 ( 25.22 ) | 0.35 ( 0.23 ) | -1.51 ( -2.1 ) |
| Neoplasms benign, malignant and unspecified (incl cysts and polyps) | 20 | 1085 | 1579091 | 60156269 | 0.7 ( 0.45 - 1.09 ) | 0.71 ( 2.48 ) | 0.71 ( 0.45 ) | -0.5 ( -1.12 ) |
| Metabolism and nutrition disorders | 16 | 1089 | 1335967 | 60399393 | 0.66 ( 0.41 - 1.09 ) | 0.67 ( 2.68 ) | 0.67 ( 0.41 ) | -0.58 ( -1.26 ) |
| Reproductive system and breast disorders | 1 | 1104 | 535468 | 61199892 | 0.1 ( 0.01 - 0.74 ) | 0.1 ( 7.76 ) | 0.1 ( 0.01 ) | -3.26 ( -4.45 ) |
| General disorders and administration site conditions | 77 | 1028 | 10742236 | 50993124 | 0.36 ( 0.28 - 0.45 ) | 0.4 ( 83.67 ) | 0.4 ( 0.32 ) | -1.32 ( -1.65 ) |
| Injury, poisoning and procedural complications | 85 | 1020 | 6616986 | 55118374 | 0.69 ( 0.56 - 0.87 ) | 0.72 ( 10.57 ) | 0.72 ( 0.58 ) | -0.48 ( -0.8 ) |
| Blood and lymphatic system disorders | 3 | 1102 | 1046486 | 60688874 | 0.16 ( 0.05 - 0.49 ) | 0.16 ( 13.44 ) | 0.16 ( 0.05 ) | -2.64 ( -3.75 ) |
| Infections and infestations | 38 | 1067 | 3254337 | 58481023 | 0.64 ( 0.46 - 0.88 ) | 0.65 ( 7.43 ) | 0.65 ( 0.47 ) | -0.62 ( -1.07 ) |
| Gastrointestinal disorders | 39 | 1066 | 5260437 | 56474923 | 0.39 ( 0.29 - 0.54 ) | 0.41 ( 35.32 ) | 0.41 ( 0.3 ) | -1.27 ( -1.72 ) |
| Ear and labyrinth disorders | 8 | 1097 | 265061 | 61470299 | 1.69 ( 0.84 - 3.39 ) | 1.69 ( 2.24 ) | 1.69 ( 0.84 ) | 0.75 ( -0.32 ) |
| Renal and urinary disorders | 13 | 1092 | 1149214 | 60586146 | 0.63 ( 0.36 - 1.08 ) | 0.63 ( 2.84 ) | 0.63 ( 0.37 ) | -0.66 ( -1.4 ) |
| Skin and subcutaneous tissue disorders | 19 | 1086 | 3388587 | 58346773 | 0.3 ( 0.19 - 0.47 ) | 0.31 ( 30.27 ) | 0.31 ( 0.2 ) | -1.67 ( -2.28 ) |
| Respiratory, thoracic and mediastinal disorders | 12 | 1093 | 2900319 | 58835041 | 0.22 ( 0.13 - 0.39 ) | 0.23 ( 32.2 ) | 0.23 ( 0.13 ) | -2.11 ( -2.83 ) |

Supplementary Table S2. Distribution of adverse events and signal detection results at SOC level for Donanemab.

| SOC | a | b | c | d | ROR(95%CI) | PRR(X^2^) | EBGM(EBGM05) | IC(IC025) |
| --- | --- | --- | --- | --- | --- | --- | --- | --- |
| Eye disorders | 106 | 6120 | 1253417 | 60476822 | 0.84 ( 0.69 - 1.01 ) | 0.84 ( 3.37 ) | 0.84 ( 0.69 ) | -0.25 ( -0.53 ) |
| Investigations | 307 | 5919 | 3741625 | 57988614 | 0.8 ( 0.72 - 0.9 ) | 0.81 ( 13.97 ) | 0.81 ( 0.73 ) | -0.3 ( -0.47 ) |
| Endocrine disorders | 2 | 6224 | 160950 | 61569289 | 0.12 ( 0.03 - 0.49 ) | 0.12 ( 12.51 ) | 0.12 ( 0.03 ) | -3.02 ( -4.19 ) |
| Vascular disorders | 352 | 5874 | 1295496 | 60434743 | 2.8 ( 2.51 - 3.11 ) | 2.69 ( 382.88 ) | 2.69 ( 2.42 ) | 1.43 ( 1.26 ) |
| Hepatobiliary disorders | 11 | 6215 | 574982 | 61155257 | 0.19 ( 0.1 - 0.34 ) | 0.19 ( 38.44 ) | 0.19 ( 0.1 ) | -2.4 ( -3.13 ) |
| Nervous system disorders | 2476 | 3750 | 5151721 | 56578518 | 7.25 ( 6.89 - 7.63 ) | 4.77 ( 8033.55 ) | 4.76 ( 4.53 ) | 2.25 ( 2.18 ) |
| Immune system disorders | 164 | 6062 | 689555 | 61040684 | 2.39 ( 2.05 - 2.8 ) | 2.36 ( 129.7 ) | 2.36 ( 2.02 ) | 1.24 ( 1 ) |
| Cardiac disorders | 101 | 6125 | 1577401 | 60152838 | 0.63 ( 0.52 - 0.77 ) | 0.63 ( 21.77 ) | 0.63 ( 0.52 ) | -0.66 ( -0.94 ) |
| Psychiatric disorders | 259 | 5967 | 3384242 | 58345997 | 0.75 ( 0.66 - 0.85 ) | 0.76 ( 21.01 ) | 0.76 ( 0.67 ) | -0.4 ( -0.58 ) |
| Musculoskeletal and connective tissue disorders | 169 | 6057 | 3178400 | 58551839 | 0.51 ( 0.44 - 0.6 ) | 0.53 ( 75.55 ) | 0.53 ( 0.45 ) | -0.92 ( -1.14 ) |
| Congenital, familial and genetic disorders | 2 | 6224 | 181201 | 61549038 | 0.11 ( 0.03 - 0.44 ) | 0.11 ( 14.54 ) | 0.11 ( 0.03 ) | -3.19 ( -4.35 ) |
| Neoplasms benign, malignant and unspecified (incl cysts and polyps) | 27 | 6199 | 1579084 | 60151155 | 0.17 ( 0.11 - 0.24 ) | 0.17 ( 112.72 ) | 0.17 ( 0.12 ) | -2.56 ( -3.06 ) |
| Metabolism and nutrition disorders | 47 | 6179 | 1335936 | 60394303 | 0.34 ( 0.26 - 0.46 ) | 0.35 ( 58.4 ) | 0.35 ( 0.26 ) | -1.52 ( -1.92 ) |
| Reproductive system and breast disorders | 9 | 6217 | 535460 | 61194779 | 0.17 ( 0.09 - 0.32 ) | 0.17 ( 37.83 ) | 0.17 ( 0.09 ) | -2.58 ( -3.37 ) |
| Surgical and medical procedures | 12 | 6214 | 860404 | 60869835 | 0.14 ( 0.08 - 0.24 ) | 0.14 ( 65.35 ) | 0.14 ( 0.08 ) | -2.85 ( -3.56 ) |
| General disorders and administration site conditions | 642 | 5584 | 10741671 | 50988568 | 0.55 ( 0.5 - 0.59 ) | 0.59 ( 217.7 ) | 0.59 ( 0.55 ) | -0.75 ( -0.87 ) |
| Injury, poisoning and procedural complications | 612 | 5614 | 6616459 | 55113780 | 0.91 ( 0.84 - 0.99 ) | 0.92 ( 5.14 ) | 0.92 ( 0.84 ) | -0.12 ( -0.25 ) |
| Blood and lymphatic system disorders | 9 | 6217 | 1046480 | 60683759 | 0.08 ( 0.04 - 0.16 ) | 0.09 ( 89.84 ) | 0.09 ( 0.04 ) | -3.55 ( -4.33 ) |
| Infections and infestations | 87 | 6139 | 3254288 | 58475951 | 0.25 ( 0.21 - 0.31 ) | 0.27 ( 187.14 ) | 0.27 ( 0.21 ) | -1.92 ( -2.21 ) |
| Gastrointestinal disorders | 311 | 5915 | 5260165 | 56470074 | 0.56 ( 0.5 - 0.63 ) | 0.59 ( 99.3 ) | 0.59 ( 0.52 ) | -0.77 ( -0.94 ) |
| Ear and labyrinth disorders | 25 | 6201 | 265044 | 61465195 | 0.93 ( 0.63 - 1.38 ) | 0.94 ( 0.11 ) | 0.94 ( 0.63 ) | -0.1 ( -0.66 ) |
| Renal and urinary disorders | 20 | 6206 | 1149207 | 60581032 | 0.17 ( 0.11 - 0.26 ) | 0.17 ( 80.86 ) | 0.17 ( 0.11 ) | -2.53 ( -3.11 ) |
| Skin and subcutaneous tissue disorders | 241 | 5985 | 3388365 | 58341874 | 0.69 ( 0.61 - 0.79 ) | 0.71 ( 31.42 ) | 0.71 ( 0.62 ) | -0.5 ( -0.69 ) |
| Respiratory, thoracic and mediastinal disorders | 235 | 5991 | 2900096 | 58830143 | 0.8 ( 0.7 - 0.91 ) | 0.8 ( 11.86 ) | 0.8 ( 0.71 ) | -0.32 ( -0.51 ) |

Supplementary Table S3. Distribution of adverse events and signal detection results at the SOC level for Donepezil.

| SOC | a | b | c | d | ROR(95%CI) | PRR(X^2^) | EBGM(EBGM05) | IC(IC025) |
| --- | --- | --- | --- | --- | --- | --- | --- | --- |
| Eye disorders | 221 | 26293 | 1253302 | 60456649 | 0.41 ( 0.36 - 0.46 ) | 0.41 ( 191.03 ) | 0.41 ( 0.36 ) | -1.28 ( -1.48 ) |
| Investigations | 1305 | 25209 | 3740627 | 57969324 | 0.8 ( 0.76 - 0.85 ) | 0.81 ( 60.46 ) | 0.81 ( 0.77 ) | -0.3 ( -0.38 ) |
| Product issues | 90 | 26424 | 1058874 | 60651077 | 0.2 ( 0.16 - 0.24 ) | 0.2 ( 297.84 ) | 0.2 ( 0.16 ) | -2.34 ( -2.63 ) |
| Endocrine disorders | 63 | 26451 | 160889 | 61549062 | 0.91 ( 0.71 - 1.17 ) | 0.91 ( 0.54 ) | 0.91 ( 0.71 ) | -0.13 ( -0.49 ) |
| Vascular disorders | 650 | 25864 | 1295198 | 60414753 | 1.17 ( 1.08 - 1.27 ) | 1.17 ( 16.04 ) | 1.17 ( 1.08 ) | 0.22 ( 0.11 ) |
| Social circumstances | 143 | 26371 | 292427 | 61417524 | 1.14 ( 0.97 - 1.34 ) | 1.14 ( 2.41 ) | 1.14 ( 0.97 ) | 0.19 ( -0.06 ) |
| Hepatobiliary disorders | 239 | 26275 | 574754 | 61135197 | 0.97 ( 0.85 - 1.1 ) | 0.97 ( 0.26 ) | 0.97 ( 0.85 ) | -0.05 ( -0.23 ) |
| Nervous system disorders | 5171 | 21343 | 5149026 | 56560925 | 2.66 ( 2.58 - 2.74 ) | 2.34 ( 4313.01 ) | 2.34 ( 2.27 ) | 1.22 ( 1.18 ) |
| Immune system disorders | 44 | 26470 | 689675 | 61020276 | 0.15 ( 0.11 - 0.2 ) | 0.15 ( 217.27 ) | 0.15 ( 0.11 ) | -2.75 ( -3.15 ) |
| Cardiac disorders | 2412 | 24102 | 1575090 | 60134861 | 3.82 ( 3.66 - 3.98 ) | 3.56 ( 4559.09 ) | 3.56 ( 3.41 ) | 1.83 ( 1.77 ) |
| Psychiatric disorders | 4034 | 22480 | 3380467 | 58329484 | 3.1 ( 2.99 - 3.2 ) | 2.78 ( 4848.84 ) | 2.78 ( 2.68 ) | 1.47 ( 1.42 ) |
| Musculoskeletal and connective tissue disorders | 824 | 25690 | 3177745 | 58532206 | 0.59 ( 0.55 - 0.63 ) | 0.6 ( 226.22 ) | 0.6 ( 0.56 ) | -0.73 ( -0.83 ) |
| Congenital, familial and genetic disorders | 12 | 26502 | 181191 | 61528760 | 0.15 ( 0.09 - 0.27 ) | 0.15 ( 55.86 ) | 0.15 ( 0.09 ) | -2.7 ( -3.4 ) |
| Neoplasms benign, malignant and unspecified (incl cysts and polyps) | 56 | 26458 | 1579055 | 60130896 | 0.08 ( 0.06 - 0.1 ) | 0.08 ( 586.04 ) | 0.08 ( 0.06 ) | -3.6 ( -3.96 ) |
| Metabolism and nutrition disorders | 973 | 25541 | 1335010 | 60374941 | 1.72 ( 1.62 - 1.84 ) | 1.7 ( 284.06 ) | 1.7 ( 1.59 ) | 0.76 ( 0.67 ) |
| Reproductive system and breast disorders | 20 | 26494 | 535449 | 61174502 | 0.09 ( 0.06 - 0.13 ) | 0.09 ( 193.47 ) | 0.09 ( 0.06 ) | -3.52 ( -4.09 ) |
| Surgical and medical procedures | 74 | 26440 | 860342 | 60849609 | 0.2 ( 0.16 - 0.25 ) | 0.2 ( 239.79 ) | 0.2 ( 0.16 ) | -2.32 ( -2.64 ) |
| General disorders and administration site conditions | 2900 | 23614 | 10739413 | 50970538 | 0.58 ( 0.56 - 0.61 ) | 0.63 ( 770.81 ) | 0.63 ( 0.6 ) | -0.67 ( -0.73 ) |
| Injury, poisoning and procedural complications | 1981 | 24533 | 6615090 | 55094861 | 0.67 ( 0.64 - 0.7 ) | 0.7 ( 292.19 ) | 0.7 ( 0.67 ) | -0.52 ( -0.59 ) |
| Blood and lymphatic system disorders | 214 | 26300 | 1046275 | 60663676 | 0.47 ( 0.41 - 0.54 ) | 0.48 ( 125.51 ) | 0.48 ( 0.42 ) | -1.07 ( -1.26 ) |
| Infections and infestations | 399 | 26115 | 3253976 | 58455975 | 0.27 ( 0.25 - 0.3 ) | 0.29 ( 753.6 ) | 0.29 ( 0.26 ) | -1.81 ( -1.95 ) |
| Gastrointestinal disorders | 2876 | 23638 | 5257600 | 56452351 | 1.31 ( 1.26 - 1.36 ) | 1.27 ( 184.15 ) | 1.27 ( 1.22 ) | 0.35 ( 0.29 ) |
| Ear and labyrinth disorders | 70 | 26444 | 264999 | 61444952 | 0.61 ( 0.49 - 0.78 ) | 0.61 ( 16.96 ) | 0.61 ( 0.49 ) | -0.7 ( -1.04 ) |
| Renal and urinary disorders | 452 | 26062 | 1148775 | 60561176 | 0.91 ( 0.83 - 1 ) | 0.92 ( 3.57 ) | 0.92 ( 0.83 ) | -0.13 ( -0.26 ) |
| Skin and subcutaneous tissue disorders | 710 | 25804 | 3387896 | 58322055 | 0.47 ( 0.44 - 0.51 ) | 0.49 ( 404.04 ) | 0.49 ( 0.45 ) | -1.04 ( -1.14 ) |
| Respiratory, thoracic and mediastinal disorders | 581 | 25933 | 2899750 | 58810201 | 0.45 ( 0.42 - 0.49 ) | 0.47 ( 372.25 ) | 0.47 ( 0.43 ) | -1.1 ( -1.22 ) |

Supplementary Table S4. Distribution of adverse events and signal detection results at the SOC level for Galantamine.

| SOC | a | b | c | d | ROR(95%CI) | PRR(X^2^) | EBGM(EBGM05) | IC(IC025) |
| --- | --- | --- | --- | --- | --- | --- | --- | --- |
| Eye disorders | 38 | 5188 | 1253485 | 60477754 | 0.35 ( 0.26 - 0.49 ) | 0.36 ( 44.63 ) | 0.36 ( 0.26 ) | -1.48 ( -1.92 ) |
| Investigations | 270 | 4956 | 3741662 | 57989577 | 0.84 ( 0.75 - 0.95 ) | 0.85 ( 7.35 ) | 0.85 ( 0.75 ) | -0.23 ( -0.41 ) |
| Product issues | 31 | 5195 | 1058933 | 60672306 | 0.34 ( 0.24 - 0.49 ) | 0.35 ( 39.03 ) | 0.35 ( 0.24 ) | -1.53 ( -2.01 ) |
| Endocrine disorders | 9 | 5217 | 160943 | 61570296 | 0.66 ( 0.34 - 1.27 ) | 0.66 ( 1.57 ) | 0.66 ( 0.34 ) | -0.6 ( -1.46 ) |
| Vascular disorders | 123 | 5103 | 1295725 | 60435514 | 1.12 ( 0.94 - 1.34 ) | 1.12 ( 1.65 ) | 1.12 ( 0.94 ) | 0.17 ( -0.1 ) |
| Social circumstances | 17 | 5209 | 292553 | 61438686 | 0.69 ( 0.43 - 1.1 ) | 0.69 ( 2.45 ) | 0.69 ( 0.43 ) | -0.54 ( -1.2 ) |
| Hepatobiliary disorders | 66 | 5160 | 574927 | 61156312 | 1.36 ( 1.07 - 1.73 ) | 1.36 ( 6.23 ) | 1.36 ( 1.06 ) | 0.44 ( 0.08 ) |
| Nervous system disorders | 890 | 4336 | 5153307 | 56577932 | 2.25 ( 2.1 - 2.42 ) | 2.04 ( 514.8 ) | 2.04 ( 1.9 ) | 1.03 ( 0.92 ) |
| Immune system disorders | 6 | 5220 | 689713 | 61041526 | 0.1 ( 0.05 - 0.23 ) | 0.1 ( 47.54 ) | 0.1 ( 0.05 ) | -3.28 ( -4.18 ) |
| Cardiac disorders | 354 | 4872 | 1577148 | 60154091 | 2.77 ( 2.49 - 3.09 ) | 2.65 ( 373.56 ) | 2.65 ( 2.38 ) | 1.41 ( 1.24 ) |
| Psychiatric disorders | 560 | 4666 | 3383941 | 58347298 | 2.07 ( 1.9 - 2.26 ) | 1.95 ( 276.26 ) | 1.95 ( 1.79 ) | 0.97 ( 0.84 ) |
| Musculoskeletal and connective tissue disorders | 113 | 5113 | 3178456 | 58552783 | 0.41 ( 0.34 - 0.49 ) | 0.42 ( 95.44 ) | 0.42 ( 0.35 ) | -1.25 ( -1.52 ) |
| Congenital, familial and genetic disorders | 2 | 5224 | 181201 | 61550038 | 0.13 ( 0.03 - 0.52 ) | 0.13 ( 11.63 ) | 0.13 ( 0.03 ) | -2.94 ( -4.11 ) |
| Neoplasms benign, malignant and unspecified (incl cysts and polyps) | 23 | 5203 | 1579088 | 60152151 | 0.17 ( 0.11 - 0.25 ) | 0.17 ( 94.04 ) | 0.17 ( 0.11 ) | -2.54 ( -3.08 ) |
| Metabolism and nutrition disorders | 217 | 5009 | 1335766 | 60395473 | 1.96 ( 1.71 - 2.24 ) | 1.92 ( 97.59 ) | 1.92 ( 1.67 ) | 0.94 ( 0.73 ) |
| Reproductive system and breast disorders | 4 | 5222 | 535465 | 61195774 | 0.09 ( 0.03 - 0.23 ) | 0.09 ( 38.01 ) | 0.09 ( 0.03 ) | -3.5 ( -4.5 ) |
| Surgical and medical procedures | 146 | 5080 | 860270 | 60870969 | 2.03 ( 1.73 - 2.4 ) | 2 ( 74.54 ) | 2 ( 1.7 ) | 1 ( 0.75 ) |
| General disorders and administration site conditions | 531 | 4695 | 10741782 | 50989457 | 0.54 ( 0.49 - 0.59 ) | 0.58 ( 190.59 ) | 0.58 ( 0.53 ) | -0.78 ( -0.91 ) |
| Injury, poisoning and procedural complications | 698 | 4528 | 6616373 | 55114866 | 1.28 ( 1.19 - 1.39 ) | 1.25 ( 38.01 ) | 1.25 ( 1.15 ) | 0.32 ( 0.2 ) |
| Blood and lymphatic system disorders | 30 | 5196 | 1046459 | 60684780 | 0.33 ( 0.23 - 0.48 ) | 0.34 ( 39.42 ) | 0.34 ( 0.24 ) | -1.56 ( -2.05 ) |
| Infections and infestations | 164 | 5062 | 3254211 | 58477028 | 0.58 ( 0.5 - 0.68 ) | 0.6 ( 47.63 ) | 0.6 ( 0.51 ) | -0.75 ( -0.97 ) |
| Gastrointestinal disorders | 498 | 4728 | 5259978 | 56471261 | 1.13 ( 1.03 - 1.24 ) | 1.12 ( 6.82 ) | 1.12 ( 1.02 ) | 0.16 ( 0.03 ) |
| Ear and labyrinth disorders | 19 | 5207 | 265050 | 61466189 | 0.85 ( 0.54 - 1.33 ) | 0.85 ( 0.53 ) | 0.85 ( 0.54 ) | -0.24 ( -0.88 ) |
| Renal and urinary disorders | 121 | 5105 | 1149106 | 60582133 | 1.25 ( 1.04 - 1.5 ) | 1.24 ( 5.89 ) | 1.24 ( 1.04 ) | 0.31 ( 0.05 ) |
| Skin and subcutaneous tissue disorders | 142 | 5084 | 3388464 | 58342775 | 0.48 ( 0.41 - 0.57 ) | 0.5 ( 77.4 ) | 0.5 ( 0.42 ) | -1.01 ( -1.25 ) |
| Respiratory, thoracic and mediastinal disorders | 154 | 5072 | 2900177 | 58831062 | 0.62 ( 0.52 - 0.72 ) | 0.63 ( 35.8 ) | 0.63 ( 0.53 ) | -0.67 ( -0.9 ) |

Supplementary Table S5. Distribution of adverse events and signal detection results at the SOC level for Lecanemab.

| SOC | a | b | c | d | ROR(95%CI) | PRR(X^2^) | EBGM(EBGM05) | IC(IC025) |
| --- | --- | --- | --- | --- | --- | --- | --- | --- |
| Eye disorders | 98 | 7293 | 1253425 | 60475649 | 0.65 ( 0.53 - 0.79 ) | 0.65 ( 18.44 ) | 0.65 ( 0.54 ) | -0.61 ( -0.9 ) |
| Investigations | 250 | 7141 | 3741682 | 57987392 | 0.54 ( 0.48 - 0.62 ) | 0.56 ( 93.15 ) | 0.56 ( 0.49 ) | -0.84 ( -1.02 ) |
| Product issues | 10 | 7381 | 1058954 | 60670120 | 0.08 ( 0.04 - 0.14 ) | 0.08 ( 109.46 ) | 0.08 ( 0.04 ) | -3.66 ( -4.41 ) |
| Endocrine disorders | 2 | 7389 | 160950 | 61568124 | 0.1 ( 0.03 - 0.41 ) | 0.1 ( 15.52 ) | 0.1 ( 0.03 ) | -3.27 ( -4.42 ) |
| Vascular disorders | 154 | 7237 | 1295694 | 60433380 | 0.99 ( 0.85 - 1.16 ) | 0.99 ( 0.01 ) | 0.99 ( 0.85 ) | -0.01 ( -0.24 ) |
| Social circumstances | 5 | 7386 | 292565 | 61436509 | 0.14 ( 0.06 - 0.34 ) | 0.14 ( 25.87 ) | 0.14 ( 0.06 ) | -2.81 ( -3.76 ) |
| Hepatobiliary disorders | 14 | 7377 | 574979 | 61154095 | 0.2 ( 0.12 - 0.34 ) | 0.2 ( 44.1 ) | 0.2 ( 0.12 ) | -2.3 ( -2.97 ) |
| Nervous system disorders | 2925 | 4466 | 5151272 | 56577802 | 7.19 ( 6.87 - 7.54 ) | 4.74 ( 9419.82 ) | 4.74 ( 4.52 ) | 2.24 ( 2.18 ) |
| Immune system disorders | 25 | 7366 | 689694 | 61039380 | 0.3 ( 0.2 - 0.44 ) | 0.3 ( 40.6 ) | 0.3 ( 0.2 ) | -1.72 ( -2.25 ) |
| Cardiac disorders | 138 | 7253 | 1577364 | 60151710 | 0.73 ( 0.61 - 0.86 ) | 0.73 ( 14.06 ) | 0.73 ( 0.62 ) | -0.45 ( -0.7 ) |
| Psychiatric disorders | 459 | 6932 | 3384042 | 58345032 | 1.14 ( 1.04 - 1.25 ) | 1.13 ( 7.56 ) | 1.13 ( 1.03 ) | 0.18 ( 0.04 ) |
| Musculoskeletal and connective tissue disorders | 192 | 7199 | 3178377 | 58550697 | 0.49 ( 0.43 - 0.57 ) | 0.5 ( 98.49 ) | 0.5 ( 0.44 ) | -0.99 ( -1.19 ) |
| Congenital, familial and genetic disorders | 2 | 7389 | 181201 | 61547873 | 0.09 ( 0.02 - 0.37 ) | 0.09 ( 17.93 ) | 0.09 ( 0.02 ) | -3.44 ( -4.59 ) |
| Neoplasms benign, malignant and unspecified (incl cysts and polyps) | 68 | 7323 | 1579043 | 60150031 | 0.35 ( 0.28 - 0.45 ) | 0.36 ( 79.55 ) | 0.36 ( 0.28 ) | -1.48 ( -1.81 ) |
| Metabolism and nutrition disorders | 98 | 7293 | 1335885 | 60393189 | 0.61 ( 0.5 - 0.74 ) | 0.61 ( 24.52 ) | 0.61 ( 0.5 ) | -0.71 ( -0.99 ) |
| Reproductive system and breast disorders | 9 | 7382 | 535460 | 61193614 | 0.14 ( 0.07 - 0.27 ) | 0.14 ( 47.79 ) | 0.14 ( 0.07 ) | -2.83 ( -3.62 ) |
| Surgical and medical procedures | 14 | 7377 | 860402 | 60868672 | 0.13 ( 0.08 - 0.23 ) | 0.14 ( 78.01 ) | 0.14 ( 0.08 ) | -2.88 ( -3.54 ) |
| General disorders and administration site conditions | 1320 | 6071 | 10740993 | 50988081 | 1.03 ( 0.97 - 1.1 ) | 1.03 ( 1.08 ) | 1.03 ( 0.97 ) | 0.04 ( -0.05 ) |
| Injury, poisoning and procedural complications | 606 | 6785 | 6616465 | 55112609 | 0.74 ( 0.68 - 0.81 ) | 0.76 ( 49.02 ) | 0.76 ( 0.7 ) | -0.39 ( -0.51 ) |
| Blood and lymphatic system disorders | 20 | 7371 | 1046469 | 60682605 | 0.16 ( 0.1 - 0.24 ) | 0.16 ( 90.01 ) | 0.16 ( 0.1 ) | -2.65 ( -3.22 ) |
| Infections and infestations | 197 | 7194 | 3254178 | 58474896 | 0.49 ( 0.43 - 0.57 ) | 0.51 ( 100.53 ) | 0.51 ( 0.44 ) | -0.98 ( -1.19 ) |
| Gastrointestinal disorders | 403 | 6988 | 5260073 | 56469001 | 0.62 ( 0.56 - 0.68 ) | 0.64 ( 89.28 ) | 0.64 ( 0.58 ) | -0.64 ( -0.79 ) |
| Ear and labyrinth disorders | 25 | 7366 | 265044 | 61464030 | 0.79 ( 0.53 - 1.17 ) | 0.79 ( 1.44 ) | 0.79 ( 0.53 ) | -0.34 ( -0.9 ) |
| Renal and urinary disorders | 50 | 7341 | 1149177 | 60579897 | 0.36 ( 0.27 - 0.47 ) | 0.36 ( 56.82 ) | 0.36 ( 0.28 ) | -1.46 ( -1.85 ) |
| Skin and subcutaneous tissue disorders | 170 | 7221 | 3388436 | 58340638 | 0.41 ( 0.35 - 0.47 ) | 0.42 ( 144.89 ) | 0.42 ( 0.36 ) | -1.25 ( -1.47 ) |
| Respiratory, thoracic and mediastinal disorders | 137 | 7254 | 2900194 | 58828880 | 0.38 ( 0.32 - 0.45 ) | 0.39 ( 133.57 ) | 0.39 ( 0.33 ) | -1.34 ( -1.58 ) |

Supplementary Table S6. Distribution of adverse events and signal detection results at the SOC level for Memantine.

| SOC | a | b | c | d | ROR(95%CI) | PRR(X^2^) | EBGM(EBGM05) | IC(IC025) |
| --- | --- | --- | --- | --- | --- | --- | --- | --- |
| Eye disorders | 285 | 21907 | 1253238 | 60461035 | 0.63 ( 0.56 - 0.71 ) | 0.63 ( 62.14 ) | 0.63 ( 0.56 ) | -0.66 ( -0.83 ) |
| Investigations | 1301 | 20891 | 3740631 | 57973642 | 0.97 ( 0.91 - 1.02 ) | 0.97 ( 1.54 ) | 0.97 ( 0.91 ) | -0.05 ( -0.13 ) |
| Product issues | 51 | 22141 | 1058913 | 60655360 | 0.13 ( 0.1 - 0.17 ) | 0.13 ( 290.58 ) | 0.13 ( 0.1 ) | -2.9 ( -3.28 ) |
| Endocrine disorders | 64 | 22128 | 160888 | 61553385 | 1.11 ( 0.87 - 1.41 ) | 1.11 ( 0.65 ) | 1.11 ( 0.87 ) | 0.15 ( -0.22 ) |
| Vascular disorders | 483 | 21709 | 1295365 | 60418908 | 1.04 ( 0.95 - 1.14 ) | 1.04 ( 0.65 ) | 1.04 ( 0.95 ) | 0.05 ( -0.08 ) |
| Social circumstances | 116 | 22076 | 292454 | 61421819 | 1.1 ( 0.92 - 1.32 ) | 1.1 ( 1.12 ) | 1.1 ( 0.92 ) | 0.14 ( -0.13 ) |
| Hepatobiliary disorders | 191 | 22001 | 574802 | 61139471 | 0.92 ( 0.8 - 1.06 ) | 0.92 ( 1.2 ) | 0.92 ( 0.8 ) | -0.11 ( -0.32 ) |
| Nervous system disorders | 4608 | 17584 | 5149589 | 56564684 | 2.88 ( 2.79 - 2.97 ) | 2.49 ( 4472.24 ) | 2.49 ( 2.41 ) | 1.31 ( 1.27 ) |
| Immune system disorders | 43 | 22149 | 689676 | 61024597 | 0.17 ( 0.13 - 0.23 ) | 0.17 ( 171.36 ) | 0.17 ( 0.13 ) | -2.53 ( -2.94 ) |
| Cardiac disorders | 766 | 21426 | 1576736 | 60137537 | 1.36 ( 1.27 - 1.47 ) | 1.35 ( 71.65 ) | 1.35 ( 1.26 ) | 0.43 ( 0.33 ) |
| Psychiatric disorders | 3876 | 18316 | 3380625 | 58333648 | 3.65 ( 3.53 - 3.78 ) | 3.19 ( 6152.6 ) | 3.19 ( 3.08 ) | 1.67 ( 1.62 ) |
| Congenital, familial and genetic disorders | 7 | 22185 | 181196 | 61533077 | 0.11 ( 0.05 - 0.22 ) | 0.11 ( 52.06 ) | 0.11 ( 0.05 ) | -3.22 ( -4.07 ) |
| Musculoskeletal and connective tissue disorders | 563 | 21629 | 3178006 | 58536267 | 0.48 ( 0.44 - 0.52 ) | 0.49 ( 310.06 ) | 0.49 ( 0.45 ) | -1.02 ( -1.14 ) |
| Neoplasms benign, malignant and unspecified (incl cysts and polyps) | 213 | 21979 | 1578898 | 60135375 | 0.37 ( 0.32 - 0.42 ) | 0.38 ( 227.46 ) | 0.38 ( 0.33 ) | -1.41 ( -1.61 ) |
| Metabolism and nutrition disorders | 688 | 21504 | 1335295 | 60378978 | 1.45 ( 1.34 - 1.56 ) | 1.43 ( 91.9 ) | 1.43 ( 1.33 ) | 0.52 ( 0.41 ) |
| Reproductive system and breast disorders | 34 | 22158 | 535435 | 61178838 | 0.18 ( 0.13 - 0.25 ) | 0.18 ( 131.68 ) | 0.18 ( 0.13 ) | -2.5 ( -2.95 ) |
| Surgical and medical procedures | 75 | 22117 | 860341 | 60853932 | 0.24 ( 0.19 - 0.3 ) | 0.24 ( 180.05 ) | 0.24 ( 0.19 ) | -2.04 ( -2.36 ) |
| General disorders and administration site conditions | 2916 | 19276 | 10739397 | 50974876 | 0.72 ( 0.69 - 0.75 ) | 0.76 ( 280.36 ) | 0.76 ( 0.73 ) | -0.41 ( -0.46 ) |
| Injury, poisoning and procedural complications | 1874 | 20318 | 6615197 | 55099076 | 0.77 ( 0.73 - 0.81 ) | 0.79 ( 119.94 ) | 0.79 ( 0.75 ) | -0.34 ( -0.41 ) |
| Blood and lymphatic system disorders | 166 | 22026 | 1046323 | 60667950 | 0.44 ( 0.38 - 0.51 ) | 0.44 ( 119.5 ) | 0.44 ( 0.38 ) | -1.18 ( -1.4 ) |
| Pregnancy, puerperium and perinatal conditions | 18 | 22174 | 257038 | 61457235 | 0.19 ( 0.12 - 0.31 ) | 0.19 ( 60.18 ) | 0.19 ( 0.12 ) | -2.36 ( -2.96 ) |
| Infections and infestations | 555 | 21637 | 3253820 | 58460453 | 0.46 ( 0.42 - 0.5 ) | 0.47 ( 341.24 ) | 0.47 ( 0.44 ) | -1.08 ( -1.2 ) |
| Gastrointestinal disorders | 1467 | 20725 | 5259009 | 56455264 | 0.76 ( 0.72 - 0.8 ) | 0.78 ( 103.94 ) | 0.78 ( 0.74 ) | -0.37 ( -0.44 ) |
| Ear and labyrinth disorders | 95 | 22097 | 264974 | 61449299 | 1 ( 0.82 - 1.22 ) | 1 ( 0 ) | 1 ( 0.82 ) | 0 ( -0.3 ) |
| Renal and urinary disorders | 495 | 21697 | 1148732 | 60565541 | 1.2 ( 1.1 - 1.31 ) | 1.2 ( 16.55 ) | 1.2 ( 1.1 ) | 0.26 ( 0.13 ) |
| Skin and subcutaneous tissue disorders | 544 | 21648 | 3388062 | 58326211 | 0.43 ( 0.4 - 0.47 ) | 0.45 ( 394.84 ) | 0.45 ( 0.41 ) | -1.16 ( -1.29 ) |
| Respiratory, thoracic and mediastinal disorders | 698 | 21494 | 2899633 | 58814640 | 0.66 ( 0.61 - 0.71 ) | 0.67 ( 119.53 ) | 0.67 ( 0.62 ) | -0.58 ( -0.69 ) |

Supplementary Table S7. Distribution of adverse events and signal detection results at the SOC level for Rivastigmine.

| SOC | a | b | c | d | ROR(95%CI) | PRR(X^2^) | EBGM(EBGM05) | IC(IC025) |
| --- | --- | --- | --- | --- | --- | --- | --- | --- |
| Eye disorders | 347 | 46195 | 1253176 | 60436747 | 0.36 ( 0.33 - 0.4 ) | 0.37 ( 386.56 ) | 0.37 ( 0.33 ) | -1.45 ( -1.6 ) |
| Investigations | 2299 | 44243 | 3739633 | 57950290 | 0.81 ( 0.77 - 0.84 ) | 0.81 ( 102.89 ) | 0.81 ( 0.78 ) | -0.3 ( -0.36 ) |
| Product issues | 760 | 45782 | 1058204 | 60631719 | 0.95 ( 0.89 - 1.02 ) | 0.95 ( 1.87 ) | 0.95 ( 0.89 ) | -0.07 ( -0.18 ) |
| Endocrine disorders | 30 | 46512 | 160922 | 61529001 | 0.25 ( 0.17 - 0.35 ) | 0.25 ( 68.99 ) | 0.25 ( 0.17 ) | -2.02 ( -2.5 ) |
| Vascular disorders | 1065 | 45477 | 1294783 | 60395140 | 1.09 ( 1.03 - 1.16 ) | 1.09 ( 8.12 ) | 1.09 ( 1.03 ) | 0.12 ( 0.03 ) |
| Social circumstances | 129 | 46413 | 292441 | 61397482 | 0.58 ( 0.49 - 0.69 ) | 0.58 ( 38.22 ) | 0.58 ( 0.49 ) | -0.77 ( -1.02 ) |
| Hepatobiliary disorders | 267 | 46275 | 574726 | 61115197 | 0.61 ( 0.54 - 0.69 ) | 0.62 ( 64.59 ) | 0.62 ( 0.55 ) | -0.7 ( -0.87 ) |
| Nervous system disorders | 7751 | 38791 | 5146446 | 56543477 | 2.2 ( 2.14 - 2.25 ) | 2 ( 4198.57 ) | 1.99 ( 1.95 ) | 1 ( 0.96 ) |
| Immune system disorders | 211 | 46331 | 689508 | 61000415 | 0.4 ( 0.35 - 0.46 ) | 0.41 ( 185.8 ) | 0.41 ( 0.35 ) | -1.3 ( -1.5 ) |
| Cardiac disorders | 1839 | 44703 | 1575663 | 60114260 | 1.57 ( 1.5 - 1.64 ) | 1.55 ( 364.58 ) | 1.55 ( 1.48 ) | 0.63 ( 0.56 ) |
| Psychiatric disorders | 5154 | 41388 | 3379347 | 58310576 | 2.15 ( 2.09 - 2.21 ) | 2.02 ( 2810.56 ) | 2.02 ( 1.96 ) | 1.01 ( 0.97 ) |
| Musculoskeletal and connective tissue disorders | 1053 | 45489 | 3177516 | 58512407 | 0.43 ( 0.4 - 0.45 ) | 0.44 ( 794.46 ) | 0.44 ( 0.41 ) | -1.19 ( -1.28 ) |
| Congenital, familial and genetic disorders | 18 | 46524 | 181185 | 61508738 | 0.13 ( 0.08 - 0.21 ) | 0.13 ( 103.36 ) | 0.13 ( 0.08 ) | -2.92 ( -3.52 ) |
| Neoplasms benign, malignant and unspecified (incl cysts and polyps) | 508 | 46034 | 1578603 | 60111320 | 0.42 ( 0.39 - 0.46 ) | 0.43 ( 401.81 ) | 0.43 ( 0.39 ) | -1.23 ( -1.36 ) |
| Metabolism and nutrition disorders | 1285 | 45257 | 1334698 | 60355225 | 1.28 ( 1.21 - 1.36 ) | 1.28 ( 78.39 ) | 1.28 ( 1.21 ) | 0.35 ( 0.27 ) |
| Reproductive system and breast disorders | 54 | 46488 | 535415 | 61154508 | 0.13 ( 0.1 - 0.17 ) | 0.13 ( 305.78 ) | 0.13 ( 0.1 ) | -2.9 ( -3.27 ) |
| Surgical and medical procedures | 233 | 46309 | 860183 | 60829740 | 0.36 ( 0.31 - 0.4 ) | 0.36 ( 270.32 ) | 0.36 ( 0.32 ) | -1.48 ( -1.66 ) |
| General disorders and administration site conditions | 8912 | 37630 | 10733401 | 50956522 | 1.12 ( 1.1 - 1.15 ) | 1.1 ( 99.02 ) | 1.1 ( 1.08 ) | 0.14 ( 0.1 ) |
| Injury, poisoning and procedural complications | 4033 | 42509 | 6613038 | 55076885 | 0.79 ( 0.77 - 0.82 ) | 0.81 ( 205.14 ) | 0.81 ( 0.78 ) | -0.31 ( -0.35 ) |
| Blood and lymphatic system disorders | 215 | 46327 | 1046274 | 60643649 | 0.27 ( 0.24 - 0.31 ) | 0.27 ( 425.04 ) | 0.27 ( 0.24 ) | -1.88 ( -2.07 ) |
| Pregnancy, puerperium and perinatal conditions | 2 | 46540 | 257054 | 61432869 | 0.01 ( 0 - 0.04 ) | 0.01 ( 190.75 ) | 0.01 ( 0 ) | -6.6 ( -7.69 ) |
| Infections and infestations | 2100 | 44442 | 3252275 | 58437648 | 0.85 ( 0.81 - 0.89 ) | 0.86 ( 53.78 ) | 0.86 ( 0.82 ) | -0.22 ( -0.29 ) |
| Gastrointestinal disorders | 3892 | 42650 | 5256584 | 56433339 | 0.98 ( 0.95 - 1.01 ) | 0.98 ( 1.5 ) | 0.98 ( 0.95 ) | -0.03 ( -0.08 ) |
| Ear and labyrinth disorders | 161 | 46381 | 264908 | 61425015 | 0.8 ( 0.69 - 0.94 ) | 0.81 ( 7.58 ) | 0.81 ( 0.69 ) | -0.31 ( -0.54 ) |
| Renal and urinary disorders | 712 | 45830 | 1148515 | 60541408 | 0.82 ( 0.76 - 0.88 ) | 0.82 ( 28.05 ) | 0.82 ( 0.76 ) | -0.28 ( -0.39 ) |
| Skin and subcutaneous tissue disorders | 1937 | 44605 | 3386669 | 58303254 | 0.75 ( 0.71 - 0.78 ) | 0.76 ( 158.11 ) | 0.76 ( 0.72 ) | -0.4 ( -0.47 ) |
| Respiratory, thoracic and mediastinal disorders | 1575 | 44967 | 2898756 | 58791167 | 0.71 ( 0.68 - 0.75 ) | 0.72 ( 179.59 ) | 0.72 ( 0.69 ) | -0.47 ( -0.55 ) |

Supplementary Table S8. Distribution of adverse events and signal detection results at the Preferred erm (PT) level for Aducanumab.

| **PT** | **a** | **b** | **c** | **d** | **ROR(95%CI)** | **PRR(X^2^)** | **EBGM(EBGM05)** | **IC(IC025)** | **FDR** |
| --- | --- | --- | --- | --- | --- | --- | --- | --- | --- |
| Superficial siderosis of central nervous system | 23 | 1082 | 92 | 61735268 | 14264.16 (8997.56 - 22613.48) | 13967.28 (256961.56) | 11174.02 (7048.36) | 13.45 (3.93) | < 0.001 |
| ARIA-E | 143 | 962 | 943 | 61734417 | 9731.43 (8072.48 - 11731.3) | 8472.2 (1051765.86) | 7356.74 (6102.62) | 12.84 (6.87) | < 0.001 |
| ARIA-H | 101 | 1004 | 837 | 61734523 | 7419.77  (5981.2 - 9204.33) | 6741.67 (607420.39) | 6015.86 (4849.49) | 12.55 (6.34) | < 0.001 |
| ARIA | 17 | 1088 | 255 | 61735105 | 3782.79  (2306.93 - 6202.82) | 3724.61 (59329.31) | 3491.88 (2129.52) | 11.77 (3.46) | < 0.001 |
| Cerebral microhaemorrhage | 7 | 1098 | 404 | 61734956 | 974.19 (460.39 - 2061.42) | 968.03 (6647.08) | 951.56 (449.69) | 9.89 (1.96) | < 0.001 |
| Brain oedema | 14 | 1091 | 12533 | 61722827 | 63.2 (37.29 - 107.1) | 62.41 (845.17) | 62.34 (36.79) | 5.96 (2.86) | < 0.001 |
| Subarachnoid haemorrhage | 9 | 1096 | 10122 | 61725238 | 50.08 (25.98 - 96.53) | 49.68 (428.96) | 49.63 (25.75) | 5.63 (2.16) | < 0.001 |
| Cerebral haemorrhage | 27 | 1078 | 35395 | 61699965 | 43.66 (29.8 - 63.97) | 42.62 (1097.11) | 42.59 (29.06) | 5.41 (3.55) | < 0.001 |
| Posterior reversible encephalopathy syndrome | 3 | 1102 | 10182 | 61725178 | 16.5 (5.31 - 51.26) | 16.46 (43.56) | 16.46 (5.3) | 4.04 (0.31) | < 0.001 |
| Head injury | 8 | 1097 | 31263 | 61704097 | 14.39 (7.18 - 28.86) | 14.3 (98.96) | 14.29 (7.13) | 3.84 (1.56) | < 0.001 |
| Confusional state | 35 | 1070 | 160194 | 61575166 | 12.57 (8.98 - 17.61) | 12.21 (360.96) | 12.2 (8.72) | 3.61 (2.73) | < 0.001 |
| Subdural haematoma | 3 | 1102 | 14242 | 61721118 | 11.8 (3.8 - 36.64) | 11.77 (29.56) | 11.77 (3.79) | 3.56 (0.23) | < 0.001 |
| Haemorrhage intracranial | 3 | 1102 | 14851 | 61720509 | 11.31 (3.64 - 35.14) | 11.29 (28.12) | 11.28 (3.63) | 3.5 (0.21) | < 0.001 |
| Mental status changes | 5 | 1100 | 27620 | 61707740 | 10.16 (4.22 - 24.45) | 10.11 (41.07) | 10.11 (4.2) | 3.34 (0.82) | < 0.001 |
| Ischaemic stroke | 3 | 1102 | 17680 | 61717680 | 9.5 (3.06 - 29.51) | 9.48 (22.76) | 9.48 (3.05) | 3.24 (0.16) | < 0.001 |
| Transient ischaemic attack | 5 | 1100 | 33658 | 61701702 | 8.33 (3.46 - 20.06) | 8.3 (32.11) | 8.3 (3.45) | 3.05 (0.72) | < 0.001 |
| Skin cancer | 3 | 1102 | 21247 | 61714113 | 7.91 (2.55 - 24.56) | 7.89 (18.05) | 7.89 (2.54) | 2.98 (0.09) | 0.001 |
| Aphasia | 4 | 1101 | 30437 | 61704923 | 7.37 (2.76 - 19.66) | 7.34 (21.92) | 7.34 (2.75) | 2.88 (0.4) | < 0.001 |
| Cerebral infarction | 3 | 1102 | 24310 | 61711050 | 6.91 (2.23 - 21.46) | 6.89 (15.12) | 6.89 (2.22) | 2.79 (0.03) | 0.002 |
| Seizure | 20 | 1085 | 171149 | 61564211 | 6.63 (4.26 - 10.32) | 6.53 (93.89) | 6.53 (4.19) | 2.71 (1.73) | < 0.001 |
| Atrial fibrillation | 11 | 1094 | 96720 | 61638640 | 6.41 (3.54 - 11.61) | 6.35 (49.7) | 6.35 (3.51) | 2.67 (1.3) | < 0.001 |
| Fall | 23 | 1082 | 328946 | 61406414 | 3.97 (2.63 - 6) | 3.91 (50) | 3.91 (2.58) | 1.97 (1.2) | < 0.001 |
| Headache | 41 | 1064 | 618819 | 61116541 | 3.81 (2.79 - 5.2) | 3.7 (81.66) | 3.7 (2.71) | 1.89 (1.34) | < 0.001 |

Supplementary Table S9. Distribution of adverse events and signal detection results at the Preferred erm (PT) level for Donanemab.

| **PT** | **a** | **b** | **c** | **d** | **ROR(95%CI)** | **PRR(X^2^)** | **EBGM(EBGM05)** | **IC(IC025)** | **FDR** |
| --- | --- | --- | --- | --- | --- | --- | --- | --- | --- |
| ARIA | 155 | 6071 | 117 | 61730122 | 13470.47 (10581.91 - 17147.52) | 13135.14 (875626.95) | 5650.61 (4438.92) | 12.46 (6.95) | < 0.001 |
| ARIA-E | 452 | 5774 | 634 | 61729605 | 7621.95 (6737.22 - 8622.86) | 7068.68 (1864750.1) | 4127.06 (3648.01) | 12.01 (8.51) | < 0.001 |
| ARIA-H | 369 | 5857 | 569 | 61729670 | 6834.91 (5980.82 - 7810.97) | 6429.88 (1438832.5) | 3900.82 (3413.38) | 11.93 (8.22) | < 0.001 |
| Cerebral microhaemorrhage | 88 | 6138 | 323 | 61729916 | 2739.99 (2161.81 - 3472.81) | 2701.28 (186678.22) | 2123.11 (1675.1) | 11.05 (6.08) | < 0.001 |
| Superficial siderosis of central nervous system | 16 | 6210 | 99 | 61730140 | 1606.54 (946.85 - 2725.85) | 1602.41 (22043.96) | 1379.61 (813.1) | 10.43 (3.32) | < 0.001 |
| Hippocampal atrophy | 4 | 6222 | 28 | 61730211 | 1417.33 (496.99 - 4041.96) | 1416.42 (4950.46) | 1239.49 (434.63) | 10.28 (0.93) | < 0.001 |
| Infusion related hypersensitivity reaction | 30 | 6196 | 541 | 61729698 | 552.47 (382.19 - 798.61) | 549.81 (15571.05) | 520.98 (360.41) | 9.03 (4.34) | < 0.001 |
| Haemorrhagic hepatic cyst | 3 | 6223 | 91 | 61730148 | 327.02 (103.51 - 1033.19) | 326.87 (943.5) | 316.47 (100.17) | 8.31 (0.51) | < 0.001 |
| Cerebral amyloid angiopathy | 5 | 6221 | 315 | 61729924 | 157.51 (65.08 - 381.18) | 157.38 (764.79) | 154.94 (64.02) | 7.28 (1.35) | < 0.001 |
| Cerebral small vessel ischaemic disease | 7 | 6219 | 640 | 61729599 | 108.57 (51.53 - 228.75) | 108.44 (737.12) | 107.28 (50.92) | 6.75 (1.88) | < 0.001 |
| Meningeal disorder | 3 | 6223 | 398 | 61729841 | 74.77 (24.01 - 232.89) | 74.74 (216.62) | 74.18 (23.82) | 6.21 (0.49) | < 0.001 |
| Lacunar infarction | 16 | 6210 | 2413 | 61727826 | 65.91 (40.29 - 107.83) | 65.74 (1013.46) | 65.32 (39.92) | 6.03 (3.07) | < 0.001 |
| Brain oedema | 69 | 6157 | 12478 | 61717761 | 55.43 (43.69 - 70.32) | 54.83 (3626.99) | 54.53 (42.98) | 5.77 (4.6) | < 0.001 |
| Infusion related reaction | 342 | 5884 | 64933 | 61665306 | 55.2 (49.48 - 61.57) | 52.22 (17110.37) | 51.95 (46.57) | 5.7 (5.34) | < 0.001 |
| Cerebral haemorrhage | 162 | 6064 | 35260 | 61694979 | 46.74 (39.98 - 54.66) | 45.55 (7030.97) | 45.35 (38.78) | 5.5 (4.93) | < 0.001 |
| White matter lesion | 6 | 6220 | 1278 | 61728961 | 46.59 (20.88 - 103.95) | 46.55 (266.18) | 46.34 (20.77) | 5.53 (1.54) | < 0.001 |
| Vasogenic cerebral oedema | 3 | 6223 | 697 | 61729542 | 42.7 (13.73 - 132.74) | 42.68 (121.57) | 42.5 (13.67) | 5.41 (0.45) | < 0.001 |
| Magnetic resonance imaging abnormal | 16 | 6210 | 4480 | 61725759 | 35.5 (21.71 - 58.03) | 35.41 (533.15) | 35.29 (21.59) | 5.14 (2.85) | < 0.001 |
| Cerebellar infarction | 5 | 6221 | 1671 | 61728568 | 29.69 (12.34 - 71.45) | 29.67 (138.1) | 29.58 (12.29) | 4.89 (1.18) | < 0.001 |
| Magnetic resonance imaging head abnormal | 6 | 6220 | 2289 | 61727950 | 26.01 (11.67 - 57.99) | 25.99 (143.8) | 25.92 (11.63) | 4.7 (1.41) | < 0.001 |
| Ischaemic cerebral infarction | 3 | 6223 | 1154 | 61729085 | 25.79 (8.3 - 80.1) | 25.78 (71.26) | 25.71 (8.28) | 4.68 (0.39) | < 0.001 |
| Cerebral atrophy | 10 | 6216 | 4021 | 61726218 | 24.7 (13.27 - 45.96) | 24.66 (226.44) | 24.6 (13.22) | 4.62 (2.1) | < 0.001 |
| Slow response to stimuli | 3 | 6223 | 1274 | 61728965 | 23.36 (7.52 - 72.54) | 23.35 (64.02) | 23.3 (7.5) | 4.54 (0.38) | < 0.001 |
| Anaphylactoid reaction | 9 | 6217 | 4080 | 61726159 | 21.9 (11.38 - 42.14) | 21.87 (178.87) | 21.83 (11.34) | 4.45 (1.91) | < 0.001 |
| Blood pressure immeasurable | 3 | 6223 | 1529 | 61728710 | 19.46 (6.27 - 60.43) | 19.45 (52.41) | 19.42 (6.25) | 4.28 (0.35) | < 0.001 |
| Flushing | 189 | 6037 | 100687 | 61629552 | 19.16 (16.58 - 22.15) | 18.61 (3148.94) | 18.58 (16.07) | 4.22 (3.87) | < 0.001 |
| Subdural haematoma | 22 | 6204 | 14223 | 61716016 | 15.39 (10.12 - 23.39) | 15.34 (294.45) | 15.31 (10.07) | 3.94 (2.64) | < 0.001 |
| Cerebral infarction | 32 | 6194 | 24281 | 61705958 | 13.13 (9.27 - 18.59) | 13.07 (356.26) | 13.05 (9.22) | 3.71 (2.75) | < 0.001 |
| Ischaemic stroke | 23 | 6203 | 17660 | 61712579 | 12.96 (8.6 - 19.52) | 12.91 (252.52) | 12.9 (8.56) | 3.69 (2.52) | < 0.001 |
| Lung opacity | 3 | 6223 | 2538 | 61727701 | 11.72 (3.78 - 36.39) | 11.72 (29.38) | 11.71 (3.77) | 3.55 (0.23) | < 0.001 |
| Cerebral ischaemia | 6 | 6220 | 5188 | 61725051 | 11.48 (5.15 - 25.57) | 11.47 (57.26) | 11.45 (5.14) | 3.52 (1.11) | < 0.001 |
| Anaphylactic shock | 29 | 6197 | 25357 | 61704882 | 11.39 (7.91 - 16.4) | 11.34 (273.2) | 11.33 (7.86) | 3.5 (2.55) | < 0.001 |
| Migraine with aura | 3 | 6223 | 2684 | 61727555 | 11.09 (3.57 - 34.41) | 11.08 (27.49) | 11.07 (3.57) | 3.47 (0.21) | < 0.001 |
| Subdural haemorrhage | 3 | 6223 | 2733 | 61727506 | 10.89 (3.51 - 33.79) | 10.88 (26.9) | 10.87 (3.5) | 3.44 (0.2) | < 0.001 |
| Haemorrhagic stroke | 8 | 6218 | 7778 | 61722461 | 10.21 (5.1 - 20.43) | 10.2 (66.31) | 10.19 (5.09) | 3.35 (1.37) | < 0.001 |
| Infusion site extravasation | 6 | 6220 | 6140 | 61724099 | 9.7 (4.35 - 21.6) | 9.69 (46.71) | 9.68 (4.35) | 3.28 (1.02) | < 0.001 |
| Dizziness postural | 9 | 6217 | 9493 | 61720746 | 9.41 (4.89 - 18.1) | 9.4 (67.5) | 9.39 (4.88) | 3.23 (1.44) | < 0.001 |
| Anaphylactic reaction | 50 | 6176 | 54285 | 61675954 | 9.2 (6.96 - 12.15) | 9.13 (362.07) | 9.12 (6.91) | 3.19 (2.57) | < 0.001 |
| Brain fog | 13 | 6213 | 14386 | 61715853 | 8.98 (5.21 - 15.47) | 8.96 (91.87) | 8.95 (5.19) | 3.16 (1.74) | < 0.001 |
| Cerebral haematoma | 3 | 6223 | 3318 | 61726921 | 8.97 (2.89 - 27.83) | 8.96 (21.21) | 8.96 (2.89) | 3.16 (0.14) | < 0.001 |
| Fibrin d dimer increased | 3 | 6223 | 3542 | 61726697 | 8.4 (2.71 - 26.07) | 8.4 (19.53) | 8.39 (2.7) | 3.07 (0.11) | 0.001 |
| Chills | 94 | 6132 | 115979 | 61614260 | 8.14 (6.64 - 9.98) | 8.04 (579.7) | 8.03 (6.55) | 3.01 (2.6) | < 0.001 |
| Head discomfort | 14 | 6212 | 17381 | 61712858 | 8 (4.74 - 13.52) | 7.99 (85.52) | 7.98 (4.72) | 3 (1.7) | < 0.001 |
| Subarachnoid haemorrhage | 8 | 6218 | 10123 | 61720116 | 7.84 (3.92 - 15.7) | 7.84 (47.68) | 7.83 (3.91) | 2.97 (1.19) | < 0.001 |
| Chest discomfort | 73 | 6153 | 100817 | 61629422 | 7.25 (5.76 - 9.14) | 7.18 (388.61) | 7.17 (5.7) | 2.84 (2.39) | < 0.001 |
| Feeling cold | 19 | 6207 | 27565 | 61702674 | 6.85 (4.37 - 10.75) | 6.83 (94.61) | 6.83 (4.35) | 2.77 (1.76) | < 0.001 |
| Feeling hot | 41 | 6185 | 60631 | 61669608 | 6.74 (4.96 - 9.17) | 6.7 (199.07) | 6.7 (4.93) | 2.74 (2.11) | < 0.001 |
| Blood pressure decreased | 43 | 6183 | 65634 | 61664605 | 6.53 (4.84 - 8.82) | 6.5 (200.02) | 6.49 (4.81) | 2.7 (2.09) | < 0.001 |
| Head injury | 18 | 6208 | 31253 | 61698986 | 5.72 (3.6 - 9.09) | 5.71 (69.94) | 5.71 (3.59) | 2.51 (1.53) | < 0.001 |
| Aphasia | 17 | 6209 | 30424 | 61699815 | 5.55 (3.45 - 8.94) | 5.54 (63.25) | 5.54 (3.44) | 2.47 (1.46) | < 0.001 |
| Confusional state | 88 | 6138 | 160141 | 61570098 | 5.51 (4.47 - 6.8) | 5.45 (320.27) | 5.45 (4.41) | 2.45 (2.07) | < 0.001 |
| Blood pressure increased | 79 | 6147 | 151413 | 61578826 | 5.23 (4.19 - 6.53) | 5.17 (266.46) | 5.17 (4.14) | 2.37 (1.97) | < 0.001 |
| Cold sweat | 9 | 6217 | 17463 | 61712776 | 5.12 (2.66 - 9.84) | 5.11 (29.74) | 5.11 (2.66) | 2.35 (0.94) | < 0.001 |
| Blood pressure systolic increased | 10 | 6216 | 19415 | 61710824 | 5.11 (2.75 - 9.51) | 5.11 (33.02) | 5.1 (2.74) | 2.35 (1.02) | < 0.001 |
| Oxygen saturation decreased | 28 | 6198 | 55174 | 61675065 | 5.05 (3.48 - 7.32) | 5.03 (90.49) | 5.03 (3.47) | 2.33 (1.61) | < 0.001 |
| Pallor | 13 | 6213 | 27022 | 61703217 | 4.78 (2.77 - 8.23) | 4.77 (38.73) | 4.77 (2.77) | 2.25 (1.14) | < 0.001 |
| Throat tightness | 12 | 6214 | 26678 | 61703561 | 4.47 (2.53 - 7.87) | 4.46 (32.21) | 4.46 (2.53) | 2.16 (1.01) | < 0.001 |
| Headache | 259 | 5967 | 618601 | 61111638 | 4.29 (3.79 - 4.86) | 4.15 (625.57) | 4.15 (3.66) | 2.05 (1.85) | < 0.001 |
| Hypersomnia | 12 | 6214 | 28276 | 61701963 | 4.21 (2.39 - 7.43) | 4.21 (29.35) | 4.21 (2.39) | 2.07 (0.95) | < 0.001 |
| Delirium | 13 | 6213 | 34222 | 61696017 | 3.77 (2.19 - 6.5) | 3.77 (26.42) | 3.77 (2.18) | 1.91 (0.88) | < 0.001 |
| Cerebrovascular accident | 63 | 6163 | 167897 | 61562342 | 3.75 (2.92 - 4.8) | 3.72 (125.61) | 3.72 (2.9) | 1.9 (1.47) | < 0.001 |
| Heart rate decreased | 13 | 6213 | 34881 | 61695358 | 3.7 (2.15 - 6.38) | 3.7 (25.56) | 3.69 (2.14) | 1.89 (0.86) | < 0.001 |
| Erythema | 70 | 6156 | 208156 | 61522083 | 3.36 (2.66 - 4.25) | 3.33 (114.74) | 3.33 (2.63) | 1.74 (1.35) | < 0.001 |
| Tremor | 54 | 6172 | 164312 | 61565927 | 3.28 (2.51 - 4.29) | 3.26 (84.73) | 3.26 (2.49) | 1.7 (1.25) | < 0.001 |
| Back pain | 74 | 6152 | 229929 | 61500310 | 3.22 (2.56 - 4.05) | 3.19 (111.7) | 3.19 (2.54) | 1.67 (1.3) | < 0.001 |
| Dizziness | 122 | 6104 | 492088 | 61238151 | 2.49 (2.08 - 2.98) | 2.46 (106.35) | 2.46 (2.05) | 1.3 (1.02) | < 0.001 |

Supplementary Table S10. Distribution of adverse events and signal detection results at the Preferred erm (PT) level for Donepezil.

| **PT** | **a** | **b** | **c** | **d** | **ROR(95%CI)** | **PRR(X^2^)** | **EBGM(EBGM05)** | **IC(IC025)** | **FDR** |
| --- | --- | --- | --- | --- | --- | --- | --- | --- | --- |
| Paratonia | 6 | 26508 | 15 | 61709936 | 931.19  (361.27 - 2400.21) | 930.98 (3981.35) | 665.27 (258.1) | 9.38 (1.54) | < 0.001 |
| Wandering pacemaker | 12 | 26502 | 35 | 61709916 | 798.34  (414.37 - 1538.11) | 797.98 (7113.05) | 594.5 (308.57) | 9.22 (2.77) | < 0.001 |
| Urea cycle disorder | 7 | 26507 | 40 | 61709911 | 407.41 (182.49 - 909.53) | 407.3 (2414.59) | 346.79 (155.34) | 8.44 (1.87) | < 0.001 |
| Cholinergic syndrome | 84 | 26430 | 548 | 61709403 | 357.89 (284.36 - 450.43) | 356.76 (25839.72) | 309.48 (245.9) | 8.27 (5.73) | < 0.001 |
| Pleurothotonus | 116 | 26398 | 786 | 61709165 | 345 (283.78 - 419.41) | 343.49 (34519.41) | 299.45 (246.32) | 8.23 (6.11) | < 0.001 |
| Overconfidence | 5 | 26509 | 36 | 61709915 | 323.32 (126.87 - 823.98) | 323.26 (1410.41) | 283.96 (111.42) | 8.15 (1.3) | < 0.001 |
| Trifascicular block | 7 | 26507 | 101 | 61709850 | 161.35 (75 - 347.14) | 161.31 (1042.92) | 150.92 (70.15) | 7.24 (1.88) | < 0.001 |
| Dropped head syndrome | 24 | 26490 | 360 | 61709591 | 155.3 (102.72 - 234.81) | 155.16 (3446.34) | 145.53 (96.25) | 7.19 (3.83) | < 0.001 |
| Poriomania | 12 | 26502 | 180 | 61709771 | 155.23 (86.53 - 278.5) | 155.16 (1723.16) | 145.53 (81.12) | 7.19 (2.76) | < 0.001 |
| Jealous delusion | 17 | 26497 | 264 | 61709687 | 149.97 (91.82 - 244.94) | 149.87 (2361.88) | 140.87 (86.25) | 7.14 (3.3) | < 0.001 |
| Adams-stokes syndrome | 9 | 26505 | 146 | 61709805 | 143.52 (73.2 - 281.4) | 143.47 (1199.39) | 135.2 (68.96) | 7.08 (2.29) | < 0.001 |
| Loose associations | 4 | 26510 | 65 | 61709886 | 143.25 (52.19 - 393.22) | 143.23 (532.19) | 134.98 (49.17) | 7.08 (0.94) | < 0.001 |
| Blood cholinesterase decreased | 15 | 26499 | 263 | 61709688 | 132.82 (78.93 - 223.5) | 132.74 (1855.46) | 125.64 (74.66) | 6.97 (3.09) | < 0.001 |
| Automatism | 11 | 26503 | 206 | 61709745 | 124.33 (67.78 - 228.06) | 124.28 (1277) | 118.03 (64.35) | 6.88 (2.6) | < 0.001 |
| Non-24-hour sleep-wake disorder | 7 | 26507 | 132 | 61709819 | 123.46 (57.72 - 264.06) | 123.43 (807.23) | 117.26 (54.82) | 6.87 (1.87) | < 0.001 |
| Prosopagnosia | 8 | 26506 | 151 | 61709800 | 123.35 (60.57 - 251.18) | 123.31 (921.7) | 117.15 (57.53) | 6.87 (2.09) | < 0.001 |
| Athetosis | 8 | 26506 | 166 | 61709785 | 112.2 (55.19 - 228.11) | 112.17 (840.88) | 107.06 (52.66) | 6.74 (2.08) | < 0.001 |
| Sexually inappropriate behaviour | 19 | 26495 | 434 | 61709517 | 101.97 (64.4 - 161.45) | 101.89 (1818.55) | 97.66 (61.68) | 6.61 (3.41) | < 0.001 |
| Bifascicular block | 8 | 26506 | 183 | 61709768 | 101.78 (50.14 - 206.61) | 101.75 (764.63) | 97.53 (48.04) | 6.61 (2.07) | < 0.001 |
| Postmortem blood drug level increased | 14 | 26500 | 344 | 61709607 | 94.77 (55.53 - 161.74) | 94.72 (1247.49) | 91.06 (53.35) | 6.51 (2.94) | < 0.001 |
| Hypocapnia | 15 | 26499 | 401 | 61709550 | 87.11 (52.02 - 145.88) | 87.06 (1230.09) | 83.96 (50.14) | 6.39 (3.03) | < 0.001 |
| Pressure of speech | 23 | 26491 | 621 | 61709330 | 86.28 (56.89 - 130.83) | 86.2 (1867.75) | 83.16 (54.84) | 6.38 (3.63) | < 0.001 |
| Visuospatial deficit | 3 | 26511 | 83 | 61709868 | 84.13 (26.59 - 266.23) | 84.12 (237.81) | 81.22 (25.67) | 6.34 (0.47) | < 0.001 |
| Neuromuscular blockade | 7 | 26507 | 219 | 61709732 | 74.41 (35.06 - 157.95) | 74.39 (491.15) | 72.12 (33.98) | 6.17 (1.83) | < 0.001 |
| Electrocardiogram pr prolongation | 26 | 26488 | 815 | 61709136 | 74.32 (50.29 - 109.84) | 74.25 (1820.79) | 71.99 (48.71) | 6.17 (3.75) | < 0.001 |
| Bradyarrhythmia | 50 | 26464 | 1581 | 61708370 | 73.74 (55.63 - 97.75) | 73.61 (3471.33) | 71.38 (53.85) | 6.16 (4.5) | < 0.001 |
| Flight of ideas | 16 | 26498 | 509 | 61709442 | 73.2 (44.5 - 120.43) | 73.16 (1104.1) | 70.96 (43.14) | 6.15 (3.08) | < 0.001 |
| Sinus node dysfunction | 91 | 26423 | 2915 | 61707036 | 72.9 (59.15 - 89.85) | 72.66 (6236.74) | 70.49 (57.19) | 6.14 (5.02) | < 0.001 |
| Progressive supranuclear palsy | 6 | 26508 | 193 | 61709758 | 72.37 (32.11 - 163.11) | 72.36 (409.49) | 70.2 (31.15) | 6.13 (1.58) | < 0.001 |
| Drop attacks | 17 | 26497 | 571 | 61709380 | 69.34 (42.8 - 112.34) | 69.29 (1111.16) | 67.32 (41.55) | 6.07 (3.15) | < 0.001 |
| Hyponatraemic syndrome | 9 | 26505 | 314 | 61709637 | 66.73 (34.4 - 129.47) | 66.71 (566.3) | 64.88 (33.44) | 6.02 (2.21) | < 0.001 |
| Brain compression | 11 | 26503 | 390 | 61709561 | 65.67 (36.07 - 119.59) | 65.65 (681.07) | 63.87 (35.08) | 6 (2.51) | < 0.001 |
| Rhythm idioventricular | 8 | 26506 | 317 | 61709634 | 58.75 (29.13 - 118.52) | 58.74 (442.86) | 57.32 (28.41) | 5.84 (2.01) | < 0.001 |
| Peptic ulcer perforation | 7 | 26507 | 298 | 61709653 | 54.69 (25.84 - 115.72) | 54.67 (360.37) | 53.44 (25.25) | 5.74 (1.79) | < 0.001 |
| Sinus bradycardia | 217 | 26297 | 9424 | 61700527 | 54.03 (47.2 - 61.84) | 53.59 (10949.26) | 52.41 (45.78) | 5.71 (5.21) | < 0.001 |
| Defect conduction intraventricular | 7 | 26507 | 314 | 61709637 | 51.9 (24.54 - 109.77) | 51.89 (341.72) | 50.78 (24.01) | 5.67 (1.78) | < 0.001 |
| Diabetic hyperosmolar coma | 9 | 26505 | 422 | 61709529 | 49.65 (25.65 - 96.11) | 49.64 (419.97) | 48.62 (25.12) | 5.6 (2.15) | < 0.001 |
| Grandiosity | 7 | 26507 | 355 | 61709596 | 45.91 (21.72 - 97) | 45.89 (301.46) | 45.03 (21.31) | 5.49 (1.76) | < 0.001 |
| Use of accessory respiratory muscles | 9 | 26505 | 463 | 61709488 | 45.26 (23.4 - 87.54) | 45.24 (381.95) | 44.4 (22.95) | 5.47 (2.13) | < 0.001 |
| Frontotemporal dementia | 8 | 26506 | 415 | 61709536 | 44.88 (22.29 - 90.35) | 44.87 (336.62) | 44.04 (21.87) | 5.46 (1.96) | < 0.001 |
| Hypersexuality | 33 | 26481 | 1779 | 61708172 | 43.23 (30.63 - 61.01) | 43.17 (1334.77) | 42.41 (30.05) | 5.41 (3.76) | < 0.001 |
| Decompensated hypothyroidism | 8 | 26506 | 431 | 61709520 | 43.21 (21.47 - 86.98) | 43.2 (323.78) | 42.43 (21.08) | 5.41 (1.95) | < 0.001 |
| Torticollis | 37 | 26477 | 2041 | 61707910 | 42.25 (30.52 - 58.5) | 42.19 (1461.57) | 41.46 (29.94) | 5.37 (3.85) | < 0.001 |
| Postural tremor | 5 | 26509 | 280 | 61709671 | 41.57 (17.17 - 100.66) | 41.56 (194.46) | 40.85 (16.87) | 5.35 (1.23) | < 0.001 |
| Atrioventricular block first degree | 74 | 26440 | 4277 | 61705674 | 40.38 (32.08 - 50.83) | 40.27 (2785.76) | 39.6 (31.46) | 5.31 (4.37) | < 0.001 |
| Thermal burns of eye | 13 | 26501 | 782 | 61709169 | 38.71 (22.37 - 66.98) | 38.69 (469.53) | 38.08 (22.01) | 5.25 (2.61) | < 0.001 |
| Executive dysfunction | 8 | 26506 | 501 | 61709450 | 37.18 (18.49 - 74.76) | 37.16 (277.11) | 36.6 (18.2) | 5.19 (1.91) | < 0.001 |
| Sinoatrial block | 9 | 26505 | 591 | 61709360 | 35.46 (18.35 - 68.49) | 35.44 (296.73) | 34.93 (18.08) | 5.13 (2.07) | < 0.001 |
| Bradycardia | 754 | 25760 | 51923 | 61658028 | 34.76 (32.31 - 37.39) | 33.8 (23674.73) | 33.33 (30.98) | 5.06 (4.89) | < 0.001 |
| Torsade de pointes | 114 | 26400 | 7668 | 61702283 | 34.75 (28.87 - 41.82) | 34.6 (3665.9) | 34.11 (28.34) | 5.09 (4.46) | < 0.001 |
| Intention tremor | 8 | 26506 | 556 | 61709395 | 33.5 (16.67 - 67.32) | 33.49 (248.57) | 33.03 (16.43) | 5.05 (1.89) | < 0.001 |
| Vascular dementia | 12 | 26502 | 856 | 61709095 | 32.64 (18.46 - 57.71) | 32.63 (362.82) | 32.19 (18.21) | 5.01 (2.44) | < 0.001 |
| Sinus arrest | 21 | 26493 | 1510 | 61708441 | 32.39 (21.05 - 49.84) | 32.37 (629.65) | 31.94 (20.76) | 5 (3.11) | < 0.001 |
| Delusion of grandeur | 6 | 26508 | 439 | 61709512 | 31.82 (14.22 - 71.22) | 31.81 (176.64) | 31.39 (14.03) | 4.97 (1.46) | < 0.001 |
| Long qt syndrome | 32 | 26482 | 2355 | 61707596 | 31.66 (22.33 - 44.89) | 31.63 (936.35) | 31.22 (22.02) | 4.96 (3.52) | < 0.001 |
| Selective eating disorder | 12 | 26502 | 887 | 61709064 | 31.5 (17.82 - 55.69) | 31.49 (349.51) | 31.08 (17.58) | 4.96 (2.42) | < 0.001 |
| Food refusal | 11 | 26503 | 822 | 61709129 | 31.16 (17.19 - 56.49) | 31.15 (316.72) | 30.75 (16.96) | 4.94 (2.3) | < 0.001 |
| Ventricular tachyarrhythmia | 4 | 26510 | 317 | 61709634 | 29.37 (10.96 - 78.75) | 29.37 (108.24) | 29.01 (10.82) | 4.86 (0.83) | < 0.001 |
| Neuropsychological symptoms | 19 | 26495 | 1514 | 61708437 | 29.23 (18.59 - 45.96) | 29.21 (511.21) | 28.86 (18.35) | 4.85 (2.94) | < 0.001 |
| Atrial tachycardia | 25 | 26489 | 2082 | 61707869 | 27.97 (18.85 - 41.5) | 27.95 (641.89) | 27.63 (18.62) | 4.79 (3.2) | < 0.001 |
| Atrioventricular block complete | 78 | 26436 | 6651 | 61703300 | 27.37 (21.89 - 34.23) | 27.3 (1953.2) | 26.99 (21.58) | 4.75 (4.02) | < 0.001 |
| Suspiciousness | 8 | 26506 | 683 | 61709268 | 27.27 (13.58 - 54.76) | 27.26 (200.04) | 26.96 (13.43) | 4.75 (1.83) | < 0.001 |
| Lack of spontaneous speech | 5 | 26509 | 435 | 61709516 | 26.76 (11.08 - 64.61) | 26.75 (122.54) | 26.46 (10.96) | 4.73 (1.15) | < 0.001 |
| Anticholinergic syndrome | 17 | 26497 | 1559 | 61708392 | 25.4 (15.74 - 40.96) | 25.38 (393.84) | 25.12 (15.57) | 4.65 (2.74) | < 0.001 |
| Antisocial behaviour | 10 | 26504 | 988 | 61708963 | 23.57 (12.64 - 43.94) | 23.56 (213.84) | 23.33 (12.51) | 4.54 (2.07) | < 0.001 |
| Skin oedema | 10 | 26504 | 992 | 61708959 | 23.47 (12.59 - 43.76) | 23.46 (212.91) | 23.24 (12.46) | 4.54 (2.07) | < 0.001 |
| Duodenal ulcer haemorrhage | 28 | 26486 | 2833 | 61707118 | 23.03 (15.87 - 33.42) | 23 (583.57) | 22.79 (15.7) | 4.51 (3.16) | < 0.001 |
| Neuroleptic malignant syndrome | 113 | 26401 | 11646 | 61698305 | 22.68 (18.83 - 27.3) | 22.58 (2308.92) | 22.38 (18.58) | 4.48 (3.96) | < 0.001 |
| Hallucination, olfactory | 4 | 26510 | 418 | 61709533 | 22.28 (8.32 - 59.63) | 22.27 (80.5) | 22.07 (8.24) | 4.46 (0.78) | < 0.001 |
| Chorea | 16 | 26498 | 1694 | 61708257 | 22 (13.44 - 35.99) | 21.98 (317.47) | 21.79 (13.31) | 4.45 (2.59) | < 0.001 |
| Sedation complication | 23 | 26491 | 2448 | 61707503 | 21.89 (14.51 - 33) | 21.87 (453.76) | 21.67 (14.37) | 4.44 (2.95) | < 0.001 |
| Atrioventricular block second degree | 28 | 26486 | 2994 | 61706957 | 21.79 (15.01 - 31.62) | 21.77 (549.63) | 21.57 (14.87) | 4.43 (3.12) | < 0.001 |
| Foaming at mouth | 18 | 26496 | 1976 | 61707975 | 21.22 (13.34 - 33.75) | 21.2 (343.36) | 21.02 (13.21) | 4.39 (2.69) | < 0.001 |
| Nodal rhythm | 13 | 26501 | 1453 | 61708498 | 20.83 (12.07 - 35.97) | 20.82 (243.16) | 20.65 (11.96) | 4.37 (2.33) | < 0.001 |
| Apraxia | 13 | 26501 | 1466 | 61708485 | 20.65 (11.96 - 35.65) | 20.64 (240.81) | 20.47 (11.85) | 4.36 (2.32) | < 0.001 |
| Parkinsonism | 84 | 26430 | 9550 | 61700401 | 20.53 (16.56 - 25.46) | 20.47 (1542.41) | 20.3 (16.37) | 4.34 (3.73) | < 0.001 |
| Mucosal haemorrhage | 10 | 26504 | 1135 | 61708816 | 20.51 (11.01 - 38.23) | 20.51 (183.93) | 20.34 (10.91) | 4.35 (2.01) | < 0.001 |
| Mania | 138 | 26376 | 16125 | 61693826 | 20.02 (16.92 - 23.68) | 19.92 (2459.3) | 19.76 (16.7) | 4.3 (3.88) | < 0.001 |
| Miosis | 67 | 26447 | 7813 | 61702138 | 20.01 (15.73 - 25.45) | 19.96 (1196.5) | 19.8 (15.56) | 4.31 (3.6) | < 0.001 |
| Libido increased | 17 | 26497 | 1988 | 61707963 | 19.91 (12.35 - 32.11) | 19.9 (302.62) | 19.74 (12.25) | 4.3 (2.59) | < 0.001 |
| Obsessive thoughts | 15 | 26499 | 1778 | 61708173 | 19.65 (11.82 - 32.66) | 19.64 (263.08) | 19.48 (11.72) | 4.28 (2.45) | < 0.001 |
| Neurodegenerative disorder | 5 | 26509 | 596 | 61709355 | 19.53 (8.1 - 47.1) | 19.53 (87.15) | 19.37 (8.03) | 4.28 (1.07) | < 0.001 |
| Hypocoagulable state | 8 | 26506 | 958 | 61708993 | 19.44 (9.69 - 38.99) | 19.44 (138.74) | 19.28 (9.61) | 4.27 (1.7) | < 0.001 |
| Hypomania | 30 | 26484 | 3626 | 61706325 | 19.28 (13.46 - 27.62) | 19.26 (515.02) | 19.11 (13.34) | 4.26 (3.07) | < 0.001 |
| Thirst decreased | 5 | 26509 | 607 | 61709344 | 19.18 (7.95 - 46.24) | 19.17 (85.42) | 19.02 (7.89) | 4.25 (1.06) | < 0.001 |
| Neuromuscular block prolonged | 3 | 26511 | 365 | 61709586 | 19.13 (6.14 - 59.6) | 19.13 (51.13) | 18.98 (6.09) | 4.25 (0.34) | < 0.001 |
| Facial spasm | 9 | 26505 | 1103 | 61708848 | 19 (9.86 - 36.61) | 18.99 (152.15) | 18.85 (9.78) | 4.24 (1.84) | < 0.001 |
| Cerebral atrophy | 32 | 26482 | 3999 | 61705952 | 18.65 (13.16 - 26.41) | 18.62 (529.49) | 18.48 (13.05) | 4.21 (3.09) | < 0.001 |
| Labelled drug-drug interaction issue | 11 | 26503 | 1375 | 61708576 | 18.63 (10.29 - 33.72) | 18.62 (181.95) | 18.48 (10.21) | 4.21 (2.07) | < 0.001 |
| Daydreaming | 12 | 26502 | 1518 | 61708433 | 18.41 (10.43 - 32.49) | 18.4 (195.89) | 18.26 (10.35) | 4.19 (2.17) | < 0.001 |
| Poverty of speech | 3 | 26511 | 384 | 61709567 | 18.19 (5.84 - 56.64) | 18.18 (48.34) | 18.05 (5.8) | 4.17 (0.33) | < 0.001 |
| Atrioventricular block | 59 | 26455 | 7594 | 61702357 | 18.12 (14.02 - 23.42) | 18.08 (944.91) | 17.95 (13.89) | 4.17 (3.43) | < 0.001 |
| Potentiating drug interaction | 26 | 26488 | 3467 | 61706484 | 17.47 (11.88 - 25.7) | 17.45 (400.32) | 17.33 (11.78) | 4.12 (2.87) | < 0.001 |
| Electrocardiogram qt prolonged | 266 | 26248 | 35963 | 61673988 | 17.38 (15.4 - 19.62) | 17.21 (4035.17) | 17.1 (15.14) | 4.1 (3.83) | < 0.001 |
| Delusion | 111 | 26403 | 15246 | 61694705 | 17.01 (14.11 - 20.51) | 16.95 (1653.84) | 16.83 (13.96) | 4.07 (3.61) | < 0.001 |
| Delusional disorder, persecutory type | 3 | 26511 | 414 | 61709537 | 16.87 (5.42 - 52.52) | 16.87 (44.45) | 16.75 (5.38) | 4.07 (0.31) | < 0.001 |
| Encephalomalacia | 4 | 26510 | 566 | 61709385 | 16.45 (6.15 - 43.99) | 16.45 (57.63) | 16.34 (6.11) | 4.03 (0.71) | < 0.001 |
| Myoclonus | 82 | 26432 | 11725 | 61698226 | 16.32 (13.13 - 20.29) | 16.28 (1167.83) | 16.17 (13.01) | 4.02 (3.45) | < 0.001 |
| Delirium | 236 | 26278 | 33999 | 61675952 | 16.29 (14.33 - 18.53) | 16.16 (3334.07) | 16.05 (14.11) | 4 (3.73) | < 0.001 |
| Inappropriate affect | 11 | 26503 | 1572 | 61708379 | 16.29 (9 - 29.48) | 16.29 (156.73) | 16.18 (8.94) | 4.02 (2) | < 0.001 |
| Multiple drug therapy | 5 | 26509 | 723 | 61709228 | 16.1 (6.68 - 38.8) | 16.1 (70.3) | 15.99 (6.64) | 4 (1.01) | < 0.001 |
| Acute abdomen | 10 | 26504 | 1448 | 61708503 | 16.08 (8.63 - 29.95) | 16.07 (140.39) | 15.97 (8.57) | 4 (1.88) | < 0.001 |
| Gastrointestinal ulcer | 15 | 26499 | 2182 | 61707769 | 16.01 (9.63 - 26.6) | 16 (209.5) | 15.9 (9.57) | 3.99 (2.32) | < 0.001 |
| Salivary hypersecretion | 67 | 26447 | 9819 | 61700132 | 15.92 (12.52 - 20.25) | 15.88 (928.09) | 15.78 (12.41) | 3.98 (3.34) | < 0.001 |
| Femoral neck fracture | 34 | 26480 | 5076 | 61704875 | 15.61 (11.14 - 21.87) | 15.59 (461.18) | 15.49 (11.06) | 3.95 (2.96) | < 0.001 |
| Muscle contractions involuntary | 23 | 26491 | 3478 | 61706473 | 15.4 (10.22 - 23.22) | 15.39 (307.48) | 15.3 (10.15) | 3.94 (2.67) | < 0.001 |
| Cerebellar haemorrhage | 9 | 26505 | 1412 | 61708539 | 14.84 (7.7 - 28.58) | 14.84 (115.39) | 14.75 (7.66) | 3.88 (1.72) | < 0.001 |
| Electrocardiogram t wave inversion | 14 | 26500 | 2204 | 61707747 | 14.79 (8.74 - 25.02) | 14.78 (178.8) | 14.7 (8.69) | 3.88 (2.19) | < 0.001 |
| Duodenal perforation | 6 | 26508 | 959 | 61708992 | 14.56 (6.53 - 32.5) | 14.56 (75.31) | 14.48 (6.49) | 3.86 (1.21) | < 0.001 |
| Conduction disorder | 11 | 26503 | 1810 | 61708141 | 14.15 (7.82 - 25.6) | 14.14 (133.56) | 14.07 (7.77) | 3.81 (1.92) | < 0.001 |
| Activities of daily living decreased | 3 | 26511 | 494 | 61709457 | 14.14 (4.54 - 43.98) | 14.13 (36.39) | 14.06 (4.52) | 3.81 (0.27) | < 0.001 |
| Theft | 4 | 26510 | 659 | 61709292 | 14.13 (5.29 - 37.76) | 14.13 (48.5) | 14.05 (5.26) | 3.81 (0.66) | < 0.001 |
| Drooling | 40 | 26474 | 6765 | 61703186 | 13.78 (10.1 - 18.81) | 13.76 (470.64) | 13.69 (10.03) | 3.77 (2.93) | < 0.001 |
| Supraventricular extrasystoles | 20 | 26494 | 3392 | 61706559 | 13.73 (8.85 - 21.32) | 13.72 (234.55) | 13.65 (8.79) | 3.77 (2.46) | < 0.001 |
| Sudden death | 61 | 26453 | 10540 | 61699411 | 13.5 (10.49 - 17.37) | 13.47 (700.27) | 13.4 (10.41) | 3.74 (3.11) | < 0.001 |
| Circulatory collapse | 97 | 26417 | 16867 | 61693084 | 13.43 (11 - 16.4) | 13.38 (1105.53) | 13.31 (10.9) | 3.73 (3.27) | < 0.001 |
| Skull fracture | 9 | 26505 | 1614 | 61708337 | 12.98 (6.74 - 25) | 12.98 (98.95) | 12.91 (6.71) | 3.69 (1.64) | < 0.001 |
| Substance-induced psychotic disorder | 12 | 26502 | 2172 | 61707779 | 12.86 (7.29 - 22.69) | 12.86 (130.52) | 12.79 (7.25) | 3.68 (1.94) | < 0.001 |
| Marital problem | 3 | 26511 | 546 | 61709405 | 12.79 (4.11 - 39.78) | 12.79 (32.42) | 12.72 (4.09) | 3.67 (0.25) | < 0.001 |
| Tonic clonic movements | 7 | 26507 | 1278 | 61708673 | 12.75 (6.07 - 26.8) | 12.75 (75.37) | 12.68 (6.03) | 3.66 (1.34) | < 0.001 |
| Gastrointestinal ulcer haemorrhage | 6 | 26508 | 1103 | 61708848 | 12.66 (5.68 - 28.25) | 12.66 (64.09) | 12.6 (5.65) | 3.66 (1.15) | < 0.001 |
| Persecutory delusion | 13 | 26501 | 2427 | 61707524 | 12.47 (7.23 - 21.51) | 12.47 (136.39) | 12.41 (7.19) | 3.63 (2) | < 0.001 |
| Bundle branch block | 5 | 26509 | 936 | 61709015 | 12.44 (5.16 - 29.95) | 12.43 (52.29) | 12.37 (5.14) | 3.63 (0.91) | < 0.001 |
| Syncope | 510 | 26004 | 99461 | 61610490 | 12.15 (11.13 - 13.26) | 11.93 (5091.41) | 11.88 (10.88) | 3.57 (3.41) | < 0.001 |
| Social avoidant behaviour | 29 | 26485 | 5723 | 61704228 | 11.81 (8.19 - 17.01) | 11.79 (285.06) | 11.74 (8.15) | 3.55 (2.58) | < 0.001 |
| Drowning | 5 | 26509 | 988 | 61708963 | 11.78 (4.89 - 28.37) | 11.78 (49.07) | 11.72 (4.87) | 3.55 (0.89) | < 0.001 |
| Duodenal ulcer | 35 | 26479 | 6967 | 61702984 | 11.71 (8.4 - 16.32) | 11.69 (340.55) | 11.64 (8.35) | 3.54 (2.68) | < 0.001 |
| Anal incontinence | 68 | 26446 | 13572 | 61696379 | 11.69 (9.21 - 14.84) | 11.66 (659.64) | 11.61 (9.14) | 3.54 (2.98) | < 0.001 |
| Muscle rigidity | 58 | 26456 | 11766 | 61698185 | 11.5 (8.88 - 14.88) | 11.47 (551.88) | 11.42 (8.82) | 3.51 (2.9) | < 0.001 |
| Systemic infection | 8 | 26506 | 1634 | 61708317 | 11.4 (5.69 - 22.83) | 11.4 (75.5) | 11.34 (5.66) | 3.5 (1.44) | < 0.001 |
| Hypothermia | 48 | 26466 | 9982 | 61699969 | 11.21 (8.44 - 14.89) | 11.19 (443.44) | 11.14 (8.39) | 3.48 (2.79) | < 0.001 |
| Slow response to stimuli | 6 | 26508 | 1271 | 61708680 | 10.99 (4.93 - 24.51) | 10.99 (54.21) | 10.94 (4.91) | 3.45 (1.08) | < 0.001 |
| Resting tremor | 3 | 26511 | 638 | 61709313 | 10.95 (3.52 - 34.03) | 10.94 (26.98) | 10.9 (3.51) | 3.45 (0.2) | < 0.001 |
| Subacute cutaneous lupus erythematosus | 8 | 26506 | 1733 | 61708218 | 10.75 (5.37 - 21.53) | 10.74 (70.37) | 10.7 (5.34) | 3.42 (1.4) | < 0.001 |
| Aggression | 222 | 26292 | 49417 | 61660534 | 10.54 (9.23 - 12.03) | 10.46 (1891.45) | 10.41 (9.12) | 3.38 (3.13) | < 0.001 |
| Nightmare | 152 | 26362 | 34211 | 61675740 | 10.39 (8.86 - 12.2) | 10.34 (1277.55) | 10.3 (8.78) | 3.36 (3.04) | < 0.001 |
| Fluid intake reduced | 21 | 26493 | 4865 | 61705086 | 10.05 (6.55 - 15.44) | 10.05 (170.35) | 10.01 (6.52) | 3.32 (2.21) | < 0.001 |
| Cerebral haematoma | 14 | 26500 | 3307 | 61706644 | 9.86 (5.83 - 16.67) | 9.85 (110.9) | 9.82 (5.81) | 3.3 (1.88) | < 0.001 |
| Slow speech | 10 | 26504 | 2363 | 61707588 | 9.85 (5.29 - 18.34) | 9.85 (79.18) | 9.81 (5.27) | 3.29 (1.57) | < 0.001 |
| Paradoxical drug reaction | 15 | 26499 | 3614 | 61706337 | 9.67 (5.82 - 16.05) | 9.66 (115.98) | 9.62 (5.8) | 3.27 (1.92) | < 0.001 |
| Judgement impaired | 6 | 26508 | 1449 | 61708502 | 9.64 (4.32 - 21.49) | 9.64 (46.26) | 9.6 (4.31) | 3.26 (1.01) | < 0.001 |
| Brain contusion | 3 | 26511 | 726 | 61709225 | 9.62 (3.09 - 29.9) | 9.62 (23.07) | 9.58 (3.08) | 3.26 (0.16) | 0.001 |
| Anorgasmia | 11 | 26503 | 2677 | 61707274 | 9.57 (5.29 - 17.3) | 9.56 (84.01) | 9.53 (5.27) | 3.25 (1.64) | < 0.001 |
| Posture abnormal | 13 | 26501 | 3208 | 61706743 | 9.44 (5.47 - 16.27) | 9.43 (97.6) | 9.4 (5.45) | 3.23 (1.78) | < 0.001 |
| Perforated ulcer | 3 | 26511 | 741 | 61709210 | 9.42 (3.03 - 29.29) | 9.42 (22.5) | 9.39 (3.02) | 3.23 (0.15) | 0.001 |
| Altered state of consciousness | 84 | 26430 | 21263 | 61688688 | 9.22 (7.44 - 11.43) | 9.19 (611.28) | 9.16 (7.39) | 3.2 (2.75) | < 0.001 |
| Gastritis haemorrhagic | 6 | 26508 | 1526 | 61708425 | 9.15 (4.11 - 20.41) | 9.15 (43.39) | 9.12 (4.09) | 3.19 (0.98) | < 0.001 |
| Physical assault | 8 | 26506 | 2055 | 61707896 | 9.06 (4.53 - 18.15) | 9.06 (57.15) | 9.03 (4.51) | 3.17 (1.29) | < 0.001 |
| Electrocardiogram t wave abnormal | 6 | 26508 | 1541 | 61708410 | 9.06 (4.07 - 20.21) | 9.06 (42.87) | 9.03 (4.05) | 3.17 (0.98) | < 0.001 |
| Hallucination, visual | 80 | 26434 | 20689 | 61689262 | 9.02 (7.24 - 11.24) | 9 (566.87) | 8.97 (7.2) | 3.16 (2.71) | < 0.001 |
| Behaviour disorder | 21 | 26493 | 5422 | 61704529 | 9.02 (5.88 - 13.85) | 9.01 (149.07) | 8.98 (5.85) | 3.17 (2.1) | < 0.001 |
| Pneumoperitoneum | 7 | 26507 | 1807 | 61708144 | 9.02 (4.29 - 18.95) | 9.02 (49.7) | 8.99 (4.28) | 3.17 (1.15) | < 0.001 |
| Cogwheel rigidity | 4 | 26510 | 1038 | 61708913 | 8.97 (3.36 - 23.95) | 8.97 (28.21) | 8.94 (3.35) | 3.16 (0.49) | < 0.001 |
| Accidental exposure to product by child | 31 | 26483 | 8241 | 61701710 | 8.76 (6.16 - 12.47) | 8.76 (212.18) | 8.73 (6.13) | 3.13 (2.3) | < 0.001 |
| Immobile | 16 | 26498 | 4282 | 61705669 | 8.7 (5.33 - 14.22) | 8.7 (108.59) | 8.67 (5.3) | 3.12 (1.88) | < 0.001 |
| Ketonuria | 3 | 26511 | 805 | 61709146 | 8.67 (2.79 - 26.96) | 8.67 (20.29) | 8.65 (2.78) | 3.11 (0.12) | 0.002 |
| Circadian rhythm sleep disorder | 3 | 26511 | 819 | 61709132 | 8.53 (2.74 - 26.49) | 8.53 (19.86) | 8.5 (2.73) | 3.09 (0.12) | 0.002 |
| Agitation | 258 | 26256 | 72924 | 61637027 | 8.31 (7.35 - 9.39) | 8.23 (1635.95) | 8.21 (7.26) | 3.04 (2.82) | < 0.001 |
| Polydipsia | 14 | 26500 | 3929 | 61706022 | 8.3 (4.91 - 14.02) | 8.29 (89.48) | 8.27 (4.89) | 3.05 (1.73) | < 0.001 |
| Incontinence | 37 | 26477 | 10461 | 61699490 | 8.24 (5.97 - 11.38) | 8.23 (234.29) | 8.21 (5.94) | 3.04 (2.32) | < 0.001 |
| Hyporesponsive to stimuli | 4 | 26510 | 1131 | 61708820 | 8.23 (3.08 - 21.97) | 8.23 (25.32) | 8.21 (3.07) | 3.04 (0.46) | < 0.001 |
| Myoclonic epilepsy | 5 | 26509 | 1455 | 61708496 | 8 (3.32 - 19.25) | 8 (30.51) | 7.97 (3.31) | 3 (0.7) | < 0.001 |
| Orthostatic hypotension | 59 | 26455 | 17252 | 61692699 | 7.98 (6.17 - 10.3) | 7.96 (357.91) | 7.94 (6.14) | 2.99 (2.46) | < 0.001 |
| Akinesia | 7 | 26507 | 2052 | 61707899 | 7.94 (3.78 - 16.68) | 7.94 (42.32) | 7.92 (3.77) | 2.98 (1.06) | < 0.001 |
| Psychomotor hyperactivity | 56 | 26458 | 16479 | 61693472 | 7.92 (6.09 - 10.3) | 7.91 (336.95) | 7.89 (6.06) | 2.98 (2.43) | < 0.001 |
| Urinary incontinence | 98 | 26416 | 29277 | 61680674 | 7.82 (6.41 - 9.53) | 7.79 (578.41) | 7.77 (6.37) | 2.96 (2.57) | < 0.001 |
| Depressed level of consciousness | 129 | 26385 | 39134 | 61670817 | 7.7 (6.48 - 9.16) | 7.67 (746.53) | 7.65 (6.43) | 2.94 (2.61) | < 0.001 |
| Unresponsive to stimuli | 84 | 26430 | 25765 | 61684186 | 7.61 (6.14 - 9.43) | 7.59 (479.1) | 7.57 (6.11) | 2.92 (2.5) | < 0.001 |
| Hyperglycaemic hyperosmolar nonketotic syndrome | 4 | 26510 | 1227 | 61708724 | 7.59 (2.84 - 20.25) | 7.59 (22.8) | 7.57 (2.83) | 2.92 (0.42) | < 0.001 |
| Drug level increased | 53 | 26461 | 16351 | 61693600 | 7.56 (5.77 - 9.9) | 7.54 (299.97) | 7.52 (5.74) | 2.91 (2.35) | < 0.001 |
| Rhabdomyolysis | 132 | 26382 | 41041 | 61668910 | 7.52 (6.33 - 8.92) | 7.49 (739.87) | 7.46 (6.29) | 2.9 (2.58) | < 0.001 |
| Hypopnoea | 11 | 26503 | 3412 | 61706539 | 7.51 (4.15 - 13.57) | 7.5 (61.81) | 7.48 (4.14) | 2.9 (1.45) | < 0.001 |
| Mallory-weiss syndrome | 4 | 26510 | 1265 | 61708686 | 7.36 (2.76 - 19.64) | 7.36 (21.91) | 7.34 (2.75) | 2.88 (0.4) | < 0.001 |
| Disinhibition | 5 | 26509 | 1586 | 61708365 | 7.34 (3.05 - 17.66) | 7.34 (27.28) | 7.32 (3.04) | 2.87 (0.65) | < 0.001 |
| Coma scale abnormal | 10 | 26504 | 3199 | 61706752 | 7.28 (3.91 - 13.54) | 7.28 (53.96) | 7.26 (3.9) | 2.86 (1.34) | < 0.001 |
| Sinus arrhythmia | 4 | 26510 | 1300 | 61708651 | 7.16 (2.68 - 19.11) | 7.16 (21.14) | 7.14 (2.68) | 2.84 (0.39) | < 0.001 |
| Ventricular tachycardia | 50 | 26464 | 16419 | 61693532 | 7.1 (5.38 - 9.37) | 7.09 (260.71) | 7.07 (5.35) | 2.82 (2.25) | < 0.001 |
| Sopor | 40 | 26474 | 13161 | 61696790 | 7.08 (5.19 - 9.66) | 7.07 (208.02) | 7.06 (5.17) | 2.82 (2.17) | < 0.001 |
| Consciousness fluctuating | 3 | 26511 | 999 | 61708952 | 6.99 (2.25 - 21.71) | 6.99 (15.35) | 6.97 (2.24) | 2.8 (0.04) | 0.003 |
| Hallucination | 220 | 26294 | 73852 | 61636099 | 6.98 (6.11 - 7.98) | 6.93 (1115.08) | 6.92 (6.06) | 2.79 (2.56) | < 0.001 |
| Impulsive behaviour | 11 | 26503 | 3707 | 61706244 | 6.91 (3.82 - 12.49) | 6.91 (55.4) | 6.89 (3.81) | 2.78 (1.37) | < 0.001 |
| Ileus paralytic | 13 | 26501 | 4407 | 61705544 | 6.87 (3.98 - 11.84) | 6.87 (64.96) | 6.85 (3.97) | 2.78 (1.5) | < 0.001 |
| Thrombocytopenic purpura | 4 | 26510 | 1355 | 61708596 | 6.87 (2.58 - 18.34) | 6.87 (20.01) | 6.85 (2.57) | 2.78 (0.37) | < 0.001 |
| Language disorder | 7 | 26507 | 2434 | 61707517 | 6.7 (3.19 - 14.06) | 6.69 (33.8) | 6.68 (3.18) | 2.74 (0.94) | < 0.001 |
| Acute psychosis | 7 | 26507 | 2436 | 61707515 | 6.69 (3.19 - 14.05) | 6.69 (33.77) | 6.67 (3.18) | 2.74 (0.94) | < 0.001 |
| Reduced facial expression | 4 | 26510 | 1394 | 61708557 | 6.68 (2.5 - 17.82) | 6.68 (19.26) | 6.66 (2.5) | 2.74 (0.35) | 0.001 |
| Unmasking of previously unidentified disease | 3 | 26511 | 1057 | 61708894 | 6.61 (2.13 - 20.52) | 6.61 (14.23) | 6.59 (2.12) | 2.72 (0.01) | 0.004 |
| Drug interaction | 434 | 26080 | 156532 | 61553419 | 6.54 (5.95 - 7.2) | 6.45 (1999.44) | 6.44 (5.85) | 2.69 (2.53) | < 0.001 |
| Aortic valve stenosis | 5 | 26509 | 1779 | 61708172 | 6.54 (2.72 - 15.74) | 6.54 (23.41) | 6.53 (2.71) | 2.71 (0.58) | < 0.001 |
| Disorganised speech | 5 | 26509 | 1783 | 61708168 | 6.53 (2.71 - 15.7) | 6.53 (23.34) | 6.51 (2.71) | 2.7 (0.58) | < 0.001 |
| Extrapyramidal disorder | 69 | 26445 | 24727 | 61685224 | 6.51 (5.14 - 8.25) | 6.49 (319.99) | 6.48 (5.11) | 2.7 (2.24) | < 0.001 |
| Petit mal epilepsy | 14 | 26500 | 5027 | 61704924 | 6.48 (3.84 - 10.96) | 6.48 (64.73) | 6.47 (3.83) | 2.69 (1.5) | < 0.001 |
| Duodenal ulcer perforation | 20 | 26494 | 7234 | 61702717 | 6.44 (4.15 - 9.99) | 6.43 (91.56) | 6.42 (4.14) | 2.68 (1.72) | < 0.001 |
| Hiccups | 22 | 26492 | 8036 | 61701915 | 6.38 (4.2 - 9.69) | 6.37 (99.37) | 6.36 (4.18) | 2.67 (1.76) | < 0.001 |
| Confusional state | 427 | 26087 | 159802 | 61550149 | 6.3 (5.73 - 6.94) | 6.22 (1870.07) | 6.21 (5.64) | 2.63 (2.48) | < 0.001 |
| Secondary adrenocortical insufficiency | 4 | 26510 | 1477 | 61708474 | 6.3 (2.36 - 16.82) | 6.3 (17.8) | 6.29 (2.36) | 2.65 (0.32) | 0.001 |
| Marasmus | 4 | 26510 | 1492 | 61708459 | 6.24 (2.34 - 16.65) | 6.24 (17.55) | 6.23 (2.33) | 2.64 (0.31) | 0.001 |
| Abnormal behaviour | 103 | 26411 | 39445 | 61670506 | 6.1 (5.02 - 7.4) | 6.08 (436.07) | 6.06 (5) | 2.6 (2.25) | < 0.001 |
| Oesophageal ulcer | 10 | 26504 | 3824 | 61706127 | 6.09 (3.27 - 11.33) | 6.09 (42.4) | 6.07 (3.26) | 2.6 (1.18) | < 0.001 |
| Hydrocephalus | 14 | 26500 | 5369 | 61704582 | 6.07 (3.59 - 10.26) | 6.07 (59.12) | 6.06 (3.58) | 2.6 (1.43) | < 0.001 |
| Bundle branch block left | 11 | 26503 | 4247 | 61705704 | 6.03 (3.34 - 10.9) | 6.03 (46.02) | 6.02 (3.33) | 2.59 (1.25) | < 0.001 |
| Clonus | 7 | 26507 | 2710 | 61707241 | 6.01 (2.86 - 12.63) | 6.01 (29.17) | 6 (2.86) | 2.58 (0.86) | < 0.001 |
| Colitis microscopic | 12 | 26502 | 4875 | 61705076 | 5.73 (3.25 - 10.1) | 5.73 (46.73) | 5.72 (3.24) | 2.52 (1.27) | < 0.001 |
| Haemodynamic instability | 18 | 26496 | 7420 | 61702531 | 5.65 (3.56 - 8.97) | 5.65 (68.66) | 5.63 (3.55) | 2.49 (1.52) | < 0.001 |
| Inhibitory drug interaction | 4 | 26510 | 1658 | 61708293 | 5.62 (2.11 - 14.98) | 5.62 (15.14) | 5.6 (2.1) | 2.49 (0.25) | 0.002 |
| Resuscitation | 4 | 26510 | 1669 | 61708282 | 5.58 (2.09 - 14.88) | 5.58 (14.99) | 5.57 (2.09) | 2.48 (0.25) | 0.002 |
| Dermatitis allergic | 29 | 26485 | 12305 | 61697646 | 5.49 (3.81 - 7.91) | 5.49 (106.13) | 5.47 (3.8) | 2.45 (1.72) | < 0.001 |
| Pemphigoid | 17 | 26497 | 7300 | 61702651 | 5.42 (3.37 - 8.73) | 5.42 (61.14) | 5.41 (3.36) | 2.44 (1.44) | < 0.001 |
| Emotional poverty | 4 | 26510 | 1728 | 61708223 | 5.39 (2.02 - 14.37) | 5.39 (14.26) | 5.38 (2.02) | 2.43 (0.23) | 0.002 |
| Euphoric mood | 24 | 26490 | 10570 | 61699381 | 5.29 (3.54 - 7.9) | 5.28 (83.2) | 5.27 (3.53) | 2.4 (1.59) | < 0.001 |
| Restlessness | 82 | 26432 | 36217 | 61673734 | 5.28 (4.25 - 6.56) | 5.27 (283.2) | 5.26 (4.23) | 2.4 (2.01) | < 0.001 |
| Respiratory rate increased | 20 | 26494 | 8837 | 61701114 | 5.27 (3.4 - 8.18) | 5.27 (69) | 5.26 (3.39) | 2.39 (1.5) | < 0.001 |
| Generalised tonic-clonic seizure | 57 | 26457 | 25479 | 61684472 | 5.22 (4.02 - 6.77) | 5.21 (193.38) | 5.2 (4.01) | 2.38 (1.9) | < 0.001 |
| Inappropriate antidiuretic hormone secretion | 22 | 26492 | 10051 | 61699900 | 5.1 (3.35 - 7.75) | 5.09 (72.25) | 5.09 (3.35) | 2.35 (1.51) | < 0.001 |
| Focal dyscognitive seizures | 5 | 26509 | 2286 | 61707665 | 5.09 (2.12 - 12.25) | 5.09 (16.4) | 5.08 (2.11) | 2.35 (0.42) | 0.001 |
| Partial seizures | 11 | 26503 | 5093 | 61704858 | 5.03 (2.78 - 9.09) | 5.03 (35.41) | 5.02 (2.78) | 2.33 (1.08) | < 0.001 |
| Volvulus | 5 | 26509 | 2345 | 61707606 | 4.96 (2.06 - 11.94) | 4.96 (15.79) | 4.95 (2.06) | 2.31 (0.4) | 0.001 |
| Cold sweat | 37 | 26477 | 17435 | 61692516 | 4.94 (3.58 - 6.83) | 4.94 (116.03) | 4.93 (3.57) | 2.3 (1.69) | < 0.001 |
| Upper gastrointestinal haemorrhage | 35 | 26479 | 16677 | 61693274 | 4.89 (3.51 - 6.81) | 4.88 (107.93) | 4.88 (3.5) | 2.29 (1.66) | < 0.001 |
| Abnormal loss of weight | 14 | 26500 | 6666 | 61703285 | 4.89 (2.89 - 8.26) | 4.89 (43.21) | 4.88 (2.89) | 2.29 (1.21) | < 0.001 |
| Hallucination, auditory | 33 | 26481 | 15741 | 61694210 | 4.88 (3.47 - 6.87) | 4.88 (101.59) | 4.87 (3.46) | 2.28 (1.63) | < 0.001 |
| Dysphemia | 9 | 26505 | 4332 | 61705619 | 4.84 (2.51 - 9.3) | 4.84 (27.33) | 4.83 (2.51) | 2.27 (0.89) | < 0.001 |
| Sedation | 53 | 26461 | 25755 | 61684196 | 4.8 (3.66 - 6.28) | 4.79 (158.65) | 4.78 (3.65) | 2.26 (1.77) | < 0.001 |
| Dyskinesia | 82 | 26432 | 40016 | 61669935 | 4.78 (3.85 - 5.94) | 4.77 (243.94) | 4.76 (3.83) | 2.25 (1.87) | < 0.001 |
| Hepatitis acute | 13 | 26501 | 6402 | 61703549 | 4.73 (2.74 - 8.15) | 4.73 (38.12) | 4.72 (2.74) | 2.24 (1.13) | < 0.001 |
| Gastric ulcer haemorrhage | 11 | 26503 | 5532 | 61704419 | 4.63 (2.56 - 8.37) | 4.63 (31.23) | 4.62 (2.56) | 2.21 (0.99) | < 0.001 |
| Epilepsy | 59 | 26455 | 30097 | 61679854 | 4.57 (3.54 - 5.9) | 4.56 (163.88) | 4.56 (3.53) | 2.19 (1.73) | < 0.001 |
| Personality change | 18 | 26496 | 9238 | 61700713 | 4.54 (2.86 - 7.21) | 4.53 (49.51) | 4.53 (2.85) | 2.18 (1.27) | < 0.001 |
| Gastritis erosive | 9 | 26505 | 4648 | 61705303 | 4.51 (2.34 - 8.67) | 4.51 (24.51) | 4.5 (2.34) | 2.17 (0.82) | < 0.001 |
| Lethargy | 109 | 26405 | 56798 | 61653153 | 4.48 (3.71 - 5.41) | 4.47 (292.97) | 4.46 (3.69) | 2.16 (1.84) | < 0.001 |
| Hallucinations, mixed | 8 | 26506 | 4154 | 61705797 | 4.48 (2.24 - 8.97) | 4.48 (21.6) | 4.48 (2.24) | 2.16 (0.73) | < 0.001 |
| Psychotic disorder | 56 | 26458 | 29207 | 61680744 | 4.47 (3.44 - 5.81) | 4.46 (150.23) | 4.46 (3.43) | 2.16 (1.69) | < 0.001 |
| Hypokalaemia | 87 | 26427 | 45469 | 61664482 | 4.46 (3.62 - 5.51) | 4.45 (232.7) | 4.45 (3.6) | 2.15 (1.79) | < 0.001 |
| Encephalopathy | 46 | 26468 | 24225 | 61685726 | 4.43 (3.31 - 5.91) | 4.42 (121.52) | 4.41 (3.3) | 2.14 (1.62) | < 0.001 |
| Screaming | 12 | 26502 | 6331 | 61703620 | 4.41 (2.5 - 7.78) | 4.41 (31.6) | 4.41 (2.5) | 2.14 (1) | < 0.001 |
| Energy increased | 11 | 26503 | 5854 | 61704097 | 4.37 (2.42 - 7.91) | 4.37 (28.57) | 4.37 (2.42) | 2.13 (0.94) | < 0.001 |
| Haematemesis | 47 | 26467 | 25120 | 61684831 | 4.36 (3.27 - 5.81) | 4.35 (121.29) | 4.35 (3.27) | 2.12 (1.61) | < 0.001 |
| Paranoia | 32 | 26482 | 17116 | 61692835 | 4.36 (3.08 - 6.16) | 4.35 (82.47) | 4.35 (3.07) | 2.12 (1.48) | < 0.001 |
| Eye movement disorder | 14 | 26500 | 7469 | 61702482 | 4.36 (2.58 - 7.37) | 4.36 (36.22) | 4.36 (2.58) | 2.12 (1.09) | < 0.001 |
| Incoherent | 12 | 26502 | 6434 | 61703517 | 4.34 (2.46 - 7.65) | 4.34 (30.8) | 4.33 (2.46) | 2.12 (0.99) | < 0.001 |
| Somnambulism | 13 | 26501 | 7077 | 61702874 | 4.28 (2.48 - 7.37) | 4.28 (32.56) | 4.27 (2.48) | 2.09 (1.02) | < 0.001 |
| Blood creatine increased | 8 | 26506 | 4397 | 61705554 | 4.24 (2.12 - 8.48) | 4.23 (19.73) | 4.23 (2.11) | 2.08 (0.67) | < 0.001 |
| Post-traumatic stress disorder | 12 | 26502 | 6637 | 61703314 | 4.21 (2.39 - 7.42) | 4.21 (29.3) | 4.2 (2.39) | 2.07 (0.95) | < 0.001 |
| Drug level above therapeutic | 8 | 26506 | 4477 | 61705474 | 4.16 (2.08 - 8.32) | 4.16 (19.16) | 4.15 (2.08) | 2.05 (0.66) | < 0.001 |
| Mental status changes | 49 | 26465 | 27576 | 61682375 | 4.14 (3.13 - 5.48) | 4.14 (116.34) | 4.13 (3.12) | 2.05 (1.55) | < 0.001 |
| Gastric ulcer | 33 | 26481 | 18841 | 61691110 | 4.08 (2.9 - 5.74) | 4.08 (76.51) | 4.07 (2.89) | 2.03 (1.4) | < 0.001 |
| Blood glucose fluctuation | 19 | 26495 | 10863 | 61699088 | 4.07 (2.6 - 6.39) | 4.07 (43.94) | 4.07 (2.59) | 2.02 (1.17) | < 0.001 |
| Bradykinesia | 10 | 26504 | 5747 | 61704204 | 4.05 (2.18 - 7.53) | 4.05 (22.93) | 4.04 (2.17) | 2.02 (0.79) | < 0.001 |
| Heart rate decreased | 60 | 26454 | 34834 | 61675117 | 4.02 (3.12 - 5.17) | 4.01 (135.35) | 4 (3.11) | 2 (1.56) | < 0.001 |
| Hypophagia | 48 | 26466 | 28048 | 61681903 | 3.99 (3 - 5.3) | 3.98 (107.1) | 3.98 (3) | 1.99 (1.49) | < 0.001 |
| Abnormal dreams | 47 | 26467 | 27431 | 61682520 | 3.99 (3 - 5.32) | 3.99 (105.08) | 3.98 (2.99) | 1.99 (1.49) | < 0.001 |
| Decreased interest | 10 | 26504 | 5830 | 61704121 | 3.99 (2.15 - 7.43) | 3.99 (22.39) | 3.99 (2.14) | 2 (0.78) | < 0.001 |
| Cardiac failure acute | 11 | 26503 | 6586 | 61703365 | 3.89 (2.15 - 7.03) | 3.89 (23.55) | 3.88 (2.15) | 1.96 (0.81) | < 0.001 |
| Neurotoxicity | 28 | 26486 | 17130 | 61692821 | 3.81 (2.63 - 5.52) | 3.8 (57.8) | 3.8 (2.62) | 1.93 (1.26) | < 0.001 |
| Shock haemorrhagic | 13 | 26501 | 8030 | 61701921 | 3.77 (2.19 - 6.5) | 3.77 (26.39) | 3.76 (2.18) | 1.91 (0.88) | < 0.001 |
| Loss of consciousness | 200 | 26314 | 126710 | 61583241 | 3.69 (3.21 - 4.25) | 3.67 (389.36) | 3.67 (3.19) | 1.88 (1.65) | < 0.001 |
| Extrasystoles | 11 | 26503 | 6945 | 61703006 | 3.69 (2.04 - 6.66) | 3.69 (21.5) | 3.68 (2.04) | 1.88 (0.76) | < 0.001 |
| Affect lability | 15 | 26499 | 9551 | 61700400 | 3.66 (2.2 - 6.07) | 3.66 (28.89) | 3.65 (2.2) | 1.87 (0.92) | < 0.001 |
| Lupus-like syndrome | 14 | 26500 | 8896 | 61701055 | 3.66 (2.17 - 6.19) | 3.66 (27.06) | 3.66 (2.17) | 1.87 (0.89) | < 0.001 |
| Decreased appetite | 369 | 26145 | 241550 | 61468401 | 3.59 (3.24 - 3.98) | 3.56 (679.39) | 3.55 (3.2) | 1.83 (1.67) | < 0.001 |
| Retinal detachment | 14 | 26500 | 9072 | 61700879 | 3.59 (2.13 - 6.07) | 3.59 (26.15) | 3.59 (2.12) | 1.84 (0.87) | < 0.001 |
| Body temperature decreased | 15 | 26499 | 9811 | 61700140 | 3.56 (2.14 - 5.91) | 3.56 (27.55) | 3.55 (2.14) | 1.83 (0.89) | < 0.001 |
| Fall | 482 | 26032 | 328487 | 61381464 | 3.46 (3.16 - 3.79) | 3.42 (826.43) | 3.41 (3.12) | 1.77 (1.63) | < 0.001 |
| Presyncope | 35 | 26479 | 23903 | 61686048 | 3.41 (2.45 - 4.75) | 3.41 (59.48) | 3.4 (2.44) | 1.77 (1.19) | < 0.001 |
| Dystonia | 28 | 26486 | 19213 | 61690738 | 3.39 (2.34 - 4.92) | 3.39 (47.17) | 3.39 (2.34) | 1.76 (1.11) | < 0.001 |
| Melaena | 31 | 26483 | 21834 | 61688117 | 3.31 (2.32 - 4.7) | 3.3 (49.77) | 3.3 (2.32) | 1.72 (1.11) | < 0.001 |
| Parkinson's disease | 27 | 26487 | 18972 | 61690979 | 3.31 (2.27 - 4.84) | 3.31 (43.54) | 3.31 (2.27) | 1.73 (1.07) | < 0.001 |
| Liver function test abnormal | 41 | 26473 | 28945 | 61681006 | 3.3 (2.43 - 4.48) | 3.3 (65.54) | 3.29 (2.42) | 1.72 (1.2) | < 0.001 |
| Cerebral infarction | 34 | 26480 | 24279 | 61685672 | 3.26 (2.33 - 4.57) | 3.26 (53.2) | 3.26 (2.33) | 1.7 (1.12) | < 0.001 |
| Seizure | 227 | 26287 | 170942 | 61539009 | 3.11 (2.73 - 3.54) | 3.09 (321.5) | 3.09 (2.71) | 1.63 (1.42) | < 0.001 |
| Speech disorder | 68 | 26446 | 51792 | 61658159 | 3.06 (2.41 - 3.88) | 3.06 (94) | 3.05 (2.41) | 1.61 (1.22) | < 0.001 |
| Muscle twitching | 29 | 26485 | 22833 | 61687118 | 2.96 (2.05 - 4.26) | 2.96 (37.5) | 2.95 (2.05) | 1.56 (0.94) | < 0.001 |
| Movement disorder | 40 | 26474 | 31835 | 61678116 | 2.93 (2.15 - 3.99) | 2.92 (50.62) | 2.92 (2.14) | 1.55 (1.03) | < 0.001 |
| Somnolence | 240 | 26274 | 198583 | 61511368 | 2.83 (2.49 - 3.21) | 2.81 (280.98) | 2.81 (2.48) | 1.49 (1.29) | < 0.001 |
| Arrhythmia | 55 | 26459 | 47028 | 61662923 | 2.73 (2.09 - 3.55) | 2.72 (59.89) | 2.72 (2.09) | 1.44 (1.01) | < 0.001 |
| Coma | 54 | 26460 | 47177 | 61662774 | 2.67 (2.04 - 3.48) | 2.66 (56.11) | 2.66 (2.04) | 1.41 (0.98) | < 0.001 |
| Cardiac arrest | 93 | 26421 | 82265 | 61627686 | 2.64 (2.15 - 3.23) | 2.63 (94.06) | 2.63 (2.14) | 1.39 (1.07) | < 0.001 |
| Tremor | 182 | 26332 | 164184 | 61545767 | 2.59 (2.24 - 3) | 2.58 (176.38) | 2.58 (2.23) | 1.37 (1.14) | < 0.001 |
| Vomiting | 508 | 26006 | 463156 | 61246795 | 2.58 (2.37 - 2.82) | 2.55 (482.92) | 2.55 (2.34) | 1.35 (1.22) | < 0.001 |
| Toxicity to various agents | 197 | 26317 | 182894 | 61527057 | 2.52 (2.19 - 2.9) | 2.51 (178.79) | 2.51 (2.18) | 1.33 (1.11) | < 0.001 |
| Atrial fibrillation | 103 | 26411 | 96628 | 61613323 | 2.49 (2.05 - 3.02) | 2.48 (91.1) | 2.48 (2.04) | 1.31 (1.01) | < 0.001 |
| Dehydration | 138 | 26376 | 132062 | 61577889 | 2.44 (2.06 - 2.88) | 2.43 (116.5) | 2.43 (2.06) | 1.28 (1.02) | < 0.001 |
| Hyperhidrosis | 131 | 26383 | 127424 | 61582527 | 2.4 (2.02 - 2.85) | 2.39 (106.31) | 2.39 (2.01) | 1.26 (0.99) | < 0.001 |
| Hypotension | 202 | 26312 | 198130 | 61511821 | 2.38 (2.08 - 2.74) | 2.37 (160.81) | 2.37 (2.06) | 1.25 (1.03) | < 0.001 |

Supplementary Table S11. Distribution of adverse events and signal detection results at the Preferred erm (PT) level for Galantamine.

| **PT** | **a** | **b** | **c** | **d** | **ROR(95%CI)** | **PRR(X^2^)** | **EBGM(EBGM05)** | **IC(IC025)** | **FDR** |
| --- | --- | --- | --- | --- | --- | --- | --- | --- | --- |
| Poriomania | 6 | 5220 | 186 | 61731053 | 381.48 (169.12 - 860.47) | 381.04 (2203.21) | 369.17 (163.67) | 8.53 (1.67) | < 0.001 |
| Pleurothotonus | 15 | 5211 | 887 | 61730352 | 200.33 (120.17 - 333.96) | 199.76 (2917.15) | 196.45 (117.84) | 7.62 (3.16) | < 0.001 |
| Electrocardiogram qrs complex shortened | 6 | 5220 | 375 | 61730864 | 189.21 (84.43 - 424.06) | 189 (1104.35) | 186.04 (83.01) | 7.54 (1.66) | < 0.001 |
| Cholinergic syndrome | 7 | 5219 | 625 | 61730614 | 132.47 (62.86 - 279.17) | 132.3 (902.05) | 130.84 (62.09) | 7.03 (1.9) | < 0.001 |
| Marasmus | 15 | 5211 | 1481 | 61729758 | 119.98 (72.09 - 199.67) | 119.64 (1747.05) | 118.45 (71.17) | 6.89 (3.1) | < 0.001 |
| Rhythm idioventricular | 3 | 5223 | 322 | 61730917 | 110.12 (35.32 - 343.34) | 110.05 (321.19) | 109.05 (34.97) | 6.77 (0.51) | < 0.001 |
| Oesophageal achalasia | 5 | 5221 | 630 | 61730609 | 93.84 (38.91 - 226.33) | 93.75 (455.19) | 93.02 (38.57) | 6.54 (1.33) | < 0.001 |
| Flashback | 4 | 5222 | 567 | 61730672 | 83.4 (31.18 - 223.05) | 83.33 (323.1) | 82.76 (30.94) | 6.37 (0.96) | < 0.001 |
| Vasculitic rash | 5 | 5221 | 765 | 61730474 | 77.28 (32.06 - 186.27) | 77.2 (373.65) | 76.71 (31.82) | 6.26 (1.31) | < 0.001 |
| Sedation complication | 15 | 5211 | 2456 | 61728783 | 72.35 (43.52 - 120.28) | 72.14 (1046.02) | 71.71 (43.13) | 6.16 (3) | < 0.001 |
| Diet refusal | 3 | 5223 | 727 | 61730512 | 48.77 (15.69 - 151.63) | 48.74 (139.72) | 48.55 (15.62) | 5.6 (0.47) | < 0.001 |
| Sinus arrest | 6 | 5220 | 1525 | 61729714 | 46.53 (20.86 - 103.78) | 46.47 (265.94) | 46.3 (20.76) | 5.53 (1.54) | < 0.001 |
| Creatinine renal clearance decreased | 13 | 5213 | 3985 | 61727254 | 38.63 (22.39 - 66.63) | 38.53 (473.77) | 38.41 (22.27) | 5.26 (2.61) | < 0.001 |
| Compartment syndrome | 7 | 5219 | 2570 | 61728669 | 32.22 (15.33 - 67.68) | 32.17 (210.87) | 32.09 (15.27) | 5 (1.69) | < 0.001 |
| Bradyarrhythmia | 4 | 5222 | 1627 | 61729612 | 29.06 (10.89 - 77.56) | 29.04 (108.04) | 28.97 (10.86) | 4.86 (0.84) | < 0.001 |
| Haemorrhage subcutaneous | 8 | 5218 | 3511 | 61727728 | 26.95 (13.46 - 53.97) | 26.92 (199.17) | 26.86 (13.41) | 4.75 (1.83) | < 0.001 |
| Atrioventricular block complete | 15 | 5211 | 6714 | 61724525 | 26.46 (15.93 - 43.95) | 26.39 (365.65) | 26.33 (15.86) | 4.72 (2.63) | < 0.001 |
| Hepatitis toxic | 5 | 5221 | 2593 | 61728646 | 22.8 (9.48 - 54.84) | 22.78 (103.91) | 22.74 (9.45) | 4.51 (1.12) | < 0.001 |
| Atrioventricular block second degree | 5 | 5221 | 3017 | 61728222 | 19.59 (8.15 - 47.13) | 19.58 (88) | 19.55 (8.13) | 4.29 (1.08) | < 0.001 |
| Conduction disorder | 3 | 5223 | 1818 | 61729421 | 19.5 (6.28 - 60.55) | 19.49 (52.55) | 19.46 (6.27) | 4.28 (0.35) | < 0.001 |
| Increased bronchial secretion | 4 | 5222 | 2534 | 61728705 | 18.66 (7 - 49.77) | 18.65 (66.7) | 18.62 (6.98) | 4.22 (0.75) | < 0.001 |
| Gastric perforation | 4 | 5222 | 2678 | 61728561 | 17.66 (6.62 - 47.1) | 17.64 (62.71) | 17.62 (6.61) | 4.14 (0.73) | < 0.001 |
| Atrioventricular block | 11 | 5215 | 7642 | 61723597 | 17.04 (9.42 - 30.8) | 17 (165.46) | 16.98 (9.39) | 4.09 (2.03) | < 0.001 |
| Bundle branch block left | 6 | 5220 | 4252 | 61726987 | 16.69 (7.49 - 37.18) | 16.67 (88.25) | 16.65 (7.47) | 4.06 (1.27) | < 0.001 |
| Miosis | 11 | 5215 | 7869 | 61723370 | 16.55 (9.15 - 29.91) | 16.51 (160.1) | 16.49 (9.12) | 4.04 (2.01) | < 0.001 |
| Sinus node dysfunction | 4 | 5222 | 3002 | 61728237 | 15.75 (5.91 - 42.01) | 15.74 (55.14) | 15.72 (5.89) | 3.97 (0.7) | < 0.001 |
| Behaviour disorder | 7 | 5219 | 5436 | 61725803 | 15.23 (7.25 - 31.98) | 15.21 (92.82) | 15.19 (7.24) | 3.93 (1.43) | < 0.001 |
| Parkinsonism | 12 | 5214 | 9622 | 61721617 | 14.76 (8.38 - 26.02) | 14.73 (153.43) | 14.71 (8.35) | 3.88 (2.04) | < 0.001 |
| Persecutory delusion | 3 | 5223 | 2437 | 61728802 | 14.55 (4.69 - 45.16) | 14.54 (37.78) | 14.52 (4.68) | 3.86 (0.28) | < 0.001 |
| Physical deconditioning | 3 | 5223 | 2469 | 61728770 | 14.36 (4.63 - 44.57) | 14.35 (37.22) | 14.34 (4.62) | 3.84 (0.28) | < 0.001 |
| Tachyarrhythmia | 3 | 5223 | 2472 | 61728767 | 14.34 (4.62 - 44.52) | 14.34 (37.17) | 14.32 (4.61) | 3.84 (0.28) | < 0.001 |
| Fracture | 22 | 5204 | 18641 | 61712598 | 14 (9.2 - 21.28) | 13.94 (264.04) | 13.93 (9.16) | 3.8 (2.55) | < 0.001 |
| Bradycardia | 61 | 5165 | 52616 | 61678623 | 13.84 (10.75 - 17.82) | 13.69 (717.6) | 13.68 (10.63) | 3.77 (3.14) | < 0.001 |
| Torsade de pointes | 9 | 5217 | 7773 | 61723466 | 13.7 (7.12 - 26.35) | 13.68 (105.64) | 13.66 (7.1) | 3.77 (1.68) | < 0.001 |
| Atrioventricular block first degree | 5 | 5221 | 4346 | 61726893 | 13.6 (5.66 - 32.71) | 13.59 (58.25) | 13.58 (5.65) | 3.76 (0.95) | < 0.001 |
| Myoclonus | 13 | 5213 | 11794 | 61719445 | 13.05 (7.57 - 22.5) | 13.02 (144.13) | 13.01 (7.55) | 3.7 (2.03) | < 0.001 |
| Pemphigoid | 8 | 5218 | 7309 | 61723930 | 12.95 (6.47 - 25.91) | 12.93 (87.97) | 12.92 (6.45) | 3.69 (1.51) | < 0.001 |
| Gastric ulcer haemorrhage | 6 | 5220 | 5537 | 61725702 | 12.81 (5.75 - 28.55) | 12.8 (65.2) | 12.79 (5.74) | 3.68 (1.16) | < 0.001 |
| Oesophageal stenosis | 3 | 5223 | 2818 | 61728421 | 12.58 (4.05 - 39.05) | 12.58 (31.93) | 12.56 (4.05) | 3.65 (0.25) | < 0.001 |
| Delirium | 36 | 5190 | 34199 | 61697040 | 12.51 (9.01 - 17.37) | 12.43 (378.35) | 12.42 (8.95) | 3.63 (2.77) | < 0.001 |
| Electrocardiogram qt prolonged | 38 | 5188 | 36191 | 61695048 | 12.49 (9.07 - 17.18) | 12.4 (398.18) | 12.39 (9) | 3.63 (2.8) | < 0.001 |
| Communication disorder | 4 | 5222 | 3801 | 61727438 | 12.44 (4.66 - 33.17) | 12.43 (42) | 12.42 (4.66) | 3.63 (0.63) | < 0.001 |
| Acute generalised exanthematous pustulosis | 8 | 5218 | 7634 | 61723605 | 12.4 (6.19 - 24.81) | 12.38 (83.6) | 12.37 (6.18) | 3.63 (1.49) | < 0.001 |
| Dyspnoea at rest | 3 | 5223 | 2861 | 61728378 | 12.39 (3.99 - 38.46) | 12.39 (31.37) | 12.37 (3.99) | 3.63 (0.24) | < 0.001 |
| Salivary hypersecretion | 10 | 5216 | 9876 | 61721363 | 11.98 (6.44 - 22.29) | 11.96 (100.36) | 11.95 (6.42) | 3.58 (1.71) | < 0.001 |
| Circulatory collapse | 17 | 5209 | 16947 | 61714292 | 11.88 (7.38 - 19.14) | 11.85 (168.75) | 11.84 (7.35) | 3.57 (2.2) | < 0.001 |
| Hypothermia | 10 | 5216 | 10020 | 61721219 | 11.81 (6.35 - 21.97) | 11.79 (98.65) | 11.78 (6.33) | 3.56 (1.7) | < 0.001 |
| Cerebrovascular disorder | 3 | 5223 | 3019 | 61728220 | 11.74 (3.78 - 36.45) | 11.74 (29.44) | 11.73 (3.78) | 3.55 (0.23) | < 0.001 |
| Tearfulness | 4 | 5222 | 4280 | 61726959 | 11.05 (4.14 - 29.46) | 11.04 (36.49) | 11.03 (4.14) | 3.46 (0.58) | < 0.001 |
| Mania | 15 | 5211 | 16248 | 61714991 | 10.93 (6.59 - 18.15) | 10.91 (134.86) | 10.9 (6.56) | 3.45 (2.03) | < 0.001 |
| Ileus paralytic | 4 | 5222 | 4416 | 61726823 | 10.71 (4.02 - 28.55) | 10.7 (35.14) | 10.69 (4.01) | 3.42 (0.57) | < 0.001 |
| Post-traumatic stress disorder | 6 | 5220 | 6643 | 61724596 | 10.68 (4.79 - 23.79) | 10.67 (52.53) | 10.66 (4.79) | 3.41 (1.07) | < 0.001 |
| Altered state of consciousness | 19 | 5207 | 21328 | 61709911 | 10.56 (6.73 - 16.57) | 10.52 (163.65) | 10.51 (6.7) | 3.39 (2.19) | < 0.001 |
| Blood calcium decreased | 10 | 5216 | 11222 | 61720017 | 10.54 (5.67 - 19.61) | 10.53 (86.15) | 10.52 (5.65) | 3.39 (1.62) | < 0.001 |
| Subarachnoid haemorrhage | 9 | 5217 | 10122 | 61721117 | 10.52 (5.47 - 20.23) | 10.5 (77.33) | 10.49 (5.46) | 3.39 (1.51) | < 0.001 |
| Renal function test abnormal | 4 | 5222 | 4552 | 61726687 | 10.39 (3.9 - 27.7) | 10.38 (33.88) | 10.37 (3.89) | 3.37 (0.56) | < 0.001 |
| Orthostatic hypotension | 15 | 5211 | 17296 | 61713943 | 10.27 (6.19 - 17.05) | 10.24 (125.05) | 10.24 (6.17) | 3.36 (1.98) | < 0.001 |
| Sinus bradycardia | 8 | 5218 | 9633 | 61721606 | 9.82 (4.91 - 19.66) | 9.81 (63.25) | 9.8 (4.9) | 3.29 (1.35) | < 0.001 |
| Arrhythmia | 38 | 5188 | 47045 | 61684194 | 9.6 (6.98 - 13.22) | 9.54 (290.54) | 9.53 (6.93) | 3.25 (2.5) | < 0.001 |
| Self-destructive behaviour | 3 | 5223 | 3801 | 61727438 | 9.33 (3.01 - 28.94) | 9.32 (22.27) | 9.32 (3) | 3.22 (0.15) | < 0.001 |
| Delusion | 12 | 5214 | 15345 | 61715894 | 9.26 (5.25 - 16.31) | 9.24 (88.1) | 9.23 (5.24) | 3.21 (1.7) | < 0.001 |
| Activated partial thromboplastin time prolonged | 4 | 5222 | 5115 | 61726124 | 9.24 (3.47 - 24.65) | 9.24 (29.36) | 9.23 (3.46) | 3.21 (0.51) | < 0.001 |
| Hallucination, visual | 16 | 5210 | 20753 | 61710486 | 9.13 (5.59 - 14.92) | 9.11 (115.42) | 9.1 (5.57) | 3.19 (1.92) | < 0.001 |
| Depressed level of consciousness | 30 | 5196 | 39233 | 61692006 | 9.08 (6.34 - 13) | 9.03 (214.27) | 9.03 (6.3) | 3.17 (2.32) | < 0.001 |
| Neuroleptic malignant syndrome | 9 | 5217 | 11750 | 61719489 | 9.06 (4.71 - 17.43) | 9.05 (64.39) | 9.04 (4.7) | 3.18 (1.41) | < 0.001 |
| Cerebral infarction | 18 | 5208 | 24295 | 61706944 | 8.78 (5.53 - 13.95) | 8.75 (123.54) | 8.75 (5.5) | 3.13 (1.97) | < 0.001 |
| Affect lability | 7 | 5219 | 9559 | 61721680 | 8.66 (4.13 - 18.18) | 8.65 (47.33) | 8.64 (4.12) | 3.11 (1.12) | < 0.001 |
| Sedation | 18 | 5208 | 25790 | 61705449 | 8.27 (5.21 - 13.14) | 8.24 (114.55) | 8.24 (5.19) | 3.04 (1.91) | < 0.001 |
| Prothrombin time prolonged | 4 | 5222 | 6085 | 61725154 | 7.77 (2.91 - 20.72) | 7.76 (23.56) | 7.76 (2.91) | 2.96 (0.43) | < 0.001 |
| Bundle branch block right | 3 | 5223 | 4621 | 61726618 | 7.67 (2.47 - 23.81) | 7.67 (17.39) | 7.66 (2.47) | 2.94 (0.08) | 0.002 |
| Hepatitis acute | 4 | 5222 | 6411 | 61724828 | 7.37 (2.77 - 19.66) | 7.37 (22.01) | 7.37 (2.76) | 2.88 (0.4) | < 0.001 |
| Product use complaint | 10 | 5216 | 16303 | 61714936 | 7.26 (3.9 - 13.5) | 7.25 (53.82) | 7.24 (3.89) | 2.86 (1.34) | < 0.001 |
| Hypernatraemia | 3 | 5223 | 4920 | 61726319 | 7.21 (2.32 - 22.36) | 7.2 (16.02) | 7.2 (2.32) | 2.85 (0.05) | 0.002 |
| Abdominal tenderness | 3 | 5223 | 4930 | 61726309 | 7.19 (2.32 - 22.31) | 7.19 (15.97) | 7.18 (2.32) | 2.84 (0.05) | 0.002 |
| Aggression | 30 | 5196 | 49609 | 61681630 | 7.18 (5.01 - 10.28) | 7.14 (158.53) | 7.14 (4.99) | 2.84 (2.05) | < 0.001 |
| Meningitis | 4 | 5222 | 6602 | 61724637 | 7.16 (2.69 - 19.09) | 7.16 (21.18) | 7.15 (2.68) | 2.84 (0.39) | < 0.001 |
| Hemiplegia | 5 | 5221 | 8298 | 61722941 | 7.12 (2.96 - 17.13) | 7.12 (26.28) | 7.11 (2.96) | 2.83 (0.64) | < 0.001 |
| Toxic skin eruption | 6 | 5220 | 10011 | 61721228 | 7.09 (3.18 - 15.79) | 7.08 (31.31) | 7.08 (3.18) | 2.82 (0.83) | < 0.001 |
| Abnormal loss of weight | 4 | 5222 | 6676 | 61724563 | 7.08 (2.66 - 18.88) | 7.08 (20.86) | 7.07 (2.65) | 2.82 (0.38) | < 0.001 |
| Drooling | 4 | 5222 | 6801 | 61724438 | 6.95 (2.61 - 18.54) | 6.95 (20.36) | 6.94 (2.6) | 2.8 (0.37) | < 0.001 |
| Femoral neck fracture | 3 | 5223 | 5107 | 61726132 | 6.94 (2.24 - 21.54) | 6.94 (15.24) | 6.94 (2.24) | 2.79 (0.04) | 0.002 |
| Syncope | 54 | 5172 | 99917 | 61631322 | 6.44 (4.93 - 8.42) | 6.38 (245.46) | 6.38 (4.88) | 2.67 (2.15) | < 0.001 |
| Psychomotor hyperactivity | 9 | 5217 | 16526 | 61714713 | 6.44 (3.35 - 12.39) | 6.43 (41.28) | 6.43 (3.34) | 2.68 (1.15) | < 0.001 |
| Rhabdomyolysis | 22 | 5204 | 41151 | 61690088 | 6.34 (4.17 - 9.63) | 6.32 (98.43) | 6.31 (4.15) | 2.66 (1.75) | < 0.001 |
| Aphasia | 16 | 5210 | 30425 | 61700814 | 6.23 (3.81 - 10.17) | 6.21 (69.96) | 6.21 (3.8) | 2.63 (1.55) | < 0.001 |
| Nightmare | 18 | 5208 | 34345 | 61696894 | 6.21 (3.91 - 9.86) | 6.19 (78.34) | 6.19 (3.9) | 2.63 (1.62) | < 0.001 |
| Urinary incontinence | 15 | 5211 | 29360 | 61701879 | 6.05 (3.64 - 10.04) | 6.03 (63.01) | 6.03 (3.63) | 2.59 (1.48) | < 0.001 |
| Agitation | 37 | 5189 | 73145 | 61658094 | 6.01 (4.35 - 8.31) | 5.98 (153.38) | 5.97 (4.32) | 2.58 (1.93) | < 0.001 |
| Sinus tachycardia | 7 | 5219 | 14109 | 61717130 | 5.87 (2.8 - 12.32) | 5.86 (28.21) | 5.86 (2.79) | 2.55 (0.84) | < 0.001 |
| Inappropriate antidiuretic hormone secretion | 5 | 5221 | 10068 | 61721171 | 5.87 (2.44 - 14.11) | 5.87 (20.18) | 5.86 (2.44) | 2.55 (0.52) | < 0.001 |
| Ventricular tachycardia | 8 | 5218 | 16461 | 61714778 | 5.75 (2.87 - 11.5) | 5.74 (31.31) | 5.74 (2.87) | 2.52 (0.95) | < 0.001 |
| Cerebral haemorrhage | 17 | 5209 | 35405 | 61695834 | 5.69 (3.53 - 9.16) | 5.67 (65.42) | 5.67 (3.52) | 2.5 (1.49) | < 0.001 |
| Epilepsy | 14 | 5212 | 30142 | 61701097 | 5.5 (3.25 - 9.29) | 5.49 (51.36) | 5.48 (3.25) | 2.46 (1.33) | < 0.001 |
| Feeding disorder | 9 | 5217 | 19675 | 61711564 | 5.41 (2.81 - 10.41) | 5.4 (32.29) | 5.4 (2.81) | 2.43 (0.99) | < 0.001 |
| Loss of consciousness | 57 | 5169 | 126853 | 61604386 | 5.36 (4.12 - 6.95) | 5.31 (199.6) | 5.31 (4.09) | 2.41 (1.92) | < 0.001 |
| Hypokalaemia | 20 | 5206 | 45536 | 61685703 | 5.2 (3.35 - 8.07) | 5.19 (67.64) | 5.19 (3.34) | 2.37 (1.48) | < 0.001 |
| Renal impairment | 36 | 5190 | 82689 | 61648550 | 5.17 (3.73 - 7.18) | 5.14 (120.25) | 5.14 (3.7) | 2.36 (1.73) | < 0.001 |
| Abnormal behaviour | 17 | 5209 | 39531 | 61691708 | 5.09 (3.16 - 8.2) | 5.08 (55.71) | 5.08 (3.15) | 2.34 (1.37) | < 0.001 |
| Gastric ulcer | 8 | 5218 | 18866 | 61712373 | 5.02 (2.51 - 10.04) | 5.01 (25.67) | 5.01 (2.5) | 2.32 (0.83) | < 0.001 |
| Muscle rigidity | 5 | 5221 | 11819 | 61719420 | 5 (2.08 - 12.02) | 5 (15.98) | 5 (2.08) | 2.32 (0.41) | < 0.001 |
| Parkinson's disease | 8 | 5218 | 18991 | 61712248 | 4.98 (2.49 - 9.97) | 4.98 (25.41) | 4.97 (2.49) | 2.31 (0.82) | < 0.001 |
| Hallucination | 31 | 5195 | 74041 | 61657198 | 4.97 (3.49 - 7.07) | 4.95 (97.66) | 4.94 (3.47) | 2.31 (1.63) | < 0.001 |
| Blood creatine phosphokinase increased | 12 | 5214 | 29438 | 61701801 | 4.82 (2.74 - 8.5) | 4.82 (36.28) | 4.81 (2.73) | 2.27 (1.09) | < 0.001 |
| Cardiac failure | 32 | 5194 | 79619 | 61651620 | 4.77 (3.37 - 6.75) | 4.75 (94.75) | 4.75 (3.35) | 2.25 (1.59) | < 0.001 |
| Extrapyramidal disorder | 10 | 5216 | 24786 | 61706453 | 4.77 (2.57 - 8.88) | 4.77 (29.76) | 4.76 (2.56) | 2.25 (0.96) | < 0.001 |
| Dyskinesia | 16 | 5210 | 40082 | 61691157 | 4.73 (2.89 - 7.72) | 4.72 (46.85) | 4.71 (2.89) | 2.24 (1.25) | < 0.001 |
| Dysphagia | 39 | 5187 | 98166 | 61633073 | 4.72 (3.44 - 6.47) | 4.69 (113.47) | 4.69 (3.42) | 2.23 (1.64) | < 0.001 |
| Restlessness | 14 | 5212 | 36285 | 61694954 | 4.57 (2.7 - 7.72) | 4.56 (38.89) | 4.56 (2.7) | 2.19 (1.13) | < 0.001 |
| Urinary retention | 12 | 5214 | 33045 | 61698194 | 4.3 (2.44 - 7.57) | 4.29 (30.28) | 4.29 (2.43) | 2.1 (0.97) | < 0.001 |
| Stevens-johnson syndrome | 8 | 5218 | 22016 | 61709223 | 4.3 (2.15 - 8.6) | 4.29 (20.2) | 4.29 (2.14) | 2.1 (0.69) | < 0.001 |
| Decreased appetite | 86 | 5140 | 241833 | 61489406 | 4.25 (3.44 - 5.26) | 4.2 (210.48) | 4.2 (3.39) | 2.07 (1.71) | < 0.001 |
| Anger | 12 | 5214 | 33585 | 61697654 | 4.23 (2.4 - 7.45) | 4.22 (29.5) | 4.22 (2.39) | 2.08 (0.96) | < 0.001 |
| Fall | 106 | 5120 | 328863 | 61402376 | 3.87 (3.19 - 4.69) | 3.81 (220.53) | 3.81 (3.14) | 1.93 (1.61) | < 0.001 |
| Confusional state | 52 | 5174 | 160177 | 61571062 | 3.86 (2.94 - 5.08) | 3.83 (109.22) | 3.83 (2.92) | 1.94 (1.46) | < 0.001 |
| Interstitial lung disease | 15 | 5211 | 47771 | 61683468 | 3.72 (2.24 - 6.17) | 3.71 (29.69) | 3.71 (2.23) | 1.89 (0.94) | < 0.001 |
| Hyponatraemia | 17 | 5209 | 55652 | 61675587 | 3.62 (2.25 - 5.82) | 3.61 (32.07) | 3.61 (2.24) | 1.85 (0.97) | < 0.001 |
| Seizure | 43 | 5183 | 171126 | 61560113 | 2.98 (2.21 - 4.03) | 2.97 (56.26) | 2.97 (2.2) | 1.57 (1.07) | < 0.001 |
| Drug interaction | 39 | 5187 | 156927 | 61574312 | 2.95 (2.15 - 4.04) | 2.94 (49.89) | 2.94 (2.14) | 1.55 (1.03) | < 0.001 |
| Somnolence | 47 | 5179 | 198776 | 61532463 | 2.81 (2.11 - 3.74) | 2.79 (54.26) | 2.79 (2.1) | 1.48 (1.01) | < 0.001 |

Supplementary Table S12. Distribution of adverse events and signal detection results at the Preferred erm (PT) level for Lecanemab.

| **PT** | **a** | **b** | **c** | **d** | **ROR(95%CI)** | **PRR(X^2^)** | **EBGM(EBGM05)** | **IC(IC025)** | **FDR** |
| --- | --- | --- | --- | --- | --- | --- | --- | --- | --- |
| ARIA-H | 453 | 6938 | 485 | 61728589 | 8310.14 (7295.56 - 9465.83) | 7800.87 (1826734.25) | 4033.98 (3541.47) | 11.98 (8.5) | < 0.001 |
| ARIA-E | 488 | 6903 | 598 | 61728476 | 7297.37 (6460.08 - 8243.18) | 6815.62 (1830950.35) | 3753.43 (3322.77) | 11.87 (8.6) | < 0.001 |
| Superficial siderosis of central nervous system | 46 | 7345 | 69 | 61729005 | 5602.81 (3855.45 - 8142.11) | 5567.95 (153620.5) | 3341.17 (2299.15) | 11.71 (5.03) | < 0.001 |
| ARIA | 92 | 7299 | 180 | 61728894 | 4322.55 (3358.88 - 5562.72) | 4268.76 (259771.63) | 2825.25 (2195.39) | 11.46 (6.14) | < 0.001 |
| Cerebral microhaemorrhage | 27 | 7364 | 384 | 61728690 | 589.39 (398.69 - 871.31) | 587.24 (14763.69) | 548.73 (371.19) | 9.1 (4.17) | < 0.001 |
| Traumatic subarachnoid haemorrhage | 3 | 7388 | 54 | 61729020 | 464.18 (145.1 - 1484.92) | 464 (1313.05) | 439.63 (137.43) | 8.78 (0.5) | < 0.001 |
| Lacunar stroke | 3 | 7388 | 308 | 61728766 | 81.38 (26.1 - 253.79) | 81.35 (235.79) | 80.57 (25.84) | 6.33 (0.49) | < 0.001 |
| Lacunar infarction | 19 | 7372 | 2410 | 61726664 | 66.01 (42.01 - 103.74) | 65.85 (1203.9) | 65.34 (41.58) | 6.03 (3.3) | < 0.001 |
| Cerebellar infarction | 13 | 7378 | 1663 | 61727411 | 65.4 (37.88 - 112.93) | 65.29 (816.59) | 64.79 (37.52) | 6.02 (2.77) | < 0.001 |
| Hypertensive urgency | 3 | 7388 | 505 | 61728569 | 49.64 (15.95 - 154.45) | 49.62 (142.06) | 49.33 (15.85) | 5.62 (0.47) | < 0.001 |
| Cerebral mass effect | 5 | 7386 | 879 | 61728195 | 47.54 (19.73 - 114.54) | 47.51 (226.36) | 47.25 (19.61) | 5.56 (1.26) | < 0.001 |
| Infusion related reaction | 316 | 7075 | 64959 | 61664115 | 42.4 (37.87 - 47.47) | 40.63 (12168.22) | 40.44 (36.12) | 5.34 (5) | < 0.001 |
| Vasogenic cerebral oedema | 3 | 7388 | 697 | 61728377 | 35.96 (11.57 - 111.8) | 35.95 (101.49) | 35.8 (11.51) | 5.16 (0.44) | < 0.001 |
| Magnetic resonance imaging head abnormal | 9 | 7382 | 2286 | 61726788 | 32.92 (17.1 - 63.38) | 32.88 (277.13) | 32.76 (17.01) | 5.03 (2.06) | < 0.001 |
| Neuropsychological symptoms | 6 | 7385 | 1527 | 61727547 | 32.84 (14.73 - 73.24) | 32.82 (184.36) | 32.69 (14.66) | 5.03 (1.47) | < 0.001 |
| Basal ganglia haemorrhage | 3 | 7388 | 808 | 61728266 | 31.02 (9.98 - 96.41) | 31.01 (86.8) | 30.9 (9.94) | 4.95 (0.42) | < 0.001 |
| Cerebral haemorrhage | 117 | 7274 | 35305 | 61693769 | 28.11 (23.41 - 33.75) | 27.68 (3000.35) | 27.59 (22.98) | 4.79 (4.22) | < 0.001 |
| Magnetic resonance imaging abnormal | 15 | 7376 | 4481 | 61724593 | 28.01 (16.86 - 46.53) | 27.96 (388.63) | 27.87 (16.78) | 4.8 (2.66) | < 0.001 |
| Cerebral infarction | 78 | 7313 | 24235 | 61704839 | 27.16 (21.72 - 33.96) | 26.88 (1938.11) | 26.8 (21.43) | 4.74 (4.01) | < 0.001 |
| Drowning | 3 | 7388 | 990 | 61728084 | 25.32 (8.15 - 78.66) | 25.31 (69.83) | 25.24 (8.12) | 4.66 (0.39) | < 0.001 |
| Brain oedema | 30 | 7361 | 12517 | 61716557 | 20.09 (14.03 - 28.77) | 20.02 (540.83) | 19.97 (13.95) | 4.32 (3.11) | < 0.001 |
| Extradural haematoma | 3 | 7388 | 1319 | 61727755 | 19 (6.12 - 59.01) | 19 (51.03) | 18.96 (6.1) | 4.24 (0.34) | < 0.001 |
| Chills | 243 | 7148 | 115830 | 61613244 | 18.08 (15.91 - 20.55) | 17.52 (3784.79) | 17.49 (15.39) | 4.13 (3.85) | < 0.001 |
| Subdural haematoma | 28 | 7363 | 14217 | 61714857 | 16.51 (11.39 - 23.93) | 16.45 (405.57) | 16.42 (11.32) | 4.04 (2.88) | < 0.001 |
| Subdural haemorrhage | 5 | 7386 | 2731 | 61726343 | 15.3 (6.36 - 36.8) | 15.29 (66.66) | 15.26 (6.35) | 3.93 (1) | < 0.001 |
| Aortic dissection | 7 | 7384 | 4126 | 61724948 | 14.18 (6.75 - 29.78) | 14.17 (85.54) | 14.15 (6.74) | 3.82 (1.4) | < 0.001 |
| Intraventricular haemorrhage | 4 | 7387 | 2473 | 61726601 | 13.52 (5.07 - 36.05) | 13.51 (46.26) | 13.49 (5.06) | 3.75 (0.65) | < 0.001 |
| Femoral neck fracture | 8 | 7383 | 5102 | 61723972 | 13.11 (6.55 - 26.24) | 13.1 (89.25) | 13.08 (6.53) | 3.71 (1.52) | < 0.001 |
| Feeling cold | 42 | 7349 | 27542 | 61701532 | 12.8 (9.45 - 17.34) | 12.74 (453.73) | 12.72 (9.39) | 3.67 (2.88) | < 0.001 |
| Infusion site extravasation | 9 | 7382 | 6137 | 61722937 | 12.26 (6.37 - 23.59) | 12.25 (92.84) | 12.23 (6.36) | 3.61 (1.61) | < 0.001 |
| Infusion site bruising | 3 | 7388 | 2096 | 61726978 | 11.96 (3.85 - 37.12) | 11.95 (30.07) | 11.94 (3.85) | 3.58 (0.23) | < 0.001 |
| Fibrin d dimer increased | 5 | 7386 | 3540 | 61725534 | 11.8 (4.91 - 28.39) | 11.8 (49.34) | 11.78 (4.9) | 3.56 (0.89) | < 0.001 |
| Brain fog | 19 | 7372 | 14380 | 61714694 | 11.06 (7.05 - 17.36) | 11.04 (173.2) | 11.02 (7.02) | 3.46 (2.23) | < 0.001 |
| Slow speech | 3 | 7388 | 2370 | 61726704 | 10.58 (3.41 - 32.82) | 10.57 (25.97) | 10.56 (3.4) | 3.4 (0.19) | < 0.001 |
| Cervical vertebral fracture | 4 | 7387 | 3399 | 61725675 | 9.83 (3.69 - 26.22) | 9.83 (31.69) | 9.82 (3.68) | 3.3 (0.54) | < 0.001 |
| Clumsiness | 3 | 7388 | 2714 | 61726360 | 9.24 (2.98 - 28.66) | 9.23 (22) | 9.22 (2.97) | 3.21 (0.15) | < 0.001 |
| Subarachnoid haemorrhage | 11 | 7380 | 10120 | 61718954 | 9.09 (5.03 - 16.43) | 9.08 (79) | 9.07 (5.02) | 3.18 (1.6) | < 0.001 |
| Cerebral thrombosis | 3 | 7388 | 2794 | 61726280 | 8.97 (2.89 - 27.84) | 8.97 (21.22) | 8.96 (2.89) | 3.16 (0.14) | < 0.001 |
| Confusional state | 166 | 7225 | 160063 | 61569011 | 8.84 (7.58 - 10.31) | 8.66 (1126.77) | 8.65 (7.42) | 3.11 (2.82) | < 0.001 |
| Haemorrhagic stroke | 8 | 7383 | 7778 | 61721296 | 8.6 (4.3 - 17.21) | 8.59 (53.61) | 8.58 (4.29) | 3.1 (1.26) | < 0.001 |
| Status epilepticus | 11 | 7380 | 11196 | 61717878 | 8.22 (4.55 - 14.85) | 8.21 (69.55) | 8.2 (4.54) | 3.04 (1.52) | < 0.001 |
| Influenza like illness | 75 | 7316 | 81664 | 61647410 | 7.74 (6.16 - 9.72) | 7.67 (435.23) | 7.66 (6.1) | 2.94 (2.48) | < 0.001 |
| Cerebral haematoma | 3 | 7388 | 3318 | 61725756 | 7.55 (2.43 - 23.44) | 7.55 (17.04) | 7.55 (2.43) | 2.92 (0.07) | 0.001 |
| Encephalitis | 6 | 7385 | 6854 | 61722220 | 7.32 (3.28 - 16.3) | 7.31 (32.66) | 7.31 (3.28) | 2.87 (0.85) | < 0.001 |
| Depressive symptom | 3 | 7388 | 3477 | 61725597 | 7.21 (2.32 - 22.37) | 7.21 (16.02) | 7.2 (2.32) | 2.85 (0.05) | 0.002 |
| Headache | 478 | 6913 | 618382 | 61110692 | 6.83 (6.23 - 7.5) | 6.46 (2224.56) | 6.45 (5.88) | 2.69 (2.54) | < 0.001 |
| Head discomfort | 14 | 7377 | 17381 | 61711693 | 6.74 (3.99 - 11.39) | 6.73 (68.23) | 6.72 (3.98) | 2.75 (1.54) | < 0.001 |
| Ischaemic stroke | 14 | 7377 | 17669 | 61711405 | 6.63 (3.92 - 11.2) | 6.62 (66.73) | 6.61 (3.91) | 2.73 (1.52) | < 0.001 |
| Conjunctival haemorrhage | 3 | 7388 | 3789 | 61725285 | 6.62 (2.13 - 20.52) | 6.61 (14.28) | 6.61 (2.13) | 2.72 (0.02) | 0.002 |
| Aphasia | 23 | 7368 | 30418 | 61698656 | 6.33 (4.2 - 9.54) | 6.32 (102.86) | 6.31 (4.19) | 2.66 (1.78) | < 0.001 |
| Incontinence | 6 | 7385 | 10492 | 61718582 | 4.78 (2.15 - 10.64) | 4.78 (17.91) | 4.77 (2.14) | 2.26 (0.54) | < 0.001 |
| Tremor | 91 | 7300 | 164275 | 61564799 | 4.67 (3.8 - 5.75) | 4.63 (259.23) | 4.62 (3.76) | 2.21 (1.85) | < 0.001 |
| Fracture | 10 | 7381 | 18653 | 61710421 | 4.48 (2.41 - 8.34) | 4.48 (27) | 4.48 (2.41) | 2.16 (0.89) | < 0.001 |
| Delirium | 18 | 7373 | 34217 | 61694857 | 4.4 (2.77 - 6.99) | 4.39 (47.18) | 4.39 (2.77) | 2.13 (1.23) | < 0.001 |
| Pyrexia | 174 | 7217 | 342287 | 61386787 | 4.32 (3.72 - 5.03) | 4.25 (433.92) | 4.24 (3.65) | 2.09 (1.84) | < 0.001 |
| Lethargy | 29 | 7362 | 56878 | 61672196 | 4.27 (2.97 - 6.15) | 4.26 (72.33) | 4.26 (2.96) | 2.09 (1.41) | < 0.001 |
| Body temperature increased | 10 | 7381 | 20250 | 61708824 | 4.13 (2.22 - 7.68) | 4.12 (23.66) | 4.12 (2.22) | 2.04 (0.81) | < 0.001 |
| Atrial fibrillation | 45 | 7346 | 96686 | 61632388 | 3.9 (2.91 - 5.23) | 3.89 (96.61) | 3.89 (2.9) | 1.96 (1.44) | < 0.001 |
| Hypersomnia | 13 | 7378 | 28275 | 61700799 | 3.84 (2.23 - 6.63) | 3.84 (27.3) | 3.84 (2.23) | 1.94 (0.9) | < 0.001 |
| Mental status changes | 12 | 7379 | 27613 | 61701461 | 3.63 (2.06 - 6.4) | 3.63 (22.86) | 3.63 (2.06) | 1.86 (0.79) | < 0.001 |
| C-reactive protein increased | 15 | 7376 | 36084 | 61692990 | 3.48 (2.09 - 5.77) | 3.47 (26.4) | 3.47 (2.09) | 1.8 (0.87) | < 0.001 |
| Head injury | 13 | 7378 | 31258 | 61697816 | 3.48 (2.02 - 5.99) | 3.47 (22.9) | 3.47 (2.02) | 1.8 (0.79) | < 0.001 |
| Syncope | 41 | 7350 | 99930 | 61629144 | 3.44 (2.53 - 4.68) | 3.43 (70.54) | 3.43 (2.52) | 1.78 (1.25) | < 0.001 |
| Hallucination | 29 | 7362 | 74043 | 61655031 | 3.28 (2.28 - 4.72) | 3.27 (45.77) | 3.27 (2.27) | 1.71 (1.08) | < 0.001 |
| Balance disorder | 33 | 7358 | 85586 | 61643488 | 3.23 (2.29 - 4.55) | 3.22 (50.57) | 3.22 (2.29) | 1.69 (1.1) | < 0.001 |
| Dizziness | 165 | 7226 | 492045 | 61237029 | 2.84 (2.44 - 3.32) | 2.8 (192.5) | 2.8 (2.4) | 1.49 (1.24) | < 0.001 |
| Fall | 104 | 7287 | 328865 | 61400209 | 2.66 (2.2 - 3.23) | 2.64 (106.6) | 2.64 (2.18) | 1.4 (1.09) | < 0.001 |
| Seizure | 54 | 7337 | 171115 | 61557959 | 2.65 (2.03 - 3.46) | 2.64 (54.95) | 2.64 (2.02) | 1.4 (0.96) | < 0.001 |
| Fatigue | 217 | 7174 | 767631 | 60961443 | 2.4 (2.1 - 2.75) | 2.36 (172.34) | 2.36 (2.06) | 1.24 (1.03) | < 0.001 |

Supplementary Table S13. Distribution of adverse events and signal detection results at the Preferred erm (PT) level for Memantine.

| **PT** | **a** | **b** | **c** | **d** | **ROR(95%CI)** | **PRR(X^2^)** | **EBGM(EBGM05)** | **IC(IC025)** | **FDR** |
| --- | --- | --- | --- | --- | --- | --- | --- | --- | --- |
| Myopic chorioretinal degeneration | 4 | 22188 | 24 | 61714249 | 463.57 (160.83 - 1336.17) | 463.49 (1582.25) | 397.42 (137.88) | 8.63 (0.91) | < 0.001 |
| Echolalia | 31 | 22161 | 288 | 61713985 | 299.75 (206.9 - 434.28) | 299.34 (8321.81) | 270.34 (186.6) | 8.08 (4.31) | < 0.001 |
| Mixed incontinence | 6 | 22186 | 86 | 61714187 | 194.07 (84.82 - 444.05) | 194.02 (1077) | 181.43 (79.29) | 7.5 (1.63) | < 0.001 |
| Drug-device interaction | 3 | 22189 | 102 | 61714171 | 81.8 (25.95 - 257.89) | 81.79 (232.57) | 79.48 (25.21) | 6.31 (0.48) | < 0.001 |
| Belligerence | 12 | 22180 | 418 | 61713855 | 79.88 (44.99 - 141.81) | 79.84 (908.11) | 77.64 (43.73) | 6.28 (2.68) | < 0.001 |
| Brachiocephalic vein thrombosis | 3 | 22189 | 119 | 61714154 | 70.12 (22.29 - 220.53) | 70.11 (199.34) | 68.41 (21.75) | 6.1 (0.47) | < 0.001 |
| Illiteracy | 4 | 22188 | 161 | 61714112 | 69.1 (25.62 - 186.38) | 69.09 (261.92) | 67.44 (25) | 6.08 (0.93) | < 0.001 |
| Ophthalmic vein thrombosis | 5 | 22187 | 234 | 61714039 | 59.43 (24.51 - 144.15) | 59.42 (281.18) | 58.2 (24) | 5.86 (1.27) | < 0.001 |
| Repetitive speech | 10 | 22182 | 545 | 61713728 | 51.05 (27.31 - 95.43) | 51.03 (481.62) | 50.12 (26.81) | 5.65 (2.32) | < 0.001 |
| Poriomania | 3 | 22189 | 189 | 61714084 | 44.15 (14.11 - 138.13) | 44.14 (124.52) | 43.47 (13.89) | 5.44 (0.45) | < 0.001 |
| Chorea | 26 | 22166 | 1684 | 61712589 | 42.99 (29.17 - 63.33) | 42.94 (1048.78) | 42.3 (28.71) | 5.4 (3.5) | < 0.001 |
| Perineal infection | 3 | 22189 | 195 | 61714078 | 42.79 (13.68 - 133.84) | 42.78 (120.57) | 42.15 (13.48) | 5.4 (0.44) | < 0.001 |
| Biliary sepsis | 6 | 22186 | 454 | 61713819 | 36.76 (16.43 - 82.27) | 36.75 (205.96) | 36.29 (16.21) | 5.18 (1.49) | < 0.001 |
| Diet refusal | 9 | 22183 | 721 | 61713552 | 34.73 (17.99 - 67.02) | 34.71 (291.05) | 34.3 (17.77) | 5.1 (2.07) | < 0.001 |
| Scleral discolouration | 8 | 22184 | 646 | 61713627 | 34.45 (17.15 - 69.19) | 34.44 (256.57) | 34.03 (16.94) | 5.09 (1.9) | < 0.001 |
| Lack of spontaneous speech | 5 | 22187 | 435 | 61713838 | 31.97 (13.24 - 77.21) | 31.96 (148.28) | 31.61 (13.09) | 4.98 (1.19) | < 0.001 |
| Grunting | 6 | 22186 | 591 | 61713682 | 28.24 (12.63 - 63.12) | 28.23 (156.03) | 27.96 (12.51) | 4.81 (1.43) | < 0.001 |
| Excessive cerumen production | 6 | 22186 | 599 | 61713674 | 27.86 (12.47 - 62.28) | 27.86 (153.81) | 27.59 (12.34) | 4.79 (1.43) | < 0.001 |
| Normal pressure hydrocephalus | 4 | 22188 | 400 | 61713873 | 27.81 (10.39 - 74.48) | 27.81 (102.36) | 27.54 (10.29) | 4.78 (0.83) | < 0.001 |
| Electrocardiogram pr prolongation | 8 | 22184 | 833 | 61713440 | 26.72 (13.31 - 53.61) | 26.71 (196.08) | 26.46 (13.19) | 4.73 (1.82) | < 0.001 |
| Blood pressure orthostatic decreased | 3 | 22189 | 335 | 61713938 | 24.91 (7.99 - 77.62) | 24.9 (68.22) | 24.69 (7.92) | 4.63 (0.38) | < 0.001 |
| Myoclonus | 97 | 22095 | 11710 | 61702563 | 23.13 (18.93 - 28.26) | 23.04 (2028.28) | 22.85 (18.71) | 4.51 (3.93) | < 0.001 |
| Spinal cord oedema | 3 | 22189 | 362 | 61713911 | 23.05 (7.4 - 71.81) | 23.05 (62.75) | 22.87 (7.34) | 4.52 (0.37) | < 0.001 |
| Dropped head syndrome | 3 | 22189 | 381 | 61713892 | 21.9 (7.03 - 68.21) | 21.9 (59.36) | 21.73 (6.98) | 4.44 (0.36) | < 0.001 |
| Abulia | 7 | 22185 | 894 | 61713379 | 21.78 (10.35 - 45.83) | 21.77 (137.67) | 21.61 (10.27) | 4.43 (1.57) | < 0.001 |
| Hypervigilance | 7 | 22185 | 942 | 61713331 | 20.67 (9.83 - 43.49) | 20.67 (130.03) | 20.52 (9.75) | 4.36 (1.55) | < 0.001 |
| Catatonia | 36 | 22156 | 5119 | 61709154 | 19.59 (14.11 - 27.19) | 19.56 (629.53) | 19.43 (13.99) | 4.28 (3.22) | < 0.001 |
| Cystitis noninfective | 9 | 22183 | 1322 | 61712951 | 18.94 (9.83 - 36.49) | 18.93 (151.83) | 18.81 (9.76) | 4.23 (1.84) | < 0.001 |
| Verbal abuse | 3 | 22189 | 484 | 61713789 | 17.24 (5.54 - 53.64) | 17.24 (45.6) | 17.14 (5.51) | 4.1 (0.32) | < 0.001 |
| Pre-existing disease | 3 | 22189 | 485 | 61713788 | 17.2 (5.53 - 53.53) | 17.2 (45.5) | 17.1 (5.5) | 4.1 (0.32) | < 0.001 |
| Libido increased | 12 | 22180 | 1993 | 61712280 | 16.75 (9.5 - 29.55) | 16.74 (176.59) | 16.65 (9.44) | 4.06 (2.11) | < 0.001 |
| Atrioventricular block second degree | 18 | 22174 | 3004 | 61711269 | 16.68 (10.49 - 26.51) | 16.66 (263.45) | 16.57 (10.42) | 4.05 (2.52) | < 0.001 |
| Behaviour disorder | 32 | 22160 | 5411 | 61708862 | 16.47 (11.63 - 23.32) | 16.45 (461.53) | 16.36 (11.55) | 4.03 (2.98) | < 0.001 |
| Confusional state | 878 | 21314 | 159351 | 61554922 | 15.91 (14.87 - 17.03) | 15.32 (11720.44) | 15.24 (14.25) | 3.93 (3.81) | < 0.001 |
| Sleep talking | 9 | 22183 | 1682 | 61712591 | 14.89 (7.73 - 28.66) | 14.88 (115.91) | 14.81 (7.69) | 3.89 (1.72) | < 0.001 |
| Agitation | 383 | 21809 | 72799 | 61641474 | 14.87 (13.44 - 16.46) | 14.63 (4843.99) | 14.56 (13.16) | 3.86 (3.66) | < 0.001 |
| Dermatillomania | 3 | 22189 | 572 | 61713701 | 14.59 (4.69 - 45.37) | 14.59 (37.76) | 14.51 (4.67) | 3.86 (0.28) | < 0.001 |
| Psychotic behaviour | 5 | 22187 | 1009 | 61713264 | 13.78 (5.72 - 33.19) | 13.78 (58.97) | 13.72 (5.7) | 3.78 (0.95) | < 0.001 |
| Bradyarrhythmia | 8 | 22184 | 1623 | 61712650 | 13.71 (6.84 - 27.47) | 13.71 (93.78) | 13.65 (6.81) | 3.77 (1.54) | < 0.001 |
| Postictal state | 5 | 22187 | 1037 | 61713236 | 13.41 (5.57 - 32.29) | 13.41 (57.14) | 13.35 (5.54) | 3.74 (0.94) | < 0.001 |
| Illusion | 8 | 22184 | 1667 | 61712606 | 13.35 (6.66 - 26.74) | 13.35 (90.93) | 13.29 (6.63) | 3.73 (1.53) | < 0.001 |
| Cerebral atrophy | 19 | 22173 | 4012 | 61710261 | 13.18 (8.4 - 20.69) | 13.17 (212.68) | 13.11 (8.35) | 3.71 (2.38) | < 0.001 |
| Aggression | 231 | 21961 | 49408 | 61664865 | 13.13 (11.53 - 14.95) | 13 (2549.33) | 12.95 (11.37) | 3.69 (3.43) | < 0.001 |
| Failure to thrive | 24 | 22168 | 5192 | 61709081 | 12.87 (8.61 - 19.22) | 12.85 (261.2) | 12.8 (8.57) | 3.68 (2.54) | < 0.001 |
| Staring | 11 | 22181 | 2378 | 61711895 | 12.87 (7.12 - 23.27) | 12.86 (119.81) | 12.81 (7.08) | 3.68 (1.86) | < 0.001 |
| Atrioventricular block first degree | 19 | 22173 | 4332 | 61709941 | 12.21 (7.78 - 19.16) | 12.2 (194.46) | 12.15 (7.74) | 3.6 (2.32) | < 0.001 |
| Forearm fracture | 3 | 22189 | 685 | 61713588 | 12.18 (3.92 - 37.86) | 12.18 (30.65) | 12.13 (3.9) | 3.6 (0.23) | < 0.001 |
| Vasogenic cerebral oedema | 3 | 22189 | 697 | 61713576 | 11.97 (3.85 - 37.21) | 11.97 (30.03) | 11.92 (3.84) | 3.58 (0.23) | < 0.001 |
| Drug-disease interaction | 3 | 22189 | 699 | 61713574 | 11.94 (3.84 - 37.1) | 11.94 (29.93) | 11.89 (3.82) | 3.57 (0.23) | < 0.001 |
| Apraxia | 6 | 22186 | 1473 | 61712800 | 11.33 (5.08 - 25.26) | 11.33 (56.27) | 11.29 (5.06) | 3.5 (1.1) | < 0.001 |
| Lung consolidation | 11 | 22181 | 2796 | 61711477 | 10.95 (6.05 - 19.79) | 10.94 (98.97) | 10.9 (6.03) | 3.45 (1.74) | < 0.001 |
| Neuropsychological symptoms | 6 | 22186 | 1527 | 61712746 | 10.93 (4.9 - 24.37) | 10.93 (53.9) | 10.89 (4.88) | 3.44 (1.08) | < 0.001 |
| Violence-related symptom | 6 | 22186 | 1553 | 61712720 | 10.75 (4.82 - 23.96) | 10.74 (52.82) | 10.71 (4.8) | 3.42 (1.07) | < 0.001 |
| Starvation | 3 | 22189 | 790 | 61713483 | 10.56 (3.4 - 32.82) | 10.56 (25.87) | 10.52 (3.39) | 3.4 (0.19) | < 0.001 |
| Abnormal behaviour | 148 | 22044 | 39400 | 61674873 | 10.51 (8.94 - 12.36) | 10.45 (1260.27) | 10.41 (8.85) | 3.38 (3.05) | < 0.001 |
| Atrioventricular block | 28 | 22164 | 7625 | 61706648 | 10.22 (7.05 - 14.82) | 10.21 (231.85) | 10.18 (7.02) | 3.35 (2.41) | < 0.001 |
| Essential tremor | 4 | 22188 | 1105 | 61713168 | 10.07 (3.77 - 26.88) | 10.07 (32.55) | 10.03 (3.76) | 3.33 (0.54) | < 0.001 |
| Metastases to meninges | 8 | 22184 | 2227 | 61712046 | 9.99 (4.99 - 20.01) | 9.99 (64.49) | 9.96 (4.97) | 3.32 (1.35) | < 0.001 |
| Intestinal pseudo-obstruction | 4 | 22188 | 1128 | 61713145 | 9.86 (3.69 - 26.33) | 9.86 (31.74) | 9.83 (3.68) | 3.3 (0.54) | < 0.001 |
| Bradycardia | 182 | 22010 | 52495 | 61661778 | 9.71 (8.39 - 11.24) | 9.64 (1405.95) | 9.61 (8.3) | 3.26 (2.98) | < 0.001 |
| Hypersomnia | 98 | 22094 | 28190 | 61686083 | 9.71 (7.96 - 11.84) | 9.67 (759.27) | 9.64 (7.9) | 3.27 (2.86) | < 0.001 |
| Psychotic symptom | 7 | 22185 | 2006 | 61712267 | 9.71 (4.62 - 20.39) | 9.7 (54.46) | 9.67 (4.61) | 3.27 (1.19) | < 0.001 |
| Mutism | 5 | 22187 | 1432 | 61712841 | 9.71 (4.04 - 23.37) | 9.71 (38.93) | 9.68 (4.02) | 3.27 (0.8) | < 0.001 |
| Non-small cell lung cancer metastatic | 3 | 22189 | 864 | 61713409 | 9.66 (3.11 - 30) | 9.66 (23.2) | 9.63 (3.1) | 3.27 (0.16) | < 0.001 |
| Delusion | 53 | 22139 | 15304 | 61698969 | 9.65 (7.37 - 12.64) | 9.63 (408.62) | 9.6 (7.33) | 3.26 (2.66) | < 0.001 |
| Splenic infarction | 6 | 22186 | 1729 | 61712544 | 9.65 (4.33 - 21.52) | 9.65 (46.36) | 9.62 (4.32) | 3.27 (1.01) | < 0.001 |
| Psychomotor hyperactivity | 57 | 22135 | 16478 | 61697795 | 9.64 (7.43 - 12.51) | 9.62 (438.85) | 9.59 (7.39) | 3.26 (2.68) | < 0.001 |
| Volvulus | 8 | 22184 | 2342 | 61711931 | 9.5 (4.75 - 19.03) | 9.5 (60.63) | 9.47 (4.73) | 3.24 (1.32) | < 0.001 |
| Hallucination, visual | 70 | 22122 | 20699 | 61693574 | 9.43 (7.46 - 11.93) | 9.4 (524.17) | 9.38 (7.41) | 3.23 (2.72) | < 0.001 |
| Inappropriate antidiuretic hormone secretion | 34 | 22158 | 10039 | 61704234 | 9.43 (6.73 - 13.21) | 9.42 (255.01) | 9.39 (6.7) | 3.23 (2.43) | < 0.001 |
| Blood sodium increased | 9 | 22183 | 2680 | 61711593 | 9.34 (4.85 - 17.98) | 9.34 (66.79) | 9.31 (4.84) | 3.22 (1.43) | < 0.001 |
| Hallucination, auditory | 52 | 22140 | 15722 | 61698551 | 9.22 (7.02 - 12.11) | 9.2 (378.78) | 9.17 (6.98) | 3.2 (2.59) | < 0.001 |
| Qrs axis abnormal | 3 | 22189 | 911 | 61713362 | 9.16 (2.95 - 28.45) | 9.16 (21.73) | 9.13 (2.94) | 3.19 (0.14) | 0.001 |
| Urinary incontinence | 94 | 22098 | 29281 | 61684992 | 8.96 (7.32 - 10.98) | 8.93 (659.91) | 8.9 (7.27) | 3.15 (2.74) | < 0.001 |
| Stereotypy | 3 | 22189 | 936 | 61713337 | 8.91 (2.87 - 27.69) | 8.91 (21.01) | 8.89 (2.86) | 3.15 (0.13) | 0.001 |
| Intracranial mass | 4 | 22188 | 1260 | 61713013 | 8.83 (3.31 - 23.57) | 8.83 (27.68) | 8.8 (3.3) | 3.14 (0.49) | < 0.001 |
| Tonic clonic movements | 4 | 22188 | 1281 | 61712992 | 8.68 (3.25 - 23.18) | 8.68 (27.11) | 8.66 (3.24) | 3.11 (0.48) | < 0.001 |
| Hallucination | 226 | 21966 | 73846 | 61640427 | 8.59 (7.53 - 9.79) | 8.51 (1495.22) | 8.49 (7.44) | 3.09 (2.85) | < 0.001 |
| Screaming | 19 | 22173 | 6324 | 61707949 | 8.36 (5.33 - 13.12) | 8.36 (122.66) | 8.33 (5.31) | 3.06 (1.96) | < 0.001 |
| Corneal opacity | 4 | 22188 | 1335 | 61712938 | 8.33 (3.12 - 22.24) | 8.33 (25.73) | 8.31 (3.11) | 3.05 (0.46) | < 0.001 |
| Faecaloma | 16 | 22176 | 5374 | 61708899 | 8.28 (5.07 - 13.54) | 8.28 (102.11) | 8.26 (5.05) | 3.05 (1.83) | < 0.001 |
| Incoherent | 19 | 22173 | 6427 | 61707846 | 8.23 (5.24 - 12.91) | 8.22 (120.17) | 8.2 (5.23) | 3.04 (1.95) | < 0.001 |
| Lymphocyte percentage decreased | 4 | 22188 | 1356 | 61712917 | 8.2 (3.07 - 21.89) | 8.2 (25.23) | 8.18 (3.07) | 3.03 (0.45) | < 0.001 |
| Personality change | 27 | 22165 | 9229 | 61705044 | 8.14 (5.58 - 11.89) | 8.14 (168.52) | 8.11 (5.56) | 3.02 (2.15) | < 0.001 |
| Logorrhoea | 10 | 22182 | 3500 | 61710773 | 7.95 (4.27 - 14.79) | 7.95 (60.54) | 7.93 (4.26) | 2.99 (1.41) | < 0.001 |
| Hypernatraemia | 14 | 22178 | 4909 | 61709364 | 7.94 (4.7 - 13.41) | 7.93 (84.56) | 7.91 (4.68) | 2.98 (1.69) | < 0.001 |
| Bundle branch block right | 13 | 22179 | 4611 | 61709662 | 7.84 (4.55 - 13.52) | 7.84 (77.37) | 7.82 (4.54) | 2.97 (1.62) | < 0.001 |
| Petit mal epilepsy | 14 | 22178 | 5027 | 61709246 | 7.75 (4.59 - 13.1) | 7.74 (82.01) | 7.73 (4.57) | 2.95 (1.67) | < 0.001 |
| Paranoia | 47 | 22145 | 17101 | 61697172 | 7.66 (5.75 - 10.2) | 7.64 (270.7) | 7.62 (5.72) | 2.93 (2.33) | < 0.001 |
| Conduction disorder | 5 | 22187 | 1816 | 61712457 | 7.66 (3.18 - 18.42) | 7.66 (28.86) | 7.64 (3.18) | 2.93 (0.68) | < 0.001 |
| Ataxia | 32 | 22160 | 11779 | 61702494 | 7.56 (5.35 - 10.7) | 7.55 (181.54) | 7.54 (5.33) | 2.91 (2.15) | < 0.001 |
| Psychomotor skills impaired | 7 | 22185 | 2594 | 61711679 | 7.51 (3.57 - 15.76) | 7.5 (39.36) | 7.49 (3.57) | 2.9 (1.03) | < 0.001 |
| Mental status changes | 74 | 22118 | 27551 | 61686722 | 7.49 (5.96 - 9.41) | 7.47 (413.71) | 7.45 (5.93) | 2.9 (2.44) | < 0.001 |
| Incontinence | 28 | 22164 | 10470 | 61703803 | 7.45 (5.14 - 10.79) | 7.44 (155.61) | 7.42 (5.12) | 2.89 (2.07) | < 0.001 |
| Hallucinations, mixed | 11 | 22181 | 4151 | 61710122 | 7.37 (4.08 - 13.33) | 7.37 (60.4) | 7.35 (4.07) | 2.88 (1.43) | < 0.001 |
| Yawning | 7 | 22185 | 2643 | 61711630 | 7.37 (3.51 - 15.47) | 7.37 (38.41) | 7.35 (3.5) | 2.88 (1.01) | < 0.001 |
| Delirium | 90 | 22102 | 34145 | 61680128 | 7.36 (5.98 - 9.05) | 7.33 (490.96) | 7.31 (5.94) | 2.87 (2.47) | < 0.001 |
| Subclavian vein thrombosis | 3 | 22189 | 1139 | 61713134 | 7.33 (2.36 - 22.75) | 7.32 (16.34) | 7.31 (2.35) | 2.87 (0.06) | 0.002 |
| Hypertensive crisis | 29 | 22163 | 11029 | 61703244 | 7.32 (5.08 - 10.54) | 7.31 (157.64) | 7.3 (5.07) | 2.87 (2.06) | < 0.001 |
| Sinus arrest | 4 | 22188 | 1527 | 61712746 | 7.29 (2.73 - 19.44) | 7.28 (21.63) | 7.27 (2.72) | 2.86 (0.4) | < 0.001 |
| Cerebral ventricle dilatation | 3 | 22189 | 1156 | 61713117 | 7.22 (2.32 - 22.41) | 7.22 (16.03) | 7.2 (2.32) | 2.85 (0.05) | 0.002 |
| Labile blood pressure | 3 | 22189 | 1180 | 61713093 | 7.07 (2.28 - 21.96) | 7.07 (15.6) | 7.05 (2.27) | 2.82 (0.04) | 0.002 |
| Wrong dose | 6 | 22186 | 2388 | 61711885 | 6.99 (3.14 - 15.57) | 6.99 (30.71) | 6.97 (3.13) | 2.8 (0.82) | < 0.001 |
| Lacunar infarction | 6 | 22186 | 2423 | 61711850 | 6.89 (3.09 - 15.35) | 6.89 (30.12) | 6.87 (3.08) | 2.78 (0.81) | < 0.001 |
| Listless | 8 | 22184 | 3246 | 61711027 | 6.86 (3.43 - 13.72) | 6.85 (39.9) | 6.84 (3.42) | 2.77 (1.09) | < 0.001 |
| Fluid intake reduced | 12 | 22180 | 4874 | 61709399 | 6.85 (3.89 - 12.07) | 6.85 (59.77) | 6.83 (3.88) | 2.77 (1.44) | < 0.001 |
| Somnolence | 478 | 21714 | 198345 | 61515928 | 6.83 (6.24 - 7.48) | 6.7 (2320.72) | 6.69 (6.11) | 2.74 (2.59) | < 0.001 |
| Social avoidant behaviour | 14 | 22178 | 5738 | 61708535 | 6.79 (4.02 - 11.47) | 6.79 (68.89) | 6.77 (4.01) | 2.76 (1.54) | < 0.001 |
| Sedation complication | 6 | 22186 | 2465 | 61711808 | 6.77 (3.04 - 15.09) | 6.77 (29.43) | 6.75 (3.03) | 2.76 (0.8) | < 0.001 |
| Dermatomyositis | 6 | 22186 | 2506 | 61711767 | 6.66 (2.99 - 14.84) | 6.66 (28.78) | 6.64 (2.98) | 2.73 (0.79) | < 0.001 |
| Supraventricular extrasystoles | 8 | 22184 | 3404 | 61710869 | 6.54 (3.27 - 13.09) | 6.54 (37.42) | 6.52 (3.26) | 2.71 (1.05) | < 0.001 |
| Metastatic neoplasm | 10 | 22182 | 4292 | 61709981 | 6.48 (3.48 - 12.06) | 6.48 (46.23) | 6.47 (3.48) | 2.69 (1.24) | < 0.001 |
| Corneal disorder | 5 | 22187 | 2152 | 61712121 | 6.46 (2.69 - 15.54) | 6.46 (23.03) | 6.45 (2.68) | 2.69 (0.58) | < 0.001 |
| Disorganised speech | 4 | 22188 | 1784 | 61712489 | 6.24 (2.34 - 16.64) | 6.24 (17.54) | 6.22 (2.33) | 2.64 (0.31) | < 0.001 |
| Altered state of consciousness | 47 | 22145 | 21300 | 61692973 | 6.15 (4.62 - 8.19) | 6.14 (201.69) | 6.13 (4.6) | 2.61 (2.05) | < 0.001 |
| White blood cells urine positive | 5 | 22187 | 2313 | 61711960 | 6.01 (2.5 - 14.46) | 6.01 (20.85) | 6 (2.5) | 2.59 (0.53) | < 0.001 |
| Electrocardiogram qt prolonged | 77 | 22115 | 36152 | 61678121 | 5.94 (4.75 - 7.43) | 5.92 (314.59) | 5.91 (4.73) | 2.56 (2.15) | < 0.001 |
| Lethargy | 120 | 22072 | 56787 | 61657486 | 5.9 (4.93 - 7.06) | 5.88 (485.03) | 5.87 (4.9) | 2.55 (2.23) | < 0.001 |
| Pancreatic carcinoma metastatic | 6 | 22186 | 2843 | 61711430 | 5.87 (2.63 - 13.08) | 5.87 (24.19) | 5.86 (2.63) | 2.55 (0.7) | < 0.001 |
| Dyskinesia | 84 | 22108 | 40014 | 61674259 | 5.86 (4.73 - 7.26) | 5.84 (336.29) | 5.83 (4.7) | 2.54 (2.15) | < 0.001 |
| Sedation | 54 | 22138 | 25754 | 61688519 | 5.84 (4.47 - 7.63) | 5.83 (215.77) | 5.82 (4.46) | 2.54 (2.03) | < 0.001 |
| Parkinsonism | 20 | 22172 | 9614 | 61704659 | 5.79 (3.73 - 8.98) | 5.79 (79.01) | 5.78 (3.72) | 2.53 (1.6) | < 0.001 |
| Drooling | 14 | 22178 | 6791 | 61707482 | 5.74 (3.39 - 9.69) | 5.73 (54.6) | 5.72 (3.39) | 2.52 (1.38) | < 0.001 |
| Aphasia | 61 | 22131 | 30380 | 61683893 | 5.6 (4.35 - 7.2) | 5.58 (229.19) | 5.57 (4.33) | 2.48 (2.01) | < 0.001 |
| Metabolic encephalopathy | 6 | 22186 | 2978 | 61711295 | 5.6 (2.52 - 12.49) | 5.6 (22.64) | 5.59 (2.51) | 2.48 (0.66) | < 0.001 |
| Enuresis | 8 | 22184 | 3992 | 61710281 | 5.57 (2.79 - 11.16) | 5.57 (29.96) | 5.56 (2.78) | 2.48 (0.92) | < 0.001 |
| Depressed level of consciousness | 77 | 22115 | 39186 | 61675087 | 5.48 (4.38 - 6.86) | 5.46 (280.48) | 5.46 (4.36) | 2.45 (2.04) | < 0.001 |
| Hostility | 5 | 22187 | 2537 | 61711736 | 5.48 (2.28 - 13.18) | 5.48 (18.28) | 5.47 (2.28) | 2.45 (0.47) | < 0.001 |
| Drug titration error | 5 | 22187 | 2549 | 61711724 | 5.46 (2.27 - 13.12) | 5.45 (18.16) | 5.45 (2.26) | 2.45 (0.47) | < 0.001 |
| Hypomania | 7 | 22185 | 3649 | 61710624 | 5.34 (2.54 - 11.2) | 5.33 (24.61) | 5.33 (2.54) | 2.41 (0.77) | < 0.001 |
| Orthostatic hypotension | 33 | 22159 | 17278 | 61696995 | 5.32 (3.78 - 7.48) | 5.31 (115.3) | 5.3 (3.77) | 2.41 (1.74) | < 0.001 |
| Hyperacusis | 7 | 22185 | 3689 | 61710584 | 5.28 (2.51 - 11.08) | 5.28 (24.22) | 5.27 (2.51) | 2.4 (0.76) | < 0.001 |
| Electroencephalogram abnormal | 6 | 22186 | 3161 | 61711112 | 5.28 (2.37 - 11.76) | 5.28 (20.77) | 5.27 (2.37) | 2.4 (0.62) | < 0.001 |
| Metastases to central nervous system | 24 | 22168 | 12691 | 61701582 | 5.26 (3.53 - 7.86) | 5.26 (82.64) | 5.25 (3.52) | 2.39 (1.59) | < 0.001 |
| Stupor | 6 | 22186 | 3205 | 61711068 | 5.21 (2.34 - 11.6) | 5.21 (20.35) | 5.2 (2.33) | 2.38 (0.61) | < 0.001 |
| Immobile | 8 | 22184 | 4290 | 61709983 | 5.19 (2.59 - 10.38) | 5.19 (26.98) | 5.18 (2.59) | 2.37 (0.86) | < 0.001 |
| Posture abnormal | 6 | 22186 | 3215 | 61711058 | 5.19 (2.33 - 11.56) | 5.19 (20.26) | 5.18 (2.33) | 2.37 (0.61) | < 0.001 |
| Hypophagia | 52 | 22140 | 28044 | 61686229 | 5.17 (3.93 - 6.78) | 5.16 (173.98) | 5.15 (3.92) | 2.36 (1.86) | < 0.001 |
| Toxic encephalopathy | 9 | 22183 | 4997 | 61709276 | 5.01 (2.61 - 9.64) | 5.01 (28.83) | 5 (2.6) | 2.32 (0.92) | < 0.001 |
| Coordination abnormal | 25 | 22167 | 13909 | 61700364 | 5 (3.38 - 7.41) | 5 (79.84) | 4.99 (3.37) | 2.32 (1.55) | < 0.001 |
| Mania | 29 | 22163 | 16234 | 61698039 | 4.97 (3.45 - 7.16) | 4.97 (91.76) | 4.96 (3.45) | 2.31 (1.6) | < 0.001 |
| Epilepsy | 53 | 22139 | 30103 | 61684170 | 4.91 (3.75 - 6.42) | 4.9 (164.11) | 4.89 (3.73) | 2.29 (1.8) | < 0.001 |
| Torsade de pointes | 13 | 22179 | 7769 | 61706504 | 4.66 (2.7 - 8.02) | 4.65 (37.23) | 4.65 (2.7) | 2.22 (1.11) | < 0.001 |
| Mental impairment | 40 | 22152 | 23972 | 61690301 | 4.65 (3.41 - 6.34) | 4.64 (114.09) | 4.63 (3.4) | 2.21 (1.64) | < 0.001 |
| Paradoxical drug reaction | 6 | 22186 | 3623 | 61710650 | 4.61 (2.07 - 10.26) | 4.61 (16.91) | 4.6 (2.06) | 2.2 (0.51) | < 0.001 |
| Bundle branch block left | 7 | 22185 | 4251 | 61710022 | 4.58 (2.18 - 9.61) | 4.58 (19.55) | 4.57 (2.18) | 2.19 (0.64) | < 0.001 |
| Anger | 55 | 22137 | 33542 | 61680731 | 4.57 (3.51 - 5.95) | 4.56 (152.69) | 4.55 (3.49) | 2.19 (1.71) | < 0.001 |
| Pemphigoid | 12 | 22180 | 7305 | 61706968 | 4.57 (2.59 - 8.05) | 4.57 (33.39) | 4.56 (2.59) | 2.19 (1.04) | < 0.001 |
| Ventricular extrasystoles | 17 | 22175 | 10371 | 61703902 | 4.56 (2.83 - 7.34) | 4.56 (47.15) | 4.55 (2.83) | 2.19 (1.25) | < 0.001 |
| Anal incontinence | 22 | 22170 | 13618 | 61700655 | 4.5 (2.96 - 6.83) | 4.49 (59.65) | 4.49 (2.95) | 2.17 (1.36) | < 0.001 |
| Decreased activity | 17 | 22175 | 10564 | 61703709 | 4.48 (2.78 - 7.21) | 4.48 (45.81) | 4.47 (2.78) | 2.16 (1.22) | < 0.001 |
| Crying | 57 | 22135 | 36042 | 61678231 | 4.41 (3.4 - 5.72) | 4.4 (149.5) | 4.39 (3.39) | 2.14 (1.67) | < 0.001 |
| Generalised tonic-clonic seizure | 40 | 22152 | 25496 | 61688777 | 4.37 (3.2 - 5.96) | 4.36 (103.57) | 4.36 (3.19) | 2.12 (1.56) | < 0.001 |
| Hyponatraemia | 86 | 22106 | 55583 | 61658690 | 4.32 (3.49 - 5.33) | 4.3 (217.88) | 4.3 (3.48) | 2.1 (1.74) | < 0.001 |
| Dizziness | 731 | 21461 | 491479 | 61222794 | 4.24 (3.94 - 4.57) | 4.14 (1749.66) | 4.13 (3.84) | 2.05 (1.93) | < 0.001 |
| Speech disorder | 77 | 22115 | 51783 | 61662490 | 4.15 (3.31 - 5.19) | 4.14 (182.91) | 4.13 (3.3) | 2.05 (1.66) | < 0.001 |
| Restlessness | 54 | 22138 | 36245 | 61678028 | 4.15 (3.18 - 5.42) | 4.14 (128.65) | 4.14 (3.17) | 2.05 (1.58) | < 0.001 |
| Syncope | 139 | 22053 | 99832 | 61614441 | 3.89 (3.29 - 4.6) | 3.87 (296.17) | 3.87 (3.27) | 1.95 (1.68) | < 0.001 |
| Urinary retention | 46 | 22146 | 33011 | 61681262 | 3.88 (2.91 - 5.18) | 3.88 (98.04) | 3.87 (2.9) | 1.95 (1.45) | < 0.001 |
| Apathy | 20 | 22172 | 14352 | 61699921 | 3.88 (2.5 - 6.01) | 3.88 (42.62) | 3.87 (2.5) | 1.95 (1.14) | < 0.001 |
| Fall | 444 | 21748 | 328525 | 61385748 | 3.81 (3.47 - 4.19) | 3.76 (902.46) | 3.75 (3.42) | 1.91 (1.76) | < 0.001 |
| International normalised ratio increased | 39 | 22153 | 28527 | 61685746 | 3.81 (2.78 - 5.21) | 3.8 (80.46) | 3.8 (2.77) | 1.93 (1.37) | < 0.001 |
| Neuroleptic malignant syndrome | 16 | 22176 | 11743 | 61702530 | 3.79 (2.32 - 6.19) | 3.79 (32.81) | 3.79 (2.32) | 1.92 (1) | < 0.001 |
| Encephalopathy | 32 | 22160 | 24239 | 61690034 | 3.68 (2.6 - 5.2) | 3.67 (62.14) | 3.67 (2.59) | 1.87 (1.26) | < 0.001 |
| Balance disorder | 110 | 22082 | 85509 | 61628764 | 3.59 (2.98 - 4.33) | 3.58 (204.29) | 3.57 (2.96) | 1.84 (1.53) | < 0.001 |
| Blood pressure systolic increased | 25 | 22167 | 19400 | 61694873 | 3.59 (2.42 - 5.31) | 3.58 (46.52) | 3.58 (2.42) | 1.84 (1.14) | < 0.001 |
| Eating disorder | 27 | 22165 | 21102 | 61693171 | 3.56 (2.44 - 5.2) | 3.56 (49.61) | 3.55 (2.44) | 1.83 (1.16) | < 0.001 |
| Abnormal dreams | 35 | 22157 | 27443 | 61686830 | 3.55 (2.55 - 4.95) | 3.55 (63.95) | 3.54 (2.54) | 1.83 (1.24) | < 0.001 |
| Muscle twitching | 29 | 22163 | 22833 | 61691440 | 3.54 (2.46 - 5.09) | 3.53 (52.59) | 3.53 (2.45) | 1.82 (1.17) | < 0.001 |
| Seizure | 208 | 21984 | 170961 | 61543312 | 3.41 (2.97 - 3.9) | 3.38 (349.77) | 3.38 (2.95) | 1.76 (1.54) | < 0.001 |
| Jaundice | 32 | 22160 | 26704 | 61687569 | 3.34 (2.36 - 4.72) | 3.33 (52.2) | 3.33 (2.35) | 1.74 (1.13) | < 0.001 |
| Psychotic disorder | 35 | 22157 | 29228 | 61685045 | 3.33 (2.39 - 4.65) | 3.33 (57.02) | 3.33 (2.39) | 1.73 (1.16) | < 0.001 |
| Pulmonary embolism | 107 | 22085 | 94363 | 61619910 | 3.16 (2.62 - 3.83) | 3.15 (157.4) | 3.15 (2.61) | 1.66 (1.35) | < 0.001 |
| Constipation | 236 | 21956 | 213746 | 61500527 | 3.09 (2.72 - 3.52) | 3.07 (330.27) | 3.07 (2.7) | 1.62 (1.42) | < 0.001 |
| Gait disturbance | 209 | 21983 | 192875 | 61521398 | 3.03 (2.65 - 3.48) | 3.01 (281.74) | 3.01 (2.63) | 1.59 (1.38) | < 0.001 |
| Loss of consciousness | 135 | 22057 | 126775 | 61587498 | 2.97 (2.51 - 3.52) | 2.96 (175.54) | 2.96 (2.5) | 1.57 (1.3) | < 0.001 |
| Liver function test abnormal | 30 | 22162 | 28956 | 61685317 | 2.88 (2.02 - 4.13) | 2.88 (36.83) | 2.88 (2.01) | 1.53 (0.92) | < 0.001 |
| Pollakiuria | 41 | 22151 | 41149 | 61673124 | 2.77 (2.04 - 3.77) | 2.77 (46.39) | 2.77 (2.04) | 1.47 (0.96) | < 0.001 |
| Tremor | 152 | 22040 | 164214 | 61550059 | 2.58 (2.2 - 3.03) | 2.57 (146.57) | 2.57 (2.19) | 1.36 (1.11) | < 0.001 |
| Asthenia | 331 | 21861 | 371819 | 61342454 | 2.5 (2.24 - 2.78) | 2.48 (292.64) | 2.47 (2.22) | 1.31 (1.14) | < 0.001 |
| Condition aggravated | 258 | 21934 | 298434 | 61415839 | 2.42 (2.14 - 2.74) | 2.4 (212.43) | 2.4 (2.13) | 1.26 (1.08) | < 0.001 |
| Decreased appetite | 203 | 21989 | 241716 | 61472557 | 2.35 (2.04 - 2.7) | 2.34 (155.51) | 2.33 (2.03) | 1.22 (1.01) | < 0.001 |

Supplementary Table S14. Distribution of adverse events and signal detection results at the Preferred erm (PT) level for Rivastigmine.

| **PT** | **a** | **b** | **c** | **d** | **ROR(95%CI)** | **PRR(X^2^)** | **EBGM(EBGM05)** | **IC(IC025)** | **FDR** |
| --- | --- | --- | --- | --- | --- | --- | --- | --- | --- |
| Accessory cardiac pathway | 6 | 46536 | 15 | 61689908 | 530.26 (205.73 - 1366.72) | 530.19 (2263.67) | 378.99 (147.04) | 8.57 (1.53) | < 0.001 |
| Mini mental status examination abnormal | 19 | 46523 | 62 | 61689861 | 406.36 (243.03 - 679.44) | 406.19 (5878.29) | 311.15 (186.09) | 8.28 (3.52) | < 0.001 |
| Lack of application site rotation | 29 | 46513 | 120 | 61689803 | 320.52 (213.64 - 480.88) | 320.32 (7434.71) | 258.17 (172.08) | 8.01 (4.18) | < 0.001 |
| Blood cholinesterase decreased | 46 | 46496 | 232 | 61689691 | 263.07 (191.7 - 361) | 262.81 (10012.23) | 219.49 (159.94) | 7.78 (4.82) | < 0.001 |
| Cholinergic syndrome | 55 | 46487 | 577 | 61689346 | 126.49 (95.91 - 166.82) | 126.34 (6244.24) | 115.44 (87.53) | 6.85 (4.84) | < 0.001 |
| Application site hypersensitivity | 100 | 46442 | 1091 | 61688832 | 121.75 (99.18 - 149.45) | 121.49 (10946.79) | 111.37 (90.73) | 6.8 (5.43) | < 0.001 |
| Bronchial aspiration procedure | 22 | 46520 | 299 | 61689624 | 97.57 (63.28 - 150.45) | 97.53 (1957.76) | 90.91 (58.96) | 6.51 (3.59) | < 0.001 |
| Corticobasal degeneration | 3 | 46539 | 54 | 61689869 | 73.64 (23.02 - 235.54) | 73.64 (203.64) | 69.81 (21.83) | 6.13 (0.45) | < 0.001 |
| Application site eczema | 4 | 46538 | 80 | 61689843 | 66.28 (24.28 - 180.93) | 66.27 (244.91) | 63.17 (23.14) | 5.98 (0.9) | < 0.001 |
| Poriomania | 9 | 46533 | 183 | 61689740 | 65.2 (33.39 - 127.32) | 65.19 (542.16) | 62.18 (31.84) | 5.96 (2.19) | < 0.001 |
| Medical device site dryness | 3 | 46539 | 61 | 61689862 | 65.19 (20.45 - 207.78) | 65.19 (180.72) | 62.18 (19.51) | 5.96 (0.44) | < 0.001 |
| Agnosia | 10 | 46532 | 224 | 61689699 | 59.19 (31.41 - 111.52) | 59.17 (547.46) | 56.69 (30.08) | 5.82 (2.33) | < 0.001 |
| Frontotemporal dementia | 14 | 46528 | 409 | 61689514 | 45.38 (26.64 - 77.32) | 45.37 (587.39) | 43.9 (25.77) | 5.46 (2.75) | < 0.001 |
| Application site mass | 9 | 46533 | 267 | 61689656 | 44.69 (23 - 86.83) | 44.68 (371.78) | 43.25 (22.26) | 5.43 (2.12) | < 0.001 |
| Application site injury | 6 | 46536 | 179 | 61689744 | 44.43 (19.7 - 100.24) | 44.43 (246.45) | 43.02 (19.07) | 5.43 (1.51) | < 0.001 |
| Application site dermatitis | 27 | 46515 | 901 | 61689022 | 39.74 (27.1 - 58.28) | 39.72 (989.48) | 38.59 (26.32) | 5.27 (3.49) | < 0.001 |
| Stubbornness | 11 | 46531 | 418 | 61689505 | 34.89 (19.17 - 63.49) | 34.88 (352.72) | 34.01 (18.69) | 5.09 (2.34) | < 0.001 |
| Prosopagnosia | 4 | 46538 | 155 | 61689768 | 34.21 (12.68 - 92.3) | 34.21 (125.7) | 33.37 (12.37) | 5.06 (0.85) | < 0.001 |
| Lack of spontaneous speech | 11 | 46531 | 429 | 61689494 | 33.99 (18.68 - 61.85) | 33.99 (343.37) | 33.16 (18.23) | 5.05 (2.33) | < 0.001 |
| Vascular dementia | 21 | 46521 | 847 | 61689076 | 32.88 (21.32 - 50.7) | 32.86 (633.07) | 32.09 (20.81) | 5 (3.11) | < 0.001 |
| Oesophageal dilatation | 10 | 46532 | 424 | 61689499 | 31.27 (16.7 - 58.54) | 31.26 (286.18) | 30.56 (16.32) | 4.93 (2.17) | < 0.001 |
| Diastolic hypertension | 3 | 46539 | 128 | 61689795 | 31.07 (9.89 - 97.61) | 31.07 (85.29) | 30.38 (9.67) | 4.92 (0.4) | < 0.001 |
| Marasmus | 34 | 46508 | 1462 | 61688461 | 30.85 (21.95 - 43.34) | 30.82 (958.87) | 30.15 (21.45) | 4.91 (3.55) | < 0.001 |
| Pleurothotonus | 20 | 46522 | 882 | 61689041 | 30.07 (19.3 - 46.84) | 30.06 (549.34) | 29.41 (18.88) | 4.88 (3.01) | < 0.001 |
| Regressive behaviour | 6 | 46536 | 285 | 61689638 | 27.91 (12.43 - 62.65) | 27.9 (152.43) | 27.35 (12.18) | 4.77 (1.42) | < 0.001 |
| Vascular fragility | 3 | 46539 | 150 | 61689773 | 26.51 (8.45 - 83.13) | 26.51 (72.2) | 26.01 (8.29) | 4.7 (0.38) | < 0.001 |
| Application site wound | 3 | 46539 | 153 | 61689770 | 25.99 (8.29 - 81.49) | 25.99 (70.7) | 25.51 (8.14) | 4.67 (0.38) | < 0.001 |
| Neurodegenerative disorder | 11 | 46531 | 590 | 61689333 | 24.72 (13.61 - 44.88) | 24.71 (245.7) | 24.28 (13.37) | 4.6 (2.2) | < 0.001 |
| Gastric lavage | 3 | 46539 | 161 | 61689762 | 24.7 (7.88 - 77.4) | 24.7 (66.97) | 24.26 (7.74) | 4.6 (0.37) | < 0.001 |
| Repetitive speech | 10 | 46532 | 545 | 61689378 | 24.33 (13.01 - 45.47) | 24.32 (219.59) | 23.9 (12.79) | 4.58 (2.08) | < 0.001 |
| Application site erythema | 433 | 46109 | 25564 | 61664359 | 22.65 (20.59 - 24.92) | 22.45 (8730.27) | 22.09 (20.08) | 4.47 (4.26) | < 0.001 |
| Skin pressure mark | 3 | 46539 | 181 | 61689742 | 21.97 (7.02 - 68.76) | 21.97 (59.06) | 21.63 (6.91) | 4.43 (0.35) | < 0.001 |
| Parkinsonian rest tremor | 3 | 46539 | 185 | 61689738 | 21.5 (6.87 - 67.26) | 21.49 (57.69) | 21.17 (6.76) | 4.4 (0.35) | < 0.001 |
| Dysentery | 28 | 46514 | 1801 | 61688122 | 20.62 (14.19 - 29.95) | 20.61 (514.37) | 20.31 (13.98) | 4.34 (3.07) | < 0.001 |
| Eschar | 11 | 46531 | 728 | 61689195 | 20.03 (11.04 - 36.34) | 20.03 (195.9) | 19.74 (10.89) | 4.3 (2.11) | < 0.001 |
| Joint warmth | 18 | 46524 | 1204 | 61688719 | 19.82 (12.45 - 31.57) | 19.82 (316.86) | 19.54 (12.27) | 4.29 (2.64) | < 0.001 |
| Parkinsonian gait | 9 | 46533 | 606 | 61689317 | 19.69 (10.19 - 38.03) | 19.69 (157.29) | 19.41 (10.05) | 4.28 (1.85) | < 0.001 |
| Vascular encephalopathy | 4 | 46538 | 281 | 61689642 | 18.87 (7.03 - 50.63) | 18.87 (66.73) | 18.62 (6.94) | 4.22 (0.74) | < 0.001 |
| Application site pruritus | 307 | 46235 | 22115 | 61667808 | 18.52 (16.54 - 20.73) | 18.4 (4984.17) | 18.16 (16.22) | 4.18 (3.94) | < 0.001 |
| Concomitant disease progression | 27 | 46515 | 1936 | 61687987 | 18.5 (12.65 - 27.04) | 18.49 (440.44) | 18.24 (12.48) | 4.19 (2.95) | < 0.001 |
| Diet refusal | 10 | 46532 | 720 | 61689203 | 18.41 (9.86 - 34.37) | 18.41 (162.38) | 18.17 (9.73) | 4.18 (1.95) | < 0.001 |
| Scintillating scotoma | 3 | 46539 | 217 | 61689706 | 18.33 (5.86 - 57.27) | 18.32 (48.47) | 18.09 (5.79) | 4.18 (0.32) | < 0.001 |
| Parkinson's disease psychosis | 3 | 46539 | 226 | 61689697 | 17.6 (5.63 - 54.97) | 17.59 (46.34) | 17.38 (5.56) | 4.12 (0.31) | < 0.001 |
| Resting tremor | 8 | 46534 | 633 | 61689290 | 16.75 (8.34 - 33.65) | 16.75 (117.01) | 16.55 (8.24) | 4.05 (1.63) | < 0.001 |
| Parkinson's disease | 227 | 46315 | 18772 | 61671151 | 16.1 (14.12 - 18.36) | 16.03 (3161.32) | 15.85 (13.9) | 3.99 (3.7) | < 0.001 |
| Heat illness | 8 | 46534 | 663 | 61689260 | 16 (7.97 - 32.12) | 15.99 (111.11) | 15.81 (7.88) | 3.98 (1.61) | < 0.001 |
| Reduced facial expression | 16 | 46526 | 1382 | 61688541 | 15.35 (9.38 - 25.13) | 15.35 (212.12) | 15.18 (9.27) | 3.92 (2.34) | < 0.001 |
| Infarction | 77 | 46465 | 6755 | 61683168 | 15.13 (12.09 - 18.95) | 15.11 (1003.16) | 14.95 (11.94) | 3.9 (3.34) | < 0.001 |
| Oral discharge | 4 | 46538 | 357 | 61689566 | 14.85 (5.54 - 39.79) | 14.85 (51.1) | 14.7 (5.49) | 3.88 (0.67) | < 0.001 |
| Application site inflammation | 15 | 46527 | 1341 | 61688582 | 14.83 (8.91 - 24.67) | 14.83 (191.27) | 14.67 (8.82) | 3.88 (2.26) | < 0.001 |
| Sputum increased | 23 | 46519 | 2065 | 61687858 | 14.77 (9.79 - 22.28) | 14.76 (291.87) | 14.61 (9.69) | 3.87 (2.63) | < 0.001 |
| Nosocomial infection | 24 | 46518 | 2177 | 61687746 | 14.62 (9.78 - 21.86) | 14.61 (301.03) | 14.46 (9.67) | 3.85 (2.65) | < 0.001 |
| Application site rash | 143 | 46399 | 13485 | 61676438 | 14.1 (11.95 - 16.63) | 14.06 (1716.33) | 13.92 (11.8) | 3.8 (3.43) | < 0.001 |
| Application site discolouration | 42 | 46500 | 4010 | 61685913 | 13.89 (10.25 - 18.83) | 13.88 (496.93) | 13.75 (10.14) | 3.78 (2.96) | < 0.001 |
| Concomitant disease aggravated | 61 | 46481 | 6048 | 61683875 | 13.38 (10.4 - 17.23) | 13.37 (691.15) | 13.25 (10.29) | 3.73 (3.1) | < 0.001 |
| Apparent death | 36 | 46506 | 3568 | 61686355 | 13.38 (9.64 - 18.59) | 13.37 (408.05) | 13.25 (9.54) | 3.73 (2.84) | < 0.001 |
| Normal pressure hydrocephalus | 4 | 46538 | 400 | 61689523 | 13.26 (4.95 - 35.49) | 13.25 (44.87) | 13.13 (4.9) | 3.72 (0.64) | < 0.001 |
| Prosthesis implantation | 3 | 46539 | 302 | 61689621 | 13.17 (4.22 - 41.06) | 13.17 (33.4) | 13.05 (4.18) | 3.71 (0.25) | < 0.001 |
| Akinesia | 20 | 46522 | 2039 | 61687884 | 13.01 (8.37 - 20.21) | 13 (219.42) | 12.88 (8.29) | 3.69 (2.41) | < 0.001 |
| Application site reaction | 67 | 46475 | 6940 | 61682983 | 12.81 (10.07 - 16.3) | 12.8 (721.7) | 12.68 (9.97) | 3.66 (3.08) | < 0.001 |
| Application site oedema | 3 | 46539 | 317 | 61689606 | 12.54 (4.02 - 39.11) | 12.54 (31.57) | 12.44 (3.99) | 3.64 (0.24) | < 0.001 |
| Stoma site irritation | 5 | 46537 | 535 | 61689388 | 12.39 (5.14 - 29.89) | 12.39 (51.86) | 12.28 (5.09) | 3.62 (0.91) | < 0.001 |
| Chikungunya virus infection | 5 | 46537 | 554 | 61689369 | 11.96 (4.96 - 28.86) | 11.96 (49.78) | 11.86 (4.92) | 3.57 (0.89) | < 0.001 |
| Soft tissue mass | 4 | 46538 | 453 | 61689470 | 11.7 (4.37 - 31.32) | 11.7 (38.81) | 11.61 (4.34) | 3.54 (0.6) | < 0.001 |
| Fibroma | 8 | 46534 | 911 | 61689012 | 11.64 (5.8 - 23.35) | 11.64 (77.13) | 11.55 (5.76) | 3.53 (1.44) | < 0.001 |
| Suspiciousness | 6 | 46536 | 685 | 61689238 | 11.61 (5.2 - 25.94) | 11.61 (57.67) | 11.52 (5.16) | 3.53 (1.11) | < 0.001 |
| Multiple system atrophy | 3 | 46539 | 346 | 61689577 | 11.49 (3.69 - 35.81) | 11.49 (28.49) | 11.4 (3.66) | 3.51 (0.21) | < 0.001 |
| Unmasking of previously unidentified disease | 9 | 46533 | 1051 | 61688872 | 11.35 (5.89 - 21.88) | 11.35 (84.23) | 11.26 (5.84) | 3.49 (1.56) | < 0.001 |
| Helplessness | 4 | 46538 | 468 | 61689455 | 11.33 (4.23 - 30.32) | 11.33 (37.35) | 11.24 (4.2) | 3.49 (0.58) | < 0.001 |
| Time perception altered | 3 | 46539 | 353 | 61689570 | 11.27 (3.62 - 35.1) | 11.26 (27.82) | 11.18 (3.59) | 3.48 (0.21) | < 0.001 |
| Application site irritation | 103 | 46439 | 12189 | 61677734 | 11.22 (9.24 - 13.63) | 11.2 (949.02) | 11.12 (9.15) | 3.47 (3.06) | < 0.001 |
| Electrocardiogram pr prolongation | 7 | 46535 | 834 | 61689089 | 11.13 (5.29 - 23.41) | 11.13 (63.97) | 11.04 (5.25) | 3.46 (1.27) | < 0.001 |
| Brain contusion | 6 | 46536 | 723 | 61689200 | 11 (4.93 - 24.57) | 11 (54.1) | 10.92 (4.89) | 3.45 (1.08) | < 0.001 |
| Apallic syndrome | 6 | 46536 | 731 | 61689192 | 10.88 (4.87 - 24.3) | 10.88 (53.39) | 10.8 (4.84) | 3.43 (1.07) | < 0.001 |
| Bronchopneumopathy | 3 | 46539 | 366 | 61689557 | 10.87 (3.49 - 33.85) | 10.86 (26.65) | 10.78 (3.46) | 3.43 (0.19) | < 0.001 |
| Brain hypoxia | 8 | 46534 | 993 | 61688930 | 10.68 (5.33 - 21.42) | 10.68 (69.62) | 10.6 (5.29) | 3.41 (1.39) | < 0.001 |
| Bradyarrhythmia | 13 | 46529 | 1618 | 61688305 | 10.65 (6.17 - 18.39) | 10.65 (112.76) | 10.57 (6.13) | 3.4 (1.88) | < 0.001 |
| Labyrinthitis | 20 | 46522 | 2509 | 61687414 | 10.57 (6.81 - 16.41) | 10.57 (171.84) | 10.49 (6.76) | 3.39 (2.22) | < 0.001 |
| Saliva altered | 7 | 46535 | 878 | 61689045 | 10.57 (5.02 - 22.24) | 10.57 (60.16) | 10.49 (4.99) | 3.39 (1.24) | < 0.001 |
| Daydreaming | 12 | 46530 | 1518 | 61688405 | 10.48 (5.94 - 18.5) | 10.48 (102.08) | 10.4 (5.89) | 3.38 (1.79) | < 0.001 |
| Impaired reasoning | 3 | 46539 | 380 | 61689543 | 10.46 (3.36 - 32.59) | 10.46 (25.48) | 10.39 (3.34) | 3.38 (0.18) | < 0.001 |
| Poverty of speech | 3 | 46539 | 384 | 61689539 | 10.36 (3.33 - 32.25) | 10.36 (25.16) | 10.28 (3.3) | 3.36 (0.18) | < 0.001 |
| Cerebral atrophy | 31 | 46511 | 4000 | 61685923 | 10.28 (7.22 - 14.64) | 10.27 (257.48) | 10.2 (7.16) | 3.35 (2.47) | < 0.001 |
| Application site swelling | 37 | 46505 | 4896 | 61685027 | 10.02 (7.25 - 13.85) | 10.02 (298.09) | 9.95 (7.2) | 3.31 (2.54) | < 0.001 |
| Application site erosion | 10 | 46532 | 1338 | 61688585 | 9.91 (5.32 - 18.46) | 9.91 (79.48) | 9.84 (5.28) | 3.3 (1.57) | < 0.001 |
| Application site laceration | 3 | 46539 | 403 | 61689520 | 9.87 (3.17 - 30.73) | 9.87 (23.73) | 9.8 (3.15) | 3.29 (0.16) | < 0.001 |
| Communication disorder | 28 | 46514 | 3777 | 61686146 | 9.83 (6.78 - 14.26) | 9.83 (220.36) | 9.76 (6.73) | 3.29 (2.37) | < 0.001 |
| Illusion | 12 | 46530 | 1663 | 61688260 | 9.57 (5.42 - 16.88) | 9.56 (91.37) | 9.5 (5.39) | 3.25 (1.72) | < 0.001 |
| Rapid eye movements sleep abnormal | 3 | 46539 | 416 | 61689507 | 9.56 (3.07 - 29.76) | 9.56 (22.83) | 9.5 (3.05) | 3.25 (0.15) | < 0.001 |
| Suture insertion | 4 | 46538 | 569 | 61689354 | 9.32 (3.49 - 24.92) | 9.32 (29.49) | 9.26 (3.46) | 3.21 (0.51) | < 0.001 |
| Aggression | 337 | 46205 | 49302 | 61640621 | 9.12 (8.19 - 10.15) | 9.06 (2401.99) | 9.01 (8.09) | 3.17 (2.98) | < 0.001 |
| Posture abnormal | 22 | 46520 | 3199 | 61686724 | 9.12 (6 - 13.87) | 9.12 (157.88) | 9.06 (5.96) | 3.18 (2.14) | < 0.001 |
| Stoma site inflammation | 5 | 46537 | 729 | 61689194 | 9.09 (3.77 - 21.91) | 9.09 (35.76) | 9.04 (3.75) | 3.18 (0.77) | < 0.001 |
| Bradykinesia | 39 | 46503 | 5718 | 61684205 | 9.05 (6.6 - 12.4) | 9.04 (277.03) | 8.99 (6.56) | 3.17 (2.45) | < 0.001 |
| Delusional disorder, unspecified type | 5 | 46537 | 735 | 61689188 | 9.02 (3.74 - 21.73) | 9.02 (35.4) | 8.96 (3.72) | 3.16 (0.76) | < 0.001 |
| Torticollis | 14 | 46528 | 2064 | 61687859 | 8.99 (5.32 - 15.21) | 8.99 (98.76) | 8.94 (5.28) | 3.16 (1.8) | < 0.001 |
| Negativism | 5 | 46537 | 748 | 61689175 | 8.86 (3.68 - 21.35) | 8.86 (34.63) | 8.81 (3.66) | 3.14 (0.75) | < 0.001 |
| Sexually inappropriate behaviour | 3 | 46539 | 450 | 61689473 | 8.84 (2.84 - 27.5) | 8.84 (20.71) | 8.78 (2.82) | 3.13 (0.13) | 0.001 |
| Application site vesicles | 42 | 46500 | 6397 | 61683526 | 8.71 (6.43 - 11.8) | 8.7 (284.49) | 8.65 (6.39) | 3.11 (2.43) | < 0.001 |
| Salivary hypersecretion | 64 | 46478 | 9822 | 61680101 | 8.65 (6.76 - 11.06) | 8.64 (429.43) | 8.59 (6.71) | 3.1 (2.58) | < 0.001 |
| Spinal column injury | 15 | 46527 | 2308 | 61687615 | 8.62 (5.19 - 14.32) | 8.61 (100.31) | 8.57 (5.15) | 3.1 (1.82) | < 0.001 |
| Application site acne | 3 | 46539 | 468 | 61689455 | 8.5 (2.73 - 26.44) | 8.5 (19.72) | 8.45 (2.71) | 3.08 (0.11) | 0.001 |
| Anticholinergic syndrome | 10 | 46532 | 1566 | 61688357 | 8.47 (4.55 - 15.77) | 8.46 (65.41) | 8.42 (4.52) | 3.07 (1.46) | < 0.001 |
| Ischaemia | 29 | 46513 | 4585 | 61685338 | 8.39 (5.82 - 12.09) | 8.38 (187.41) | 8.34 (5.79) | 3.06 (2.21) | < 0.001 |
| Freezing phenomenon | 22 | 46520 | 3532 | 61686391 | 8.26 (5.43 - 12.56) | 8.26 (139.44) | 8.21 (5.4) | 3.04 (2.04) | < 0.001 |
| Abnormal behaviour | 241 | 46301 | 39307 | 61650616 | 8.16 (7.19 - 9.27) | 8.13 (1497.98) | 8.08 (7.12) | 3.01 (2.79) | < 0.001 |
| Cerebral thrombosis | 17 | 46525 | 2780 | 61687143 | 8.11 (5.03 - 13.06) | 8.11 (105.25) | 8.06 (5) | 3.01 (1.85) | < 0.001 |
| Bladder obstruction | 3 | 46539 | 491 | 61689432 | 8.1 (2.6 - 25.2) | 8.1 (18.55) | 8.06 (2.59) | 3.01 (0.09) | 0.002 |
| Femoral neck fracture | 31 | 46511 | 5079 | 61684844 | 8.09 (5.69 - 11.52) | 8.09 (191.47) | 8.05 (5.65) | 3.01 (2.21) | < 0.001 |
| Stoma site reaction | 4 | 46538 | 665 | 61689258 | 7.97 (2.98 - 21.31) | 7.97 (24.25) | 7.93 (2.97) | 2.99 (0.44) | < 0.001 |
| Application site infection | 3 | 46539 | 504 | 61689419 | 7.89 (2.54 - 24.55) | 7.89 (17.94) | 7.85 (2.52) | 2.97 (0.08) | 0.002 |
| Neuropsychological symptoms | 9 | 46533 | 1524 | 61688399 | 7.83 (4.07 - 15.08) | 7.83 (53.28) | 7.79 (4.04) | 2.96 (1.3) | < 0.001 |
| Soliloquy | 5 | 46537 | 849 | 61689074 | 7.81 (3.24 - 18.81) | 7.81 (29.5) | 7.77 (3.22) | 2.96 (0.69) | < 0.001 |
| Bronchial disorder | 9 | 46533 | 1533 | 61688390 | 7.78 (4.04 - 14.99) | 7.78 (52.88) | 7.74 (4.02) | 2.95 (1.29) | < 0.001 |
| Hallucination | 428 | 46114 | 73644 | 61616279 | 7.77 (7.06 - 8.54) | 7.7 (2485.12) | 7.66 (6.97) | 2.94 (2.78) | < 0.001 |
| On and off phenomenon | 30 | 46512 | 5169 | 61684754 | 7.7 (5.38 - 11.02) | 7.69 (173.69) | 7.65 (5.35) | 2.94 (2.14) | < 0.001 |
| Bronchial obstruction | 8 | 46534 | 1392 | 61688531 | 7.62 (3.8 - 15.27) | 7.62 (45.73) | 7.58 (3.78) | 2.92 (1.16) | < 0.001 |
| Logorrhoea | 20 | 46522 | 3490 | 61686433 | 7.6 (4.9 - 11.79) | 7.6 (113.9) | 7.56 (4.87) | 2.92 (1.89) | < 0.001 |
| Atrophy | 11 | 46531 | 1936 | 61687987 | 7.53 (4.16 - 13.63) | 7.53 (61.95) | 7.49 (4.14) | 2.91 (1.45) | < 0.001 |
| Dermatitis contact | 63 | 46479 | 11193 | 61678730 | 7.47 (5.83 - 9.57) | 7.46 (350.54) | 7.42 (5.79) | 2.89 (2.39) | < 0.001 |
| Pulmonary sepsis | 8 | 46534 | 1427 | 61688496 | 7.43 (3.71 - 14.89) | 7.43 (44.28) | 7.39 (3.69) | 2.89 (1.15) | < 0.001 |
| Clavicle fracture | 21 | 46521 | 3778 | 61686145 | 7.37 (4.8 - 11.32) | 7.37 (114.94) | 7.33 (4.77) | 2.87 (1.89) | < 0.001 |
| Dyslalia | 4 | 46538 | 725 | 61689198 | 7.31 (2.74 - 19.54) | 7.31 (21.68) | 7.28 (2.72) | 2.86 (0.39) | < 0.001 |
| Hallucination, visual | 113 | 46429 | 20656 | 61669267 | 7.27 (6.04 - 8.74) | 7.25 (605.84) | 7.22 (6) | 2.85 (2.5) | < 0.001 |
| Moaning | 6 | 46536 | 1098 | 61688825 | 7.24 (3.25 - 16.16) | 7.24 (32.11) | 7.21 (3.23) | 2.85 (0.84) | < 0.001 |
| Stoma site erythema | 10 | 46532 | 1836 | 61688087 | 7.22 (3.88 - 13.44) | 7.22 (53.29) | 7.19 (3.86) | 2.85 (1.33) | < 0.001 |
| Eating disorder | 112 | 46430 | 21017 | 61668906 | 7.08 (5.88 - 8.52) | 7.06 (580.07) | 7.03 (5.84) | 2.81 (2.47) | < 0.001 |
| Hyporesponsive to stimuli | 6 | 46536 | 1129 | 61688794 | 7.04 (3.16 - 15.72) | 7.04 (30.95) | 7.01 (3.14) | 2.81 (0.82) | < 0.001 |
| Dengue fever | 12 | 46530 | 2263 | 61687660 | 7.03 (3.99 - 12.4) | 7.03 (61.72) | 7 (3.97) | 2.81 (1.46) | < 0.001 |
| Distractibility | 6 | 46536 | 1138 | 61688785 | 6.99 (3.13 - 15.59) | 6.99 (30.63) | 6.96 (3.12) | 2.8 (0.82) | < 0.001 |
| Sinus arrest | 8 | 46534 | 1523 | 61688400 | 6.96 (3.48 - 13.95) | 6.96 (40.64) | 6.93 (3.46) | 2.79 (1.1) | < 0.001 |
| Sputum retention | 5 | 46537 | 959 | 61688964 | 6.91 (2.87 - 16.64) | 6.91 (25.15) | 6.88 (2.86) | 2.78 (0.61) | < 0.001 |
| Muscle rigidity | 61 | 46481 | 11763 | 61678160 | 6.88 (5.35 - 8.85) | 6.87 (304.64) | 6.84 (5.32) | 2.77 (2.28) | < 0.001 |
| Underweight | 10 | 46532 | 1933 | 61687990 | 6.86 (3.68 - 12.77) | 6.86 (49.77) | 6.83 (3.67) | 2.77 (1.28) | < 0.001 |
| Femur fracture | 157 | 46385 | 30544 | 61659379 | 6.83 (5.84 - 8) | 6.81 (775.1) | 6.78 (5.8) | 2.76 (2.48) | < 0.001 |
| Delirium | 174 | 46368 | 34061 | 61655862 | 6.79 (5.85 - 7.89) | 6.77 (852) | 6.74 (5.81) | 2.75 (2.49) | < 0.001 |
| Application site haemorrhage | 15 | 46527 | 2960 | 61686963 | 6.72 (4.04 - 11.16) | 6.72 (72.62) | 6.69 (4.03) | 2.74 (1.58) | < 0.001 |
| Sinus node dysfunction | 15 | 46527 | 2991 | 61686932 | 6.65 (4 - 11.04) | 6.65 (71.61) | 6.62 (3.99) | 2.73 (1.57) | < 0.001 |
| Atrioventricular block second degree | 15 | 46527 | 3007 | 61686916 | 6.61 (3.98 - 10.99) | 6.61 (71.1) | 6.58 (3.96) | 2.72 (1.56) | < 0.001 |
| Incorrect product dosage form administered | 5 | 46537 | 1006 | 61688917 | 6.59 (2.74 - 15.86) | 6.59 (23.58) | 6.56 (2.72) | 2.71 (0.59) | < 0.001 |
| Agitation | 358 | 46184 | 72824 | 61617099 | 6.56 (5.91 - 7.28) | 6.52 (1665.45) | 6.49 (5.85) | 2.7 (2.52) | < 0.001 |
| Atrioventricular block complete | 33 | 46509 | 6696 | 61683227 | 6.54 (4.64 - 9.2) | 6.53 (153.88) | 6.51 (4.62) | 2.7 (1.99) | < 0.001 |
| Hyperkinesia | 10 | 46532 | 2058 | 61687865 | 6.44 (3.46 - 11.99) | 6.44 (45.74) | 6.41 (3.45) | 2.68 (1.23) | < 0.001 |
| Food refusal | 4 | 46538 | 829 | 61689094 | 6.4 (2.39 - 17.08) | 6.4 (18.12) | 6.37 (2.38) | 2.67 (0.32) | < 0.001 |
| Speech disorder | 246 | 46296 | 51614 | 61638309 | 6.35 (5.6 - 7.19) | 6.32 (1096.71) | 6.29 (5.55) | 2.65 (2.44) | < 0.001 |
| Tongue movement disturbance | 4 | 46538 | 843 | 61689080 | 6.29 (2.36 - 16.8) | 6.29 (17.71) | 6.26 (2.35) | 2.65 (0.31) | < 0.001 |
| Slow response to stimuli | 6 | 46536 | 1271 | 61688652 | 6.26 (2.81 - 13.96) | 6.26 (26.38) | 6.23 (2.79) | 2.64 (0.74) | < 0.001 |
| Decubitus ulcer | 38 | 46504 | 8084 | 61681839 | 6.23 (4.53 - 8.58) | 6.23 (166.1) | 6.21 (4.51) | 2.63 (1.99) | < 0.001 |
| Hydrocephalus | 25 | 46517 | 5358 | 61684565 | 6.19 (4.18 - 9.17) | 6.18 (108.16) | 6.16 (4.16) | 2.62 (1.79) | < 0.001 |
| Aspiration | 50 | 46492 | 10737 | 61679186 | 6.18 (4.68 - 8.16) | 6.17 (215.76) | 6.15 (4.66) | 2.62 (2.08) | < 0.001 |
| Hypokinesia | 66 | 46476 | 14331 | 61675592 | 6.11 (4.8 - 7.78) | 6.1 (280.47) | 6.08 (4.77) | 2.6 (2.15) | < 0.001 |
| Incoherent | 29 | 46513 | 6417 | 61683506 | 5.99 (4.16 - 8.63) | 5.99 (120.02) | 5.97 (4.14) | 2.58 (1.83) | < 0.001 |
| Application site urticaria | 10 | 46532 | 2226 | 61687697 | 5.96 (3.2 - 11.08) | 5.95 (41.04) | 5.93 (3.19) | 2.57 (1.16) | < 0.001 |
| Gastrointestinal tube insertion | 11 | 46531 | 2455 | 61687468 | 5.94 (3.29 - 10.74) | 5.94 (44.98) | 5.92 (3.27) | 2.56 (1.23) | < 0.001 |
| Miosis | 35 | 46507 | 7845 | 61682078 | 5.92 (4.24 - 8.25) | 5.91 (142.27) | 5.89 (4.23) | 2.56 (1.89) | < 0.001 |
| Intestinal pseudo-obstruction | 5 | 46537 | 1127 | 61688796 | 5.88 (2.44 - 14.16) | 5.88 (20.16) | 5.86 (2.43) | 2.55 (0.51) | < 0.001 |
| Walking disability | 9 | 46533 | 2058 | 61687865 | 5.8 (3.01 - 11.16) | 5.8 (35.57) | 5.78 (3) | 2.53 (1.05) | < 0.001 |
| Subileus | 9 | 46533 | 2065 | 61687858 | 5.78 (3 - 11.12) | 5.78 (35.4) | 5.76 (2.99) | 2.53 (1.05) | < 0.001 |
| Increased bronchial secretion | 11 | 46531 | 2527 | 61687396 | 5.77 (3.19 - 10.43) | 5.77 (43.19) | 5.75 (3.18) | 2.52 (1.21) | < 0.001 |
| Screaming | 27 | 46515 | 6316 | 61683607 | 5.67 (3.88 - 8.27) | 5.67 (103.32) | 5.65 (3.87) | 2.5 (1.73) | < 0.001 |
| Bundle branch block | 4 | 46538 | 937 | 61688986 | 5.66 (2.12 - 15.11) | 5.66 (15.28) | 5.64 (2.11) | 2.5 (0.25) | 0.002 |
| Hypervigilance | 4 | 46538 | 945 | 61688978 | 5.61 (2.1 - 14.98) | 5.61 (15.09) | 5.59 (2.09) | 2.48 (0.25) | 0.002 |
| Delusion | 64 | 46478 | 15293 | 61674630 | 5.55 (4.34 - 7.1) | 5.55 (237.61) | 5.53 (4.32) | 2.47 (2.01) | < 0.001 |
| Bladder catheterisation | 4 | 46538 | 959 | 61688964 | 5.53 (2.07 - 14.76) | 5.53 (14.78) | 5.51 (2.06) | 2.46 (0.24) | 0.002 |
| Lacunar infarction | 10 | 46532 | 2419 | 61687504 | 5.48 (2.94 - 10.2) | 5.48 (36.47) | 5.46 (2.93) | 2.45 (1.09) | < 0.001 |
| Bradycardia | 215 | 46327 | 52462 | 61637461 | 5.45 (4.77 - 6.24) | 5.43 (774.96) | 5.41 (4.73) | 2.44 (2.21) | < 0.001 |
| Stoma site discharge | 11 | 46531 | 2695 | 61687228 | 5.41 (2.99 - 9.78) | 5.41 (39.39) | 5.39 (2.98) | 2.43 (1.15) | < 0.001 |
| Cerebral ischaemia | 21 | 46521 | 5173 | 61684750 | 5.38 (3.51 - 8.26) | 5.38 (74.6) | 5.36 (3.49) | 2.42 (1.55) | < 0.001 |
| Rhinitis allergic | 18 | 46524 | 4484 | 61685439 | 5.32 (3.35 - 8.46) | 5.32 (62.91) | 5.3 (3.34) | 2.41 (1.45) | < 0.001 |
| Hepatic infection | 5 | 46537 | 1248 | 61688675 | 5.31 (2.21 - 12.78) | 5.31 (17.42) | 5.29 (2.2) | 2.4 (0.44) | < 0.001 |
| Muscle contracture | 8 | 46534 | 2018 | 61687905 | 5.26 (2.62 - 10.52) | 5.25 (27.45) | 5.24 (2.62) | 2.39 (0.87) | < 0.001 |
| Parkinsonism | 38 | 46504 | 9596 | 61680327 | 5.25 (3.82 - 7.22) | 5.25 (130.2) | 5.23 (3.8) | 2.39 (1.78) | < 0.001 |
| Body height decreased | 42 | 46500 | 10640 | 61679283 | 5.24 (3.87 - 7.09) | 5.23 (143.24) | 5.22 (3.85) | 2.38 (1.81) | < 0.001 |
| Atrioventricular block | 30 | 46512 | 7623 | 61682300 | 5.22 (3.65 - 7.47) | 5.22 (101.85) | 5.2 (3.63) | 2.38 (1.68) | < 0.001 |
| Fracture | 73 | 46469 | 18590 | 61671333 | 5.21 (4.14 - 6.56) | 5.2 (247.09) | 5.19 (4.12) | 2.38 (1.96) | < 0.001 |
| Venous occlusion | 7 | 46535 | 1796 | 61688127 | 5.17 (2.46 - 10.85) | 5.17 (23.43) | 5.15 (2.45) | 2.36 (0.74) | < 0.001 |
| Hemiplegia | 32 | 46510 | 8271 | 61681652 | 5.13 (3.63 - 7.26) | 5.13 (105.95) | 5.11 (3.61) | 2.35 (1.68) | < 0.001 |
| Confusional state | 610 | 45932 | 159619 | 61530304 | 5.12 (4.73 - 5.55) | 5.07 (1987.91) | 5.05 (4.66) | 2.34 (2.21) | < 0.001 |
| Aphasia | 117 | 46425 | 30324 | 61659599 | 5.12 (4.27 - 6.15) | 5.11 (385.93) | 5.1 (4.25) | 2.35 (2.03) | < 0.001 |
| Apathy | 55 | 46487 | 14317 | 61675606 | 5.1 (3.91 - 6.64) | 5.09 (180.21) | 5.08 (3.89) | 2.34 (1.86) | < 0.001 |
| Sinus bradycardia | 36 | 46506 | 9605 | 61680318 | 4.97 (3.58 - 6.9) | 4.97 (113.68) | 4.95 (3.57) | 2.31 (1.69) | < 0.001 |
| Fluid intake reduced | 18 | 46524 | 4868 | 61685055 | 4.9 (3.09 - 7.79) | 4.9 (55.69) | 4.89 (3.08) | 2.29 (1.36) | < 0.001 |
| Feeding disorder | 72 | 46470 | 19612 | 61670311 | 4.87 (3.86 - 6.14) | 4.87 (220.42) | 4.85 (3.85) | 2.28 (1.87) | < 0.001 |
| Hyporeflexia | 7 | 46535 | 1919 | 61688004 | 4.84 (2.3 - 10.16) | 4.83 (21.22) | 4.82 (2.3) | 2.27 (0.68) | < 0.001 |
| Food aversion | 6 | 46536 | 1643 | 61688280 | 4.84 (2.17 - 10.79) | 4.84 (18.22) | 4.83 (2.17) | 2.27 (0.55) | < 0.001 |
| Restlessness | 131 | 46411 | 36168 | 61653755 | 4.81 (4.05 - 5.71) | 4.8 (393) | 4.79 (4.03) | 2.26 (1.97) | < 0.001 |
| Miliaria | 7 | 46535 | 1934 | 61687989 | 4.8 (2.28 - 10.08) | 4.8 (20.97) | 4.78 (2.28) | 2.26 (0.68) | < 0.001 |
| Gait inability | 206 | 46336 | 57275 | 61632648 | 4.78 (4.17 - 5.49) | 4.77 (611.64) | 4.75 (4.14) | 2.25 (2.02) | < 0.001 |
| Spinal deformity | 6 | 46536 | 1671 | 61688252 | 4.76 (2.14 - 10.61) | 4.76 (17.75) | 4.75 (2.13) | 2.25 (0.53) | < 0.001 |
| Nasal polyps | 17 | 46525 | 4752 | 61685171 | 4.74 (2.95 - 7.64) | 4.74 (50.02) | 4.73 (2.94) | 2.24 (1.29) | < 0.001 |
| Bundle branch block left | 15 | 46527 | 4243 | 61685680 | 4.69 (2.82 - 7.78) | 4.69 (43.34) | 4.67 (2.81) | 2.22 (1.2) | < 0.001 |
| Skin reaction | 49 | 46493 | 13880 | 61676043 | 4.68 (3.54 - 6.2) | 4.68 (141.29) | 4.67 (3.52) | 2.22 (1.71) | < 0.001 |
| Catatonia | 18 | 46524 | 5137 | 61684786 | 4.65 (2.92 - 7.38) | 4.64 (51.3) | 4.63 (2.92) | 2.21 (1.3) | < 0.001 |
| Psychotic symptom | 7 | 46535 | 2006 | 61687917 | 4.63 (2.2 - 9.72) | 4.63 (19.82) | 4.61 (2.2) | 2.21 (0.65) | < 0.001 |
| Kyphosis | 6 | 46536 | 1764 | 61688159 | 4.51 (2.02 - 10.05) | 4.51 (16.33) | 4.5 (2.02) | 2.17 (0.49) | < 0.001 |
| Cardiopulmonary failure | 13 | 46529 | 3901 | 61686022 | 4.42 (2.56 - 7.62) | 4.42 (34.25) | 4.41 (2.56) | 2.14 (1.05) | < 0.001 |
| Terminal state | 22 | 46520 | 6622 | 61683301 | 4.41 (2.9 - 6.7) | 4.4 (57.69) | 4.39 (2.89) | 2.13 (1.33) | < 0.001 |
| Cardio-respiratory arrest | 133 | 46409 | 41704 | 61648219 | 4.24 (3.57 - 5.02) | 4.23 (326.85) | 4.22 (3.56) | 2.08 (1.79) | < 0.001 |
| Diabetic complication | 8 | 46534 | 2532 | 61687391 | 4.19 (2.09 - 8.39) | 4.19 (19.35) | 4.18 (2.09) | 2.06 (0.66) | < 0.001 |
| Dysstasia | 91 | 46451 | 28884 | 61661039 | 4.18 (3.4 - 5.14) | 4.18 (219.21) | 4.17 (3.39) | 2.06 (1.71) | < 0.001 |
| Muscle contractions involuntary | 11 | 46531 | 3490 | 61686433 | 4.18 (2.31 - 7.55) | 4.18 (26.51) | 4.17 (2.31) | 2.06 (0.89) | < 0.001 |
| Autonomic nervous system imbalance | 9 | 46533 | 2855 | 61687068 | 4.18 (2.17 - 8.04) | 4.18 (21.69) | 4.17 (2.17) | 2.06 (0.75) | < 0.001 |
| Urinary incontinence | 92 | 46450 | 29283 | 61660640 | 4.17 (3.4 - 5.12) | 4.16 (220.62) | 4.15 (3.38) | 2.05 (1.71) | < 0.001 |
| Altered state of consciousness | 65 | 46477 | 21282 | 61668641 | 4.05 (3.18 - 5.17) | 4.05 (148.79) | 4.04 (3.17) | 2.01 (1.59) | < 0.001 |
| Syncope | 301 | 46241 | 99670 | 61590253 | 4.02 (3.59 - 4.51) | 4 (677.11) | 3.99 (3.57) | 2 (1.82) | < 0.001 |
| Motor dysfunction | 30 | 46512 | 9954 | 61679969 | 4 (2.79 - 5.72) | 3.99 (67.16) | 3.99 (2.78) | 1.99 (1.34) | < 0.001 |
| Pemphigoid | 22 | 46520 | 7295 | 61682628 | 4 (2.63 - 6.08) | 4 (49.3) | 3.99 (2.62) | 2 (1.22) | < 0.001 |
| Dermatitis bullous | 21 | 46521 | 7103 | 61682820 | 3.92 (2.55 - 6.02) | 3.92 (45.52) | 3.91 (2.55) | 1.97 (1.17) | < 0.001 |
| Behaviour disorder | 16 | 46526 | 5427 | 61684496 | 3.91 (2.39 - 6.39) | 3.91 (34.52) | 3.9 (2.39) | 1.96 (1.03) | < 0.001 |
| Malnutrition | 32 | 46510 | 11084 | 61678839 | 3.83 (2.71 - 5.42) | 3.83 (66.64) | 3.82 (2.7) | 1.93 (1.31) | < 0.001 |
| Anal incontinence | 38 | 46504 | 13602 | 61676321 | 3.71 (2.69 - 5.09) | 3.7 (74.78) | 3.7 (2.69) | 1.89 (1.33) | < 0.001 |
| Choking | 53 | 46489 | 19158 | 61670765 | 3.67 (2.8 - 4.81) | 3.67 (102.55) | 3.66 (2.79) | 1.87 (1.41) | < 0.001 |
| Vitamin b12 deficiency | 11 | 46531 | 3986 | 61685937 | 3.66 (2.02 - 6.61) | 3.66 (21.19) | 3.65 (2.02) | 1.87 (0.75) | < 0.001 |
| Dysphagia | 267 | 46275 | 97938 | 61591985 | 3.63 (3.22 - 4.09) | 3.61 (504.13) | 3.61 (3.2) | 1.85 (1.66) | < 0.001 |
| Arrhythmia | 128 | 46414 | 46955 | 61642968 | 3.62 (3.04 - 4.31) | 3.61 (241.45) | 3.61 (3.03) | 1.85 (1.57) | < 0.001 |
| Cerebral infarction | 65 | 46477 | 24248 | 61665675 | 3.56 (2.79 - 4.54) | 3.55 (118.97) | 3.55 (2.78) | 1.83 (1.42) | < 0.001 |
| Circulatory collapse | 45 | 46497 | 16919 | 61673004 | 3.53 (2.63 - 4.73) | 3.53 (81.21) | 3.52 (2.63) | 1.82 (1.31) | < 0.001 |
| Scratch | 27 | 46515 | 10202 | 61679721 | 3.51 (2.41 - 5.12) | 3.51 (48.29) | 3.5 (2.4) | 1.81 (1.14) | < 0.001 |
| Hypertensive crisis | 29 | 46513 | 11029 | 61678894 | 3.49 (2.42 - 5.02) | 3.49 (51.27) | 3.48 (2.42) | 1.8 (1.16) | < 0.001 |
| Cerebrovascular accident | 437 | 46105 | 167523 | 61522400 | 3.48 (3.17 - 3.83) | 3.46 (763.45) | 3.45 (3.14) | 1.79 (1.64) | < 0.001 |
| Movement disorder | 82 | 46460 | 31793 | 61658130 | 3.42 (2.76 - 4.25) | 3.42 (140.02) | 3.41 (2.75) | 1.77 (1.41) | < 0.001 |
| Depressed level of consciousness | 100 | 46442 | 39163 | 61650760 | 3.39 (2.79 - 4.13) | 3.38 (167.67) | 3.38 (2.78) | 1.76 (1.43) | < 0.001 |
| Orthostatic hypotension | 44 | 46498 | 17267 | 61672656 | 3.38 (2.51 - 4.54) | 3.38 (73.47) | 3.37 (2.51) | 1.75 (1.25) | < 0.001 |
| Respiratory arrest | 69 | 46473 | 27566 | 61662357 | 3.32 (2.62 - 4.21) | 3.32 (111.49) | 3.31 (2.61) | 1.73 (1.34) | < 0.001 |
| Drooling | 17 | 46525 | 6788 | 61683135 | 3.32 (2.06 - 5.34) | 3.32 (27.49) | 3.31 (2.06) | 1.73 (0.87) | < 0.001 |
| Bedridden | 32 | 46510 | 12947 | 61676976 | 3.28 (2.32 - 4.64) | 3.28 (50.49) | 3.27 (2.31) | 1.71 (1.11) | < 0.001 |
| Fall | 799 | 45743 | 328170 | 61361753 | 3.27 (3.05 - 3.5) | 3.23 (1231.65) | 3.22 (3) | 1.69 (1.58) | < 0.001 |
| Incontinence | 25 | 46517 | 10473 | 61679450 | 3.17 (2.14 - 4.69) | 3.16 (36.92) | 3.16 (2.13) | 1.66 (0.98) | < 0.001 |
| Psychomotor hyperactivity | 39 | 46503 | 16496 | 61673427 | 3.14 (2.29 - 4.29) | 3.13 (56.54) | 3.13 (2.28) | 1.65 (1.11) | < 0.001 |
| General physical health deterioration | 266 | 46276 | 113684 | 61576239 | 3.11 (2.76 - 3.51) | 3.1 (378.54) | 3.1 (2.74) | 1.63 (1.44) | < 0.001 |
| Skin irritation | 106 | 46436 | 45202 | 61644721 | 3.11 (2.57 - 3.77) | 3.11 (151.33) | 3.1 (2.56) | 1.63 (1.33) | < 0.001 |
| Hypophagia | 65 | 46477 | 28031 | 61661892 | 3.08 (2.41 - 3.92) | 3.07 (90.76) | 3.07 (2.41) | 1.62 (1.22) | < 0.001 |
| Head injury | 72 | 46470 | 31199 | 61658724 | 3.06 (2.43 - 3.86) | 3.06 (99.6) | 3.05 (2.42) | 1.61 (1.23) | < 0.001 |
| Application site pain | 50 | 46492 | 21770 | 61668153 | 3.05 (2.31 - 4.02) | 3.04 (68.5) | 3.04 (2.3) | 1.6 (1.14) | < 0.001 |
| Decreased activity | 24 | 46518 | 10557 | 61679366 | 3.01 (2.02 - 4.5) | 3.01 (32.22) | 3.01 (2.02) | 1.59 (0.9) | < 0.001 |
| Paranoia | 38 | 46504 | 17110 | 61672813 | 2.95 (2.14 - 4.05) | 2.94 (48.68) | 2.94 (2.14) | 1.56 (1.02) | < 0.001 |
| Cardiac arrest | 180 | 46362 | 82178 | 61607745 | 2.91 (2.51 - 3.37) | 2.9 (224.39) | 2.9 (2.5) | 1.54 (1.31) | < 0.001 |
| Retching | 46 | 46496 | 20976 | 61668947 | 2.91 (2.18 - 3.88) | 2.91 (57.43) | 2.9 (2.17) | 1.54 (1.06) | < 0.001 |
| Pallor | 59 | 46483 | 26976 | 61662947 | 2.9 (2.25 - 3.75) | 2.9 (73.26) | 2.89 (2.24) | 1.53 (1.12) | < 0.001 |
| Dystonia | 42 | 46500 | 19199 | 61670724 | 2.9 (2.14 - 3.93) | 2.9 (52.17) | 2.9 (2.14) | 1.53 (1.03) | < 0.001 |
| Haematemesis | 53 | 46489 | 25114 | 61664809 | 2.8 (2.14 - 3.67) | 2.8 (61.1) | 2.79 (2.13) | 1.48 (1.04) | < 0.001 |
| Nervousness | 109 | 46433 | 51819 | 61638104 | 2.79 (2.31 - 3.37) | 2.79 (124.84) | 2.78 (2.31) | 1.48 (1.18) | < 0.001 |
| Multiple organ dysfunction syndrome | 94 | 46448 | 45136 | 61644787 | 2.76 (2.26 - 3.38) | 2.76 (105.39) | 2.76 (2.25) | 1.46 (1.14) | < 0.001 |
| Unresponsive to stimuli | 52 | 46490 | 25797 | 61664126 | 2.67 (2.04 - 3.51) | 2.67 (54.31) | 2.67 (2.03) | 1.42 (0.97) | < 0.001 |
| Loss of consciousness | 251 | 46291 | 126659 | 61563264 | 2.64 (2.33 - 2.98) | 2.63 (252.87) | 2.62 (2.32) | 1.39 (1.2) | < 0.001 |
| Nightmare | 68 | 46474 | 34295 | 61655628 | 2.63 (2.07 - 3.34) | 2.63 (68.49) | 2.62 (2.07) | 1.39 (1.01) | < 0.001 |
| Hip fracture | 62 | 46480 | 31656 | 61658267 | 2.6 (2.02 - 3.33) | 2.6 (60.75) | 2.59 (2.02) | 1.37 (0.97) | < 0.001 |
| Cardiac failure | 155 | 46387 | 79496 | 61610427 | 2.59 (2.21 - 3.03) | 2.58 (150.46) | 2.58 (2.2) | 1.37 (1.12) | < 0.001 |
| Gait disturbance | 360 | 46182 | 192724 | 61497199 | 2.49 (2.24 - 2.76) | 2.48 (317.13) | 2.47 (2.23) | 1.31 (1.15) | < 0.001 |
| Tremor | 304 | 46238 | 164062 | 61525861 | 2.47 (2.2 - 2.76) | 2.46 (262.63) | 2.45 (2.19) | 1.29 (1.12) | < 0.001 |
| Urinary tract infection | 303 | 46239 | 168086 | 61521837 | 2.4 (2.14 - 2.69) | 2.39 (245.01) | 2.39 (2.13) | 1.26 (1.08) | < 0.001 |
| Vomiting | 768 | 45774 | 462896 | 61227027 | 2.22 (2.07 - 2.38) | 2.2 (505.11) | 2.2 (2.05) | 1.14 (1.03) | < 0.001 |

Supplementary Table S15. Matrix heatmap of PT-level reporting signals and corresponding ROR (95% CI) across seven anti-Alzheimer's disease drugs.

| PT | Aducanumab | Donanemab | Donepezil | Galantamine | Memantine | Rivastigmine | Lecanemab |
| --- | --- | --- | --- | --- | --- | --- | --- |
| ARIA-E | 9731.43 (8072.48 - 11731.3) | 7621.95 (6737.22 - 8622.86) |  |  |  |  | 7297.37 (6460.08 - 8243.18) |
| ARIA-H | 7419.77 (5981.2 - 9204.33) | 6834.91 (5980.82 - 7810.97) |  |  |  |  | 8310.14 (7295.56 - 9465.83) |
| Headache | 3.81 (2.79 - 5.2) | 4.29 (3.79 - 4.86) | 0.66 (0.57 - 0.76) | 0.78 (0.57 - 1.06) | 1.23 (1.09 - 1.38) | 0.47 (0.41 - 0.54) | 6.83 (6.23 - 7.5) |
| Confusional state | 12.57 (8.98 - 17.61) | 5.51 (4.47 - 6.8) | 6.3 (5.73 - 6.94) | 3.86 (2.94 - 5.08) | 15.91 (14.87 - 17.03) | 5.12 (4.73 - 5.55) | 8.84 (7.58 - 10.31) |
| Cerebral haemorrhage | 43.66 (29.8 - 63.97) | 46.74 (39.98 - 54.66) | 2.3 (1.65 - 3.21) | 5.69 (3.53 - 9.16) | 1.18 (0.71 - 1.95) | 1.99 (1.52 - 2.6) | 28.11 (23.41 - 33.75) |
| Superficial siderosis of central nervous system | 14264.16 (8997.56 - 22613.48) | 1606.54 (946.85 - 2725.85) |  |  |  |  | 5602.81 (3855.45 - 8142.11) |
| Fall | 3.97 (2.63 - 6) | 2.37 (1.89 - 2.96) | 3.46 (3.16 - 3.79) | 3.87 (3.19 - 4.69) | 3.81 (3.47 - 4.19) | 3.27 (3.05 - 3.5) | 2.66 (2.2 - 3.23) |
| Seizure | 6.63 (4.26 - 10.32) | 2.62 (1.95 - 3.51) | 3.11 (2.73 - 3.54) | 2.98 (2.21 - 4.03) | 3.41 (2.97 - 3.9) | 1.51 (1.32 - 1.74) | 2.65 (2.03 - 3.46) |
| Amyloid related imaging abnormalities | 3782.79 (2306.93 - 6202.82) | 13470.47 (10581.91 - 17147.52) |  |  |  |  | 4322.55 (3358.88 - 5562.72) |
| Brain oedema | 63.2 (37.29 - 107.1) | 55.43 (43.69 - 70.32) | 0.74 (0.28 - 1.98) |  | 3.33 (2.01 - 5.53) | 1.59 (0.96 - 2.63) | 20.09 (14.03 - 28.77) |
| Atrial fibrillation | 6.41 (3.54 - 11.61) | 1.13 (0.62 - 2.04) | 2.49 (2.05 - 3.02) | 1.59 (0.92 - 2.74) | 0.95 (0.67 - 1.34) | 1.06 (0.84 - 1.32) | 3.9 (2.91 - 5.23) |
| Subarachnoid haemorrhage | 50.08 (25.98 - 96.53) | 7.84 (3.92 - 15.7) | 1.84 (0.92 - 3.68) | 10.52 (5.47 - 20.23) | 1.1 (0.41 - 2.93) | 1.31 (0.7 - 2.44) | 9.09 (5.03 - 16.43) |
| Head injury | 14.39 (7.18 - 28.86) | 5.72 (3.6 - 9.09) | 2.09 (1.44 - 3.02) | 0.38 (0.05 - 2.68) | 2.05 (1.36 - 3.08) | 3.06 (2.43 - 3.86) | 3.48 (2.02 - 5.99) |
| Cerebral microhaemorrhage | 974.19 (460.39 - 2061.42) | 2739.99 (2161.81 - 3472.81) |  |  |  |  | 589.39 (398.69 - 871.31) |
| Mental status changes | 10.16 (4.22 - 24.45) | 1.8 (0.75 - 4.32) | 4.14 (3.13 - 5.48) | 2.57 (1.15 - 5.72) | 7.49 (5.96 - 9.41) | 1.39 (0.97 - 2.01) | 3.63 (2.06 - 6.4) |
| Transient ischaemic attack | 8.33 (3.46 - 20.06) | 3.24 (1.8 - 5.86) | 0.83 (0.47 - 1.46) | 2.11 (0.95 - 4.69) | 1.65 (1.07 - 2.56) | 2.25 (1.73 - 2.92) | 2.98 (1.69 - 5.25) |
| Aphasia | 7.37 (2.76 - 19.66) | 5.55 (3.45 - 8.94) | 2.45 (1.73 - 3.47) | 6.23 (3.81 - 10.17) | 5.6 (4.35 - 7.2) | 5.12 (4.27 - 6.15) | 6.33 (4.2 - 9.54) |
| Posterior reversible encephalopathy syndrome | 16.5 (5.31 - 51.26) | 2.92 (0.94 - 9.06) |  |  |  | 0.13 (0.02 - 0.92) | 0.82 (0.12 - 5.82) |
| Subdural haematoma | 11.8 (3.8 - 36.64) | 15.39 (10.12 - 23.39) | 2.29 (1.36 - 3.87) | 4.15 (1.73 - 9.98) | 1.37 (0.65 - 2.87) | 2.14 (1.42 - 3.23) | 16.51 (11.39 - 23.93) |
| Haemorrhage intracranial | 11.31 (3.64 - 35.14) | 1.34 (0.33 - 5.34) | 0.16 (0.02 - 1.11) | 0.8 (0.11 - 5.65) | 0.56 (0.18 - 1.74) | 0.27 (0.09 - 0.83) | 1.69 (0.54 - 5.23) |
| Ischaemic stroke | 9.5 (3.06 - 29.51) | 12.96 (8.6 - 19.52) | 0.53 (0.2 - 1.4) |  | 0.31 (0.08 - 1.26) | 1.43 (0.91 - 2.24) | 6.63 (3.92 - 11.2) |
| Skin cancer | 7.91 (2.55 - 24.56) |  | 0.11 (0.02 - 0.78) |  |  | 0.69 (0.38 - 1.24) |  |
| Cerebral infarction | 6.91 (2.23 - 21.46) | 13.13 (9.27 - 18.59) | 3.26 (2.33 - 4.57) | 8.78 (5.53 - 13.95) | 1.95 (1.21 - 3.13) | 3.56 (2.79 - 4.54) | 27.16 (21.72 - 33.96) |
| Infusion related reaction | 3.43 (1.29 - 9.16) | 55.2 (49.48 - 61.57) |  |  | 0.04 (0.01 - 0.3) |  | 42.4 (37.87 - 47.47) |
| Flushing |  | 19.16 (16.58 - 22.15) | 0.12 (0.05 - 0.28) | 0.94 (0.47 - 1.87) | 0.44 (0.27 - 0.72) | 0.13 (0.07 - 0.24) | 1.16 (0.69 - 1.96) |
| Dizziness | 1.94 (1.2 - 3.14) | 2.49 (2.08 - 2.98) | 1.8 (1.63 - 1.99) | 1.54 (1.21 - 1.97) | 4.24 (3.94 - 4.57) | 1.43 (1.32 - 1.56) | 2.84 (2.44 - 3.32) |
| Chills |  | 8.14 (6.64 - 9.98) | 0.34 (0.21 - 0.55) | 0.1 (0.01 - 0.72) | 0.65 (0.44 - 0.94) | 0.42 (0.31 - 0.58) | 18.08 (15.91 - 20.55) |
| Blood pressure increased | 1.11 (0.36 - 3.44) | 5.23 (4.19 - 6.53) | 0.68 (0.5 - 0.91) | 0.86 (0.47 - 1.55) | 1.62 (1.31 - 2) | 1.41 (1.21 - 1.65) | 1.99 (1.43 - 2.76) |
| Back pain | 0.24 (0.03 - 1.72) | 3.22 (2.56 - 4.05) | 0.29 (0.2 - 0.42) | 0.26 (0.11 - 0.62) | 0.6 (0.46 - 0.8) | 0.25 (0.19 - 0.34) | 1.46 (1.07 - 1.99) |
| Chest discomfort |  | 7.25 (5.76 - 9.14) | 0.46 (0.3 - 0.71) | 0.35 (0.11 - 1.09) | 0.28 (0.15 - 0.51) | 0.37 (0.25 - 0.53) | 0.66 (0.33 - 1.32) |
| Erythema | 0.27 (0.04 - 1.9) | 3.36 (2.66 - 4.25) | 0.35 (0.24 - 0.49) | 0.28 (0.12 - 0.68) | 0.31 (0.2 - 0.46) | 1.46 (1.28 - 1.66) | 0.76 (0.49 - 1.19) |
| Cerebrovascular accident | 2.34 (1.11 - 4.91) | 3.75 (2.92 - 4.8) | 0.79 (0.61 - 1.02) | 0.91 (0.53 - 1.58) | 0.91 (0.7 - 1.19) | 3.48 (3.17 - 3.83) | 2.3 (1.72 - 3.07) |
| Tremor | 1.7 (0.71 - 4.1) | 3.28 (2.51 - 4.29) | 2.59 (2.24 - 3) | 2.6 (1.87 - 3.61) | 2.58 (2.2 - 3.03) | 2.47 (2.2 - 2.76) | 4.67 (3.8 - 5.75) |
| Anaphylactic reaction |  | 9.2 (6.96 - 12.15) | 0.13 (0.04 - 0.4) |  | 0.31 (0.14 - 0.68) | 0.1 (0.04 - 0.26) | 0.77 (0.32 - 1.85) |
| Blood pressure decreased | 0.85 (0.12 - 6.04) | 6.53 (4.84 - 8.82) | 2.02 (1.56 - 2.62) | 1.98 (1.1 - 3.58) | 1.19 (0.82 - 1.72) | 2.21 (1.83 - 2.66) | 1.4 (0.77 - 2.53) |
| Feeling hot |  | 6.74 (4.96 - 9.17) | 0.38 (0.21 - 0.71) | 0.19 (0.03 - 1.38) | 0.55 (0.31 - 0.97) | 0.35 (0.21 - 0.57) | 1.38 (0.74 - 2.56) |
| Infusion related hypersensitivity reaction |  | 552.47 (382.19 - 798.61) |  |  |  |  |  |
| Anaphylactic shock |  | 11.39 (7.91 - 16.4) | 0.09 (0.01 - 0.65) | 0.47 (0.07 - 3.3) |  | 0.26 (0.11 - 0.63) |  |
| Oxygen saturation decreased |  | 5.05 (3.48 - 7.32) | 0.25 (0.11 - 0.56) |  | 0.81 (0.49 - 1.32) | 0.89 (0.64 - 1.23) | 0.3 (0.08 - 1.21) |
| Feeling cold |  | 6.85 (4.37 - 10.75) | 0.42 (0.18 - 1.01) | 1.28 (0.41 - 3.99) | 0.81 (0.4 - 1.61) | 0.96 (0.62 - 1.49) | 12.8 (9.45 - 17.34) |
| Bradycardia | 2.12 (0.53 - 8.5) | 2.07 (1.15 - 3.75) | 34.76 (32.31 - 37.39) | 13.84 (10.75 - 17.82) | 9.71 (8.39 - 11.24) | 5.45 (4.77 - 6.24) | 1.9 (1.08 - 3.36) |
| Syncope | 2.24 (0.84 - 5.98) | 1.89 (1.2 - 2.96) | 12.15 (11.13 - 13.26) | 6.44 (4.93 - 8.42) | 3.89 (3.29 - 4.6) | 4.02 (3.59 - 4.51) | 3.44 (2.53 - 4.68) |
| Vomiting | 0.6 (0.25 - 1.45) | 1.2 (0.92 - 1.56) | 2.58 (2.37 - 2.82) | 1.98 (1.58 - 2.48) | 1.1 (0.96 - 1.28) | 2.22 (2.07 - 2.38) | 1.59 (1.29 - 1.97) |
| Drug interaction | 0.36 (0.05 - 2.53) | 0.06 (0.01 - 0.45) | 6.54 (5.95 - 7.2) | 2.95 (2.15 - 4.04) | 1.63 (1.33 - 2) | 0.87 (0.72 - 1.06) | 0.05 (0.01 - 0.38) |
| Decreased appetite | 0.23 (0.03 - 1.64) | 0.45 (0.25 - 0.81) | 3.59 (3.24 - 3.98) | 4.25 (3.44 - 5.26) | 2.35 (2.04 - 2.7) | 2.18 (1.97 - 2.4) | 1.8 (1.37 - 2.37) |
| Electrocardiogram qt prolonged |  |  | 17.38 (15.4 - 19.62) | 12.49 (9.07 - 17.18) | 5.94 (4.75 - 7.43) | 1.43 (1.04 - 1.96) | 0.23 (0.03 - 1.64) |
| Agitation | 3.06 (1.15 - 8.17) | 1.22 (0.63 - 2.35) | 8.31 (7.35 - 9.39) | 6.01 (4.35 - 8.31) | 14.87 (13.44 - 16.46) | 6.56 (5.91 - 7.28) | 1.94 (1.21 - 3.13) |
| Somnolence | 1.12 (0.42 - 3) | 1.25 (0.84 - 1.85) | 2.83 (2.49 - 3.21) | 2.81 (2.11 - 3.74) | 6.83 (6.24 - 7.48) | 2.16 (1.94 - 2.42) | 2.11 (1.6 - 2.78) |
| Delirium | 1.63 (0.23 - 11.6) | 3.77 (2.19 - 6.5) | 16.29 (14.33 - 18.53) | 12.51 (9.01 - 17.37) | 7.36 (5.98 - 9.05) | 6.79 (5.85 - 7.89) | 4.4 (2.77 - 6.99) |
| Aggression | 3.38 (1.09 - 10.51) | 0.6 (0.19 - 1.86) | 10.54 (9.23 - 12.03) | 7.18 (5.01 - 10.28) | 13.13 (11.53 - 14.95) | 9.12 (8.19 - 10.15) | 1.35 (0.67 - 2.69) |
| Hallucination | 2.27 (0.73 - 7.04) | 1.74 (1.01 - 3) | 6.98 (6.11 - 7.98) | 4.97 (3.49 - 7.07) | 8.59 (7.53 - 9.79) | 7.77 (7.06 - 8.54) | 3.28 (2.28 - 4.72) |
| Sinus bradycardia |  |  | 54.03 (47.2 - 61.84) | 9.82 (4.91 - 19.66) | 3.18 (1.76 - 5.74) | 4.97 (3.58 - 6.9) |  |
| Hypotension | 0.56 (0.14 - 2.25) | 2.36 (1.77 - 3.15) | 2.38 (2.08 - 2.74) | 1.31 (0.86 - 1.99) | 1.15 (0.93 - 1.43) | 1.16 (1 - 1.34) | 1.1 (0.75 - 1.61) |
| Loss of consciousness | 1.32 (0.43 - 4.1) | 2.67 (1.9 - 3.73) | 3.69 (3.21 - 4.25) | 5.36 (4.12 - 6.95) | 2.97 (2.51 - 3.52) | 2.64 (2.33 - 2.98) | 0.66 (0.35 - 1.22) |
| Toxicity to various agents |  |  | 2.52 (2.19 - 2.9) | 1.75 (1.2 - 2.55) | 0.44 (0.31 - 0.63) | 0.4 (0.31 - 0.52) |  |
| Nightmare |  |  | 10.39 (8.86 - 12.2) | 6.21 (3.91 - 9.86) | 2.35 (1.63 - 3.38) | 2.63 (2.07 - 3.34) | 0.97 (0.36 - 2.59) |
| Mania |  | 1.22 (0.3 - 4.88) | 20.02 (16.92 - 23.68) | 10.93 (6.59 - 18.15) | 4.97 (3.45 - 7.16) | 1.39 (0.86 - 2.23) |  |
| Dehydration | 2.54 (1.14 - 5.68) | 1.13 (0.68 - 1.87) | 2.44 (2.06 - 2.88) | 1.79 (1.15 - 2.78) | 1.64 (1.32 - 2.05) | 1.95 (1.7 - 2.25) | 1.07 (0.67 - 1.73) |
| Rhabdomyolysis |  |  | 7.52 (6.33 - 8.92) | 6.34 (4.17 - 9.63) | 2.37 (1.7 - 3.3) | 0.64 (0.42 - 1) | 1.22 (0.55 - 2.71) |
| Hyperhidrosis | 0.88 (0.22 - 3.51) | 2.65 (1.89 - 3.72) | 2.4 (2.02 - 2.85) | 1.67 (1.05 - 2.65) | 1.11 (0.85 - 1.46) | 1.63 (1.39 - 1.9) | 0.98 (0.59 - 1.63) |
| Depressed level of consciousness | 1.42 (0.2 - 10.11) | 1.01 (0.38 - 2.69) | 7.7 (6.48 - 9.16) | 9.08 (6.34 - 13) | 5.48 (4.38 - 6.86) | 3.39 (2.79 - 4.13) | 0.43 (0.11 - 1.7) |
| Pleurothotonus |  |  | 345 (283.78 - 419.41) | 200.33 (120.17 - 333.96) | 6.18 (1.54 - 24.75) | 30.07 (19.3 - 46.84) |  |
| Torsade de pointes |  |  | 34.75 (28.87 - 41.82) | 13.7 (7.12 - 26.35) | 4.66 (2.7 - 8.02) | 1.36 (0.68 - 2.73) |  |
| Neuroleptic malignant syndrome |  |  | 22.68 (18.83 - 27.3) | 9.06 (4.71 - 17.43) | 3.79 (2.32 - 6.19) | 2.37 (1.55 - 3.64) |  |
| Delusion |  | 1.94 (0.62 - 6.01) | 17.01 (14.11 - 20.51) | 9.26 (5.25 - 16.31) | 9.65 (7.37 - 12.64) | 5.55 (4.34 - 7.1) | 2.72 (1.13 - 6.54) |
| Lethargy | 3.94 (1.48 - 10.51) | 2.09 (1.19 - 3.69) | 4.48 (3.71 - 5.41) | 1.04 (0.43 - 2.49) | 5.9 (4.93 - 7.06) | 1.17 (0.88 - 1.54) | 4.27 (2.97 - 6.15) |
| Dysphagia | 1.14 (0.28 - 4.56) | 0.5 (0.21 - 1.21) | 1.33 (1.02 - 1.73) | 4.72 (3.44 - 6.47) | 1.82 (1.42 - 2.32) | 3.63 (3.22 - 4.09) | 0.34 (0.13 - 0.91) |
| Arrhythmia |  | 0.21 (0.03 - 1.49) | 2.73 (2.09 - 3.55) | 9.6 (6.98 - 13.22) | 1.3 (0.86 - 1.98) | 3.62 (3.04 - 4.31) | 0.18 (0.02 - 1.26) |
| Renal impairment | 0.68 (0.1 - 4.8) |  | 0.9 (0.64 - 1.27) | 5.17 (3.73 - 7.18) | 1.78 (1.36 - 2.34) | 0.79 (0.59 - 1.04) | 0.2 (0.05 - 0.81) |
| Cardiac failure |  | 0.25 (0.06 - 0.99) | 1.23 (0.91 - 1.66) | 4.77 (3.37 - 6.75) | 1.5 (1.11 - 2.03) | 2.59 (2.21 - 3.03) | 1.05 (0.56 - 1.95) |
| Fracture |  | 2.13 (0.8 - 5.67) | 2.37 (1.51 - 3.72) | 14 (9.2 - 21.28) | 2.69 (1.69 - 4.27) | 5.21 (4.14 - 6.56) | 4.48 (2.41 - 8.34) |
| Hypokalaemia |  |  | 4.46 (3.62 - 5.51) | 5.2 (3.35 - 8.07) | 0.79 (0.46 - 1.37) | 0.44 (0.26 - 0.72) | 0.55 (0.18 - 1.71) |
| Altered state of consciousness |  |  | 9.22 (7.44 - 11.43) | 10.56 (6.73 - 16.57) | 6.15 (4.62 - 8.19) | 4.05 (3.18 - 5.17) | 2.35 (1.05 - 5.23) |
| Sedation |  |  | 4.8 (3.66 - 6.28) | 8.27 (5.21 - 13.14) | 5.84 (4.47 - 7.63) | 1.39 (0.95 - 2.03) | 0.97 (0.31 - 3.01) |
| Circulatory collapse |  |  | 13.43 (11 - 16.4) | 11.88 (7.38 - 19.14) | 1.15 (0.55 - 2.41) | 3.53 (2.63 - 4.73) | 0.49 (0.07 - 3.5) |
| Abnormal behaviour |  | 1.5 (0.68 - 3.35) | 6.1 (5.02 - 7.4) | 5.09 (3.16 - 8.2) | 10.51 (8.94 - 12.36) | 8.16 (7.19 - 9.27) | 1.48 (0.7 - 3.1) |
| Hyponatraemia | 1 (0.14 - 7.13) | 0.71 (0.27 - 1.9) | 2.39 (1.84 - 3.1) | 3.62 (2.25 - 5.82) | 4.32 (3.49 - 5.33) | 1.1 (0.82 - 1.46) | 1.35 (0.7 - 2.6) |
| Asthenia | 0.3 (0.07 - 1.2) | 0.77 (0.54 - 1.11) | 1.14 (0.99 - 1.32) | 1.27 (0.93 - 1.74) | 2.5 (2.24 - 2.78) | 1.55 (1.41 - 1.7) | 1.85 (1.49 - 2.3) |
| Condition aggravated |  | 0.07 (0.02 - 0.26) | 1.6 (1.39 - 1.83) | 1.31 (0.93 - 1.84) | 2.42 (2.14 - 2.74) | 1.49 (1.34 - 1.66) | 0.08 (0.03 - 0.26) |
| Constipation |  | 0.23 (0.1 - 0.56) | 0.63 (0.49 - 0.82) | 0.94 (0.58 - 1.51) | 3.09 (2.72 - 3.52) | 0.62 (0.51 - 0.75) | 0.16 (0.06 - 0.41) |
| Gait disturbance | 3.2 (1.77 - 5.8) | 1.08 (0.7 - 1.66) | 1.27 (1.05 - 1.54) | 1.59 (1.08 - 2.34) | 3.03 (2.65 - 3.48) | 2.49 (2.24 - 2.76) | 2.3 (1.76 - 3.02) |
| Balance disorder | 3.27 (1.36 - 7.88) | 2.32 (1.5 - 3.6) | 1.72 (1.34 - 2.2) | 2.49 (1.57 - 3.95) | 3.59 (2.98 - 4.33) | 1.97 (1.66 - 2.35) | 3.23 (2.29 - 4.55) |
| Pulmonary embolism | 1.18 (0.3 - 4.74) | 0.31 (0.1 - 0.98) | 0.39 (0.24 - 0.64) | 0.88 (0.42 - 1.84) | 3.16 (2.62 - 3.83) | 0.63 (0.47 - 0.85) | 1.24 (0.73 - 2.09) |
| Hypersomnia | 1.98 (0.28 - 14.04) | 4.21 (2.39 - 7.43) | 1.73 (1.13 - 2.65) | 0.84 (0.21 - 3.34) | 9.71 (7.96 - 11.84) | 1.83 (1.34 - 2.51) | 3.84 (2.23 - 6.63) |
| Myoclonus |  |  | 16.32 (13.13 - 20.29) | 13.05 (7.57 - 22.5) | 23.13 (18.93 - 28.26) | 2.25 (1.45 - 3.49) | 0.71 (0.1 - 5.02) |
| Urinary incontinence | 5.72 (1.84 - 17.76) | 1.35 (0.51 - 3.6) | 7.82 (6.41 - 9.53) | 6.05 (3.64 - 10.04) | 8.96 (7.32 - 10.98) | 4.17 (3.4 - 5.12) | 2.56 (1.33 - 4.93) |
| Dyskinesia |  | 0.49 (0.12 - 1.98) | 4.78 (3.85 - 5.94) | 4.73 (2.89 - 7.72) | 5.86 (4.73 - 7.26) | 2.32 (1.83 - 2.93) | 1.46 (0.7 - 3.06) |
| Speech disorder | 3.24 (1.04 - 10.06) | 2.3 (1.3 - 4.05) | 3.06 (2.41 - 3.88) | 2.28 (1.23 - 4.24) | 4.15 (3.31 - 5.19) | 6.35 (5.6 - 7.19) | 1.29 (0.64 - 2.58) |
| Application site erythema |  |  | 0.81 (0.42 - 1.55) |  |  | 22.65 (20.59 - 24.92) |  |
| Application site pruritus |  |  | 1.56 (0.94 - 2.59) |  |  | 18.52 (16.54 - 20.73) |  |
| Urinary tract infection | 3 (1.56 - 5.79) | 0.41 (0.2 - 0.86) | 0.55 (0.41 - 0.75) | 0.84 (0.48 - 1.48) | 2.16 (1.81 - 2.56) | 2.4 (2.14 - 2.69) | 1.24 (0.84 - 1.84) |
| General physical health deterioration | 2.46 (1.02 - 5.92) | 0.26 (0.08 - 0.81) | 1.33 (1.04 - 1.7) | 1.35 (0.78 - 2.32) | 2.01 (1.62 - 2.49) | 3.11 (2.76 - 3.51) | 0.29 (0.11 - 0.78) |
| Parkinson's disease | 2.94 (0.41 - 20.91) | 1.04 (0.26 - 4.18) | 3.31 (2.27 - 4.84) | 4.98 (2.49 - 9.97) | 1.61 (0.89 - 2.91) | 16.1 (14.12 - 18.36) | 0.44 (0.06 - 3.12) |
| Gait inability | 1.95 (0.49 - 7.79) | 0.34 (0.09 - 1.38) | 1.01 (0.68 - 1.5) | 1.65 (0.82 - 3.29) | 2.38 (1.79 - 3.14) | 4.78 (4.17 - 5.49) | 0.87 (0.39 - 1.94) |
| Cardiac arrest |  | 0.96 (0.48 - 1.93) | 2.64 (2.15 - 3.23) | 1.58 (0.87 - 2.85) | 1.18 (0.85 - 1.65) | 2.91 (2.51 - 3.37) | 0.2 (0.05 - 0.81) |
| Femur fracture |  | 0.32 (0.05 - 2.29) | 1.14 (0.69 - 1.89) | 1.15 (0.37 - 3.58) | 0.18 (0.05 - 0.72) | 6.83 (5.84 - 8) | 1.91 (0.91 - 4) |
| Application site rash |  |  | 1.2 (0.57 - 2.51) |  |  | 14.1 (11.95 - 16.63) |  |
| Cardio-respiratory arrest |  |  | 2.12 (1.54 - 2.91) | 2.54 (1.32 - 4.89) | 1.06 (0.65 - 1.74) | 4.24 (3.57 - 5.02) |  |
| Restlessness |  | 0.82 (0.26 - 2.54) | 5.28 (4.25 - 6.56) | 4.57 (2.7 - 7.72) | 4.15 (3.18 - 5.42) | 4.81 (4.05 - 5.71) | 1.84 (0.92 - 3.69) |
| Fatigue | 0.87 (0.49 - 1.54) | 0.85 (0.67 - 1.08) | 0.56 (0.49 - 0.65) | 0.32 (0.21 - 0.49) | 1.08 (0.97 - 1.22) | 0.44 (0.39 - 0.49) | 2.4 (2.1 - 2.75) |
| Pyrexia | 0.16 (0.02 - 1.15) | 0.61 (0.4 - 0.93) | 0.45 (0.35 - 0.57) | 0.59 (0.36 - 0.94) | 0.51 (0.4 - 0.65) | 0.79 (0.69 - 0.91) | 4.32 (3.72 - 5.03) |
| Influenza like illness |  | 1.09 (0.57 - 2.1) | 0.06 (0.01 - 0.23) |  | 0.14 (0.05 - 0.36) | 0.1 (0.04 - 0.22) | 7.74 (6.16 - 9.72) |
| Lacunar infarction | 23.03 (3.24 - 163.71) | 65.91 (40.29 - 107.83) | 3.84 (1.44 - 10.24) |  | 6.89 (3.09 - 15.35) | 5.48 (2.94 - 10.2) | 66.01 (42.01 - 103.74) |
| Brain fog |  | 8.98 (5.21 - 15.47) | 1.46 (0.76 - 2.8) |  | 1.35 (0.64 - 2.84) | 0.28 (0.09 - 0.86) | 11.06 (7.05 - 17.36) |

**Supplementary Table S16.** TTO overall Kruskal-Wallis rank-sum test.

| **test** | **comparison** | **statistic** | **df** | **p value** |
| --- | --- | --- | --- | --- |
| Kruskal-Wallis rank-sum test | TTO distributions across seven drugs | 480.23 | 6 | < 0.001 |

Supplementary Table S17. Significant post hoc pairwise TTO comparisons.

| **Drug 1** | **Drug 2** | **BH adjusted P** |
| --- | --- | --- |
| Rivastigmine | Memantine | < 0.001 |
| Memantine | Donepezil | < 0.001 |
| Memantine | Aducanumab | < 0.001 |
| Donanemab | Aducanumab | < 0.001 |
| Lecanemab | Aducanumab | < 0.001 |
| Memantine | Lecanemab | < 0.001 |
| Memantine | Galantamine | < 0.001 |
| Galantamine | Aducanumab | < 0.001 |
| Rivastigmine | Donanemab | < 0.001 |
| Rivastigmine | Lecanemab | < 0.001 |
| Donepezil | Aducanumab | < 0.001 |
| Memantine | Donanemab | < 0.001 |
| Rivastigmine | Aducanumab | < 0.001 |
| Rivastigmine | Donepezil | < 0.001 |
| Rivastigmine | Galantamine | 0.007 |
| Donepezil | Donanemab | 0.008 |
| Lecanemab | Donanemab | 0.008 |
| Lecanemab | Donepezil | 0.021 |

Note: Pairwise Wilcoxon rank-sum tests were performed only after the overall Kruskal-Wallis test. P values were adjusted using the Benjamini-Hochberg method. To keep the supplementary table compact, only comparisons with adjusted P < 0.05 are shown.

**Supplementary Table S18.** PT-level time-to-onset and Weibull analyses for the five most frequently reported preferred terms of each anti-Alzheimer drug.

| **PT** | **Valid TTO reports, n** | **Overall median time-to-onset (IQR)** | **Weibull β (95% CI); onset pattern** |
| --- | --- | --- | --- |
| **Lecanemab** |  |  |  |
| ARIA-E | 277 | 49 (35,76) | 0.750 (0.692–0.813); early-failure |
| Headache | 258 | 0 (0,1) | 0.413 (0.359–0.475); early-failure |
| ARIA-H | 261 | 49 (41,139) | 0.925 (0.846–1.012); random |
| Infusion related reaction | 219 | 0 (0,2) | 0.445 (0.380–0.520); early-failure |
| Chills | 156 | 0 (0,0) | 0.524 (0.394–0.697); early-failure |
| **Aducanumab** |  |  |  |
| ARIA-E | 116 | 168 (118.75,365) | 0.730 (0.644–0.828); early-failure |
| ARIA-H | 83 | 175 (123.5,320.5) | 0.951 (0.824–1.097); random |
| Headache | 26 | 85.5 (3.75,150) | 0.630 (0.455–0.872); early-failure |
| Confusional state | 21 | 72 (19,124) | 0.656 (0.472–0.913); early-failure |
| Cerebral haemorrhage | 8 | 152.5 (96.25,575.5) | 0.691 (0.412–1.157); random |
| **Donanemab** |  |  |  |
| ARIA-E | 94 | 34 (17,74.75) | 0.873 (0.755–1.011); random |
| ARIA-H | 82 | 29.5 (15,75) | 0.662 (0.572–0.768); early-failure |
| Infusion related reaction | 167 | 27 (0,37) | 0.735 (0.643–0.840); early-failure |
| Headache | 69 | 1 (0,40) | 0.476 (0.386–0.588); early-failure |
| Flushing | 96 | 0 (0,28) | 1.313 (1.052–1.639); wear-out/delayed |
| **Donepezil** |  |  |  |
| Bradycardia | 255 | 62 (2,508.5) | 0.535 (0.479–0.597); early-failure |
| Syncope | 224 | 263 (8.75,1029.5) | 0.536 (0.480–0.600); early-failure |
| Vomiting | 247 | 2 (0,35.5) | 0.391 (0.349–0.437); early-failure |
| Fall | 155 | 67 (3.5,353) | 0.511 (0.447–0.583); early-failure |
| Drug interaction | 110 | 66 (10,806.25) | 0.565 (0.482–0.664); early-failure |
| **Galantamine** |  |  |  |
| Fall | 16 | 67 (1.5,600.25) | 0.696 (0.439–1.103); random |
| Decreased appetite | 29 | 35 (7,360) | 0.522 (0.386–0.707); early-failure |
| Bradycardia | 23 | 28 (4.5,345) | 0.571 (0.402–0.810); early-failure |
| Loss of consciousness | 23 | 37 (3,228) | 0.616 (0.430–0.883); early-failure |
| Syncope | 16 | 19.5 (14.5,63) | 0.462 (0.330–0.646); early-failure |
| **Rivastigmine** |  |  |  |
| Fall | 266 | 76 (13.25,345.75) | 0.518 (0.471–0.569); early-failure |
| Vomiting | 277 | 18 (0,224) | 0.441 (0.398–0.489); early-failure |
| Confusional state | 196 | 36.5 (2,193.25) | 0.490 (0.437–0.550); early-failure |
| Cerebrovascular accident | 90 | 136.5 (40.25,447.75) | 0.628 (0.535–0.737); early-failure |
| Application site erythema | 95 | 50 (1,140) | 0.585 (0.494–0.694); early-failure |
| **Memantine** |  |  |  |
| Confusional state | 524 | 7 (0,30) | 0.524 (0.487–0.562); early-failure |
| Dizziness | 437 | 6 (0,31) | 0.508 (0.467–0.552); early-failure |
| Somnolence | 281 | 6 (0,26) | 0.515 (0.465–0.570); early-failure |
| Fall | 158 | 24 (1,184) | 0.523 (0.458–0.597); early-failure |
| Agitation | 195 | 7 (0,53.5) | 0.563 (0.500–0.633); early-failure |
